# Impact of oral health perception on the quality of life of hospital staff

**DOI:** 10.12688/f1000research.161146.1

**Published:** 2025-02-26

**Authors:** Miryam Lora Loza, Sheyla del Pilar Alvarado-Romero, Katia Ninozca Flores Ledesma, Nancy Cuenca Robles, David Rene Rodríguez Díaz

**Affiliations:** 1 Graduate School, César Vallejo University, Trujillo, La Libertad, 13001, Peru; 2 Graduate School of, Cesar Vallejo University, Lima, La Libertad, 13001, Peru; 3 School of Human Medicine, Private University of the North, Trujillo, Lima Norte, 13001, Peru

**Keywords:** Quality of life, perception, oral health, staff, hospital

## Abstract

**Introduction:**

The perception of oral health that hospital staff may have has repercussions on the general quality of life that they lead and the corresponding work performance. This work attempts to show how these two dimensions of the human being go hand in hand, which would provide the necessary knowledge to draw up strategies that can alleviate the aforementioned profile, and at the same time would help to build a work context that could well have a healthier horizon.

**Aim:**

To determine the relationship between quality of life (QoL) and oral health perception (OHP) in the staff of level II-1 hospital, located in the northern zone of Peru.

**Methods:**

The study had a quantitative approach, a cross-sectional design, an applied type and a correlational-causal scope. The participation consisted of 72 participants. Reference was made to the validated questionnaires OHIP-14 and HU-DBI, which allow measuring the quality of life and the perception of oral health, and obtained reliability coefficients of 0.847 and 0.804, respectively. Data processing was carried out by applying the Spearman correlation coefficient and ordinal logistic regression.

**Results:**

The analyses showed a significant association between quality of life and oral health perception (Rho=0.391, p<0.05), with an impact of 19.8% according to Nagelkerke’s pseudo R square. The dimensions of quality of life that most significantly influenced oral health perception were physical limitation (Rho=0.319, p<0.05) and social limitation (Rho=0.242, p<0.05). Regarding the general results, the excellent level of quality of life was the most prevalent (38.9%), while the low level of oral health perception was the most frequent, with 52.8%.

**Conclusion:**

The link between quality of life and the perception of oral health in hospital staff emphasizes the need to implement comprehensive strategies that optimize their well-being and work performance, contributing significantly to improving hospital services.

## Introduction

Oral health is an essential pillar for comprehensive well-being, as it has a direct impact on quality of life, since it allows essential functions such as communicating, eating and interacting socially, as well as strengthening self-esteem and relationships with other people. ^1^ Oral diseases not only affect functional aspects, but also psychological, social and economic dimensions, generating discomfort, pain and loss of self-esteem, which compromises general well-being and social relationships. ^2, 3^ The relationship between oral health and quality of life highlights its importance in meeting SDG 3: Good Health and Well-being, aimed at ensuring healthy conditions for all people. In addition, oral health contributes to other global goals, such as access to safe drinking water (SDG 6), reducing inequalities (SDG 10) and promoting sustainable communities (SDG 11).

The number of people affected by oral cavity diseases is close to 3.5 billion worldwide, being more frequent in low- and middle-income countries, which concentrate 75% of the cases. Among these, untreated dental caries is the most prevalent condition, reflecting inequalities in access to preventive services and basic treatments. ^4, 5^ In Latin America, periodontal diseases are considered an epidemic that significantly affects the population, and in countries such as Peru, the lack of priority in oral health is reflected in low public investment, with oral cancer rates of 2.60 per 100,000 women and 1.97 in men between 2000 and 2017. ^6^ This panorama is linked to the limited allocation of resources, the absence of effective preventive strategies and early detection programs. ^7^ In response to this problem, international organizations such as the WHO have launched key initiatives. The IED Vision 2030 Report and the 2021 Resolution on oral health emphasize the need to incorporate this area into Universal Health Coverage (UHC), aligning it with the global agenda for addressing non-communicable diseases (NCDs).

Likewise, the “World Oral Health Report 2022” highlights the interest in highlighting the importance of prioritizing the promotion of oral health, following national plans that are in line with global strategies that support it. This requires the collaboration of support agencies such as UNESCO and UNDP, which can be decisive in the integration of oral health into policies for the well-being and development of people. ^7– 9^ PSB is a reference aspect of health for the personnel who achieve it and the quality of the services that result from it. This perception may be influenced by some circumstances of the environment or of the personnel themselves: restricted access to dental services and long workloads. ^10– 12^ Having a negative perception of SO often hinders interaction and self-esteem, which shows the importance of not taking SO into account in CV studies regarding healthcare personnel. ^6, 13^

The present study aims to try to analyze the relationship between the QoL and the PSB of the staff of a level II-1 hospital, located in the north of Peru, taking into account its dimensions and interactions. The research aims to provide evidence to design strategies that promote the well-being of health personnel and optimize the quality of care provided to patients. ^14, 15^

## Methods

### Research type and design

An applied study was conducted, aimed at using theoretical knowledge to propose concrete solutions to problems of hospital staff. ^16, 17^ An applied study was conducted, aimed at applying theoretical knowledge to propose concrete solutions to problems of hospital staff. ^18^ The scope of the study was correlational-causal, seeking to identify the relationship between the variables and understand their level of causal relationship. ^19, 20^ Likewise, a non-experimental cross-sectional research design was used, collecting data at a single time without altering the variables, allowing the analysis of phenomena in their natural context.

### Population

The group was made up of professional and technical health personnel who provided care in a level II-1 hospital in northern Peru, reaching a total of 80 people. This group included 21 physicians, 10 obstetricians, 1 dentist, 1 pharmaceutical chemist, 17 nurses, 2 psychologists, 3 biologists, 5 microbiologists, 2 medical technologists, 13 nursing technicians, 4 pharmacy technicians and 1 laboratory technician. After applying the inclusion criteria, which took into consideration appointed or seconded personnel and CAS (Administrative Contracting of Services), with more than 6 months of seniority, who agreed to complete each of the questions formulated in both questionnaires, adding their stamp and signature on the document referring to informed consent, and the exclusion criteria considered workers with less than six months of seniority, those who were on vacation or on medical leave, those who worked under a third party modality, those who were performing the SERUMS (Rural and Marginal Urban Health Service) and those who did not agree to participate in the study. As a result, a final sample of 72 collaborators was obtained. The sampling used was non-probabilistic and based on convenience.

### Variables

Regarding the variables, quality of life (QoL) was identified as the independent variable, defined as the perception that an individual has about his or her life in aspects related to his or her family relationships, health and social environment, used to evaluate his or her general well-being. ^21^ To do so, various dimensions were analyzed, such as functional limitation, psychological discomfort, physical pain, various physical, psychological and social disabilities and handicap. On the other hand, the dependent variable was PSB, which refers to the subjective assessment of the oral state and its impact on QoL. ^22^ This variable was broken down into 3 main dimensions: perception of attitude, knowledge and behavior.

### Data collection technique and instrument

The technique used was the survey, since it allowed collecting precise information from the professional and technical staff of the hospital through questionnaires, without altering the environment or the object of study. ^23^

Regarding the instruments, the Quality of Life (QoL) Questionnaire was used, originally designed by Slade and Spencer in 1994, adapted by Espinoza in 2017, and updated and validated in 2024 by Cruzado, Alvarado, and Lora. Loza. This questionnaire includes 14 items organized into 7 dimensions (Functional limitation, Physical pain, Psychological discomfort, Physical disabilities, Psychological disabilities, Social disability, and Disability), with 2 items per dimension. A 5-point Likert scale was applied (0 = never, 4 = very frequently), the results of which were classified into three categories: excellent (0-2 points), average (3-9 points), and poor quality of life (10 points or more).

Similarly, the Oral Health Perception Questionnaire (OHQ) was used, initially developed by Kawamura in 1988, modified by Midolo in 2023, and validated in 2024 by Cruzado, Alvarado, and Lora. This instrument consists of 20 items distributed in three dimensions: Perception of knowledge (8 items), Perception of attitude (6 items), and Perception of behavior (6 items). Responses were recorded on a dichotomous scale (Yes = 1, No = 0), and results were classified into three levels: poor (0-9 points), average (10 points), and excellent (11-20 points).

Both instruments were subjected to strict validation, which was carried out by five experts in the area of health and research methodology, who evaluated the questions of both questionnaires with respect to the criteria of coherence, relevance, clarity including further clarifications and sufficiency. This process had a degree of concordance of 100% (Aiken’s V = 1.00), which indicates a strong validity in the instruments. And for reliability, a pilot study was carried out prior to the research, applying the Quality of Life (QoL) Questionnaire to 20 healthcare workers, where a Cronbach’s Alpha coefficient of 0.847 was obtained, which is a good internal consistency. In the case of the Oral Health Perception Questionnaire (OHQ), which was constructed as a dichotomous instrument, it showed good internal reliability with a Cronbach’s Alpha coefficient of 0.804, which also indicates high reliability. The results obtained support the suitability of the questionnaires to be applied in the research.

***Procedure***

The process began with the sending of a cover letter, issued by the Graduate School of the César Vallejo University; this was addressed to the administrative area of the level II-1 La Libertad hospital. In it, authorization was requested for the use of the research instruments. Once they authorized the use, days and times were coordinated to visit the hospital and distribute the questionnaires to the healthcare staff. Likewise, at the separation, the subjects were told the purposes and objectives of the study, guaranteeing the anonymity and confidentiality of the data. The participants were asked to read and sign an informed consent prior to answering the questionnaires. The application of the instruments was carried out in isolated spaces within their work areas, and lasts approximately 8 minutes per subject, ensuring adequate conditions to minimize distractions and biases.

***Information analysis***

The data collected through the application of the questionnaires have been statistically processed using the IBM SPSS software version 25 (Statistical Package for the Social Sciences, https://www.ibm.com/products/spss-statistics ). First, a normality test was performed with the Kolmogorov-Smirnov statistic, which yielded results indicating that the data did not comply with normality (p < 0.05); a result that precipitated the option of using non-parametric tests to analyze the data. To analyze the relationship between Quality of Life (QoL) and Oral Health Perception (OHP), the Spearman Rho correlation coefficient was used, which is the most appropriate for ordinal data or data that do not follow a normal distribution. Once the data were categorized, an ordinal logistic regression model was used, Nagelkerke’s Pseudo R square as a statistic. As an alternative to the software used, the R program, The R Project for Statistical Computing, could also be used. This analysis identified the impact of the independent variables and the amount of variability explained. The results obtained allowed an in-depth analysis of the associations and effects of the variables. ^24– 27^

### Ethical implications

This study was conducted under strict principles of scientific integrity and in compliance with the Code of Ethics for Research of the César Vallejo University (Universidad César Vallejo, 2024). ^28^ Likewise, international ethical standards were rigorously respected, including the provisions of the Council of International Organizations of Medical Sciences (CIOMS, 2016), the guidelines of the Belmont Report (National Commission for the Protection of Human Subjects of Biomedical and Behavioral Research, 1979) and the principles of the Declaration of Helsinki (World Medical Association, 2013). ^29– 31^ The study was approved by the Research Ethics Committee of the Master’s Program in Health Services Management at César Vallejo University, through the Resolution of the Vice-rectorate for Research No. 405-2024-VI-UCV, issued on January 30, 2025, with official confirmation on February 11, 2025. The ethics committee was made up of: president: Dr. Roberto Santiago Bellido García, secretary: Dr. Diaz Mujica Juana Yris, External member: Mgtr. Melecio Belisario Carlos Reyes, Alternate member: Dr. Patricia Luz Figueroa Garrido, Added member: Mtro. Omar Arturo Lizárraga Carrasco, Added member: Dr. Meneses La Riva Mónica Elisa. Likewise, all participants signed a written informed consent, in which the objectives, procedures, benefits, and risks of the study were detailed. Only those who fully understood and accepted these conditions participated. Furthermore, the university’s ethical policies were respected, ensuring the originality and rigor of the work, with a level of textual similarity of less than 17% in the documents generated.

## Results

Table 1, shows that 38.90% of the staff with excellent CV reported a low PSB of 52.80%, while 34.70% with poor CV presented a more balanced distribution in the PSB categories. Also, a low positive correlation (r=0.391) but significant (p=0.001) was evident between CV and PSB. Also, the ordinal logistic regression analysis, with a Nagelkerke pseudo R square of 0.198 (p=0.001), confirmed a significant influence of CV on PSB, representing 19.80%.

**Table 1.** Causal relationship between Quality of life and the Perception of Oral Health of the healthcare staff of a level II-1 hospital in Northern Peru, 2024.

Quality of life (CV)	Oral Health Perception (OHP)						Total	
	Low		Regular		Excellent			
	N	%	N	%	N	%	N	%
**Excellent**	21	29,20	6	8.30	1	1.40	28	38.90
**Regular**	8	11,10	1	1.40	10	13.90	19	26.40
**Bad**	9	12.50	6	8.30	10	13.90	25	34.70
**Total**	38	52.80	13	18,10	21	29,20	72	100,00

*Note:* Correlation between quality of life and oral health, data processed with SPSS, own elaboration.

Table 2 the results show that QoL is mainly classified into three levels: excellent (38.90%), poor (34.70%) and average (26.40%). Furthermore, when analyzing the seven dimensions of QoL, it can be observed that all of them reached high percentages at the excellent level. Firstly, Functional Limitation obtained 44.40%, followed by Physical Pain which obtained 52.80%. Likewise, Psychological Discomfort reached 54.20%, while Physical and Psychological Disability both registered 69.40%. On the other hand, Social Disability reached 76.40% and, finally, Handicap reached the highest percentage with 83.30%.

**Table 2.** Quality of Life Level and its dimensionsof the healthcare staff of a Level II-1 Hospital in Northern Peru, 2024.

Rho Spearman	Next.	Pseudo R Nagelkerke	Next.
0.391	0.001	0.198	0.001

CV			CV Dimensions													
			Limitation functional		Pain physical		Discomfort psychological		Physical disability		Inability psychological		Social incapacity		Handicap	
Levels	N	%	N	%	N	%	N	%	N	%	N	%	N	%	N	%
**Excellent**	28	38.90	32	44.40	38	52.80	39	54.20	50	69.40	50	69.40	55	76.40	60	83.30
**Regular**	19	26.40	28	38.90	23	31.90	22	30.60	18	25.00	18	25.00	14	19.40	10	13.90
**Bad**	25	34.70	12	16.70	11	15.30	11	15.30	4	5.60	4	5.60	3	4.20	2	2.80
**Total**	72	100.00	72	100.00	72	100.00	72	100.00	72	100.00	72	100.00	72	100.00	72	100.00

*Note:* Statistical analysis of oral health perception, processed questionnaires, own elaboration.

Table 3 indicates that the Oral Health Perception (OHP) is mainly distributed in three levels: low (52.80%), excellent (29.20%) and regular (18.00%). When examining the different dimensions, it was identified that the Knowledge Perception registered 100% at a low level. On the other hand, the Attitude Perception showed 44.4% at the low level, while the Behavior Perception was located at 62.5% at the regular level.

**Table 3.** Level of Perception of Oral Health and its dimensions in healthcare personnelfrom a level II-1 hospital in Northern Peru, 2024.

PSB			PSB Dimensions					
			Perception of knowledge		Attitude perception		Perception of behavior	
Levels	N	%	N	%	N	%	N	%
**Low**	38	52.80	72	100.00	32	44.40	15	20.80
**Regular**	13	18.00	0	0.00	31	43.10	45	62.50
**Excellent**	21	29.20	0	0.00	9	12.50	12	16.70
**Total**	72	100.00	72	100.00	72	100.00	72	100.00

*Note:* Statistical analysis of oral health perception, processed questionnaires, own elaboration.

Table 4 presents a detailed analysis that shows the existing relationships between the dimensions of Quality of Life (QoL) and the Perception of Oral Health (POH), highlighting important differences in the magnitude of these associations. In particular, the dimension corresponding to psychological distress showed the most significant connection with POH, supported by a moderate correlation coefficient (r = 0.421) and a solid level of statistical significance (p = 0.000). Likewise, Nagelkerke’s Pseudo R square obtained a value of 0.111 (p = 0.027), which explains that this dimension predicts 11.1% of the variability of POH. In turn, physical disability showed a significant association with POH, although with a smaller effect size than the previous dimension. The correlation coefficient was 0.319 (p = 0.006), while the value of the Pseudo R square was 0.167 (p = 0.004), indicating a 16.7% effect on PSB. In contrast, the dimension associated with functional limitations indicated a much weaker relationship with PSB, with a correlation coefficient of 0.096 (p = 0.424) and a Pseudo R square of 0.014 (p = 0.649), suggesting a practically null effect on the target variable.

**Table 4.** Relationship between the dimensions of theQuality of life with the perception of oral healthof the staff of a level II-1 hospital in La Libertad, 2024.

Functional limitation	Oral Health Perception								Inferential analysis			
	Low		Regular		Excellent		Total		Rho Spearman	Next.	Pseudo R Nagelkerke	Next.
	N	%	N	%	N	%	N	%				
Excellent	18	25.00	7	9.70	7	9.70	32	44.40	0.096	0.424	0.014	0.649
Regular	15	20.80	3	4.20	10	13.90	28	38.90				
Bad	5	6.90	3	4.20	4	5.60	12	16.70				
**Total**	38	52.80	13	18.10	21	29.20	72	100.00				

Pain physical	PSB						Total					
	Low		Regular		Excellent							
	N	%	N	%	N	%	N	%				
Excellent	24	33.30	7	9.70	7	9.70	38	52.80	0.266	0.024	0.093	0.049
Regular	12	16.70	2	2.80	9	12.50	23	31.90				
Bad	2	2.80	4	5.60	5	6.90	11	15.30				
**Total**	38	52.80	13	18.10	21	29.20	72	100.00				

Discomfort psychological	PSB						Total					
	Low		Regular		Excellent							
	N	%	N	%	N	%	N	%				
Excellent	26	36.10	6	8.30	7	9.70	39	54.20	0.421	0.000	0.111	0.027
Regular	8	11.10	5	6.90	9	12.50	22	30.60				
Bad	4	5.60	2	2.80	5	6.90	11	15.30				
**Total**	38	52.80	13	18.10	21	29.20	72	100.00				

Physical disability	PSB						Total					
	Low		Regular		Excellent							
	N	%	N	%	N	%	N	%				
Excellent	30	41.70	8	11.10	12	16.70	50	69.40	0.319	0.006	0.167	0.004
Regular	8	11.10	5	6.90	5	6.90	18	25.00				
Bad	0	0.00	0	0.00	4	5.60	4	5.60				
**Total**	38	52.80	13	18.10	21	29.20	72	100.00				

Inability psychological	PSB						Total					
	Low		Regular		Excellent							
	N	%	N	%	N	%	N	%				
Excellent	30	41.70	8	11.10	12	16.70	50	69.40	0.232	0.050	0.167	0.004
Regular	8	11.10	5	6.90	5	6.90	18	25.00				
Bad	0	0.00	0	0.00	4	5.60	4	5.60				
**Total**	38	52.80	13	18.10	21	29.20	72	100.00				

Inability social	PSB						Total					
	Low		Regular		Excellent							
	N	%	N	%	N	%	N	%				
Excellent	31	43.10	11	15.30	13	18.10	55	76.40	0.242	0.040	0.124	0.017
Regular	7	9.70	2	2.80	5	6.90	14	19.40				
Bad	0	0.00	0	0.00	3	4.20	3	4.20				
**Total**	38	52.80	13	18.10	21	29.20	72	100.00				

Handicap	PSB						Total					
	Low		Regular		Excellent							
	N	%	N	%	N	%	N	%				
Excellent	36	50.00	9	12.50	15	20.80	60	83.30	0.298	0.011	0.131	0.013
Regular	2	2.80	4	5.60	4	5.60	10	13.90				
Bad	0	0.00	0	0.00	2	2.80	2	2.80				
**Total**	38	52.80	13	18.10	21	29.20	72	100.00				

*Note:* Association between dimensions of quality of life and oral health, analysis in SPSS, own elaboration.

Overall, these findings confirm that a positive relationship was found between the dimensions of quality of life and oral health problems. However, it is clear that the dimensions of psychological distress and physical disability have a greater influence on PSB, which underlines the need to implement specific interventions targeting these critical areas.

## Discussion

Quality of life (QoL) and oral health perception (OHP) are essential elements that directly influence the general well-being of hospital staff. In this study, the relationship between these variables was established in a total of 72 workers from a hospital located in the northern part of Peru during the year 2024, with the purpose of identifying and establishing areas of opportunity that allow the execution of strategies that address the specific needs of the group. First, the data presented in Table 1 show that there is a positive correlation of low level (r = 0.391), but statistically significant (p < 0.05). In addition, it was shown that QoL influences OHP by 19.8%. It should be noted that the highest frequency of cases corresponds to healthcare personnel who reported an optimal QoL, despite experiencing low levels of OHP (29.2%). These findings show similarities with those obtained by Miranda and Alcocer in 2021, who found that the sociodemographic characteristics of older adults intervene in the perception of QoL and its link with oral health, without generating significant negative impacts. In their study, it was identified that a high percentage of participants maintained outstanding (45.4%) or moderate (34.6%) levels of QoL. Thus, when health professionals experience a favorable QoL status, problems associated with oral health tend to go unnoticed, since they do not significantly interfere with their daily activities or their work performance. ^2, 10^

Table 2 shows that quality of life (QoL) is distributed into three main categories: excellent with 38.9%, poor with 34.7% and average with 26.4%. In addition, when analyzing the seven dimensions of QoL, it is observed that all of them reached high percentages at the excellent level. Firstly, Functional Limitation obtained 44.40%, followed by Physical Pain which obtained 52.80%. Likewise, Psychological Discomfort reached 54.20%, while Physical and Psychological Disability both registered 69.40%. On the other hand, Social Disability reached 76.40% and, finally, Handicap reached the highest percentage with 83.30%. TheseData show similarity with those obtained by Espinoza et al. (2022), who conducted a study on QoL and its link to the oral health of members of a geriatric center in Lima. Their findings revealed that QoL was excellent in 66.8% of cases and that oral health did not negatively impact this perception. ^32^ These results can be explained by the importance that health personnel attach to the care of their oral health, which is reflected in a positive perception of their QoL. Theory suggests that good oral health is closely related to a better quality of life, as it reduces pain and discomfort, and improves functional and social capacity. ^33^

Table 3 presents the most relevant data on PSB and its dimensions in the healthcare staff of a hospital in the north of Peru. PSB was mainly distributed in three levels: low (52.8%), excellent (29.2%) and regular (18.0%). Likewise, the specific dimensions when analyzed, showed that the low level predominated at 100% regarding Knowledge Perception. For its part, Attitude Perception registered 44.4% also at a low level. Finally, Behavior Perception was mostly located at the regular level with 62.5%. These findings differ considerably from previous studies, such as that of López (2021), who indicated that the level of knowledge about oral health in healthcare workers at Hospital II EsSalud during the Covid-19 pandemic was high, reaching 81.5%, which shows a good command of this dimension in this context. ^34^ Therefore, the limited perception of knowledge could be linked to the lack of constant training programs in oral health (OH) aimed at health personnel. This is in line with previous studies that highlight that training and education are essential elements to strengthen knowledge and promote adequate OH practices. On the other hand, attitude and behavior not only reflect the degree of knowledge, but also the cultural beliefs and traditions related to OH, which can influence the adoption of preventive habits and the search for appropriate treatments. ^35, 36^

Table 4 presents the results on the relationship between functional limitation and oral health problems (OHP) in workers. It was observed that the highest percentage corresponds to those with an excellent quality of life (QoL), but with low OHP, representing 25.0%. However, statistical analysis showed that there is no significant correlation (r = 0.096) or a relevant influence between this dimension and the variable (p > 0.05). When comparing these results with previous research, similar findings are identified. Like García-Cortés et al. (2020) reported that OHP did not show a statistically significant relationship with QoL in a study conducted with health professionals in Spain. ^37^ Similarly, López-Jiménez et al. (2019) reported that, although functional limitations existed, these did not significantly affect OHP in a sample of nurses in Mexico. ^38^ On the other hand, some studies have found a significant relationship between these variables in different contexts. For example, Martínez-Rodríguez et al. (2018) found that functional limitations did affect PSB in a sample of older adults in Chile. ^39^

Regarding the influence of physical pain and oral health problems (OHP) in workers, a low intensity positive correlation was identified (r = 0.266), with a limited influence of 9.3% of this dimension on OHP. Likewise, the highest percentage was found in workers with excellent CV, but with low OHP (33.30%). These data are similar to the research by Campos et al. (2014) who conducted an analysis of how job performance is affected by alterations in the oral cavity and that in relation to physical pain, this had a negative influence of 82.90%. ^40^ This indicates that the absence of physical pain attributed to alterations in the oral cavity can lead to OHP going unnoticed. ^41^

Similarly, when evaluating the link between psychological distress and PSB in workers, a moderate correlation (r = 0.421) was observed with a high level of statistical significance (p < 0.01) and a relevant influence of 11.1% of this dimension on PSB. The highest percentage belongs to workers with excellent CV, but with low PSB (36.10). These results are similar to those found by Espinoza (2017), who identified a negative impact of 61.4% in relation to psychological distress. ^42^ This suggests that the absence of psychological distress related to the oral cavity may lead to PSB not being considered. ^41^

Regarding the link between physical disability and PSB in workers, a significant correlation of low magnitude was observed (r = 0.319, p < 0.05), with a notable influence of 16.7% of this dimension on the Perception of Oral Health (PSB). These findings contrast with the results of Bellamy and Moreno (2014), who reported that physical disability was one of the most affected dimensions in patients with removable prostheses and tooth loss, reaching 32.5%. ^43^ Thus, when physical disability is absent, PSB is unnoticed; and, on the contrary, if this dimension is present, SB is prioritized. ^44^

Likewise, in relation to psychological incapacity and PSB of workers, a low correlation was identified (r = 0.232) with a significant influence of 16.7% on PSB. In addition, the highest percentage corresponded to those workers with an excellent quality of life and low PSB (41.7%). Similarly, when psychological incapacity ^42^ is at a regular level, PSB is located at regular or excellent levels, with 6.9% in both cases. These findings agree with what was reported by Espinoza (2017), who showed a negative influence of oral health on QoL, reflecting an impact of 31.5% in relation to psychological incapacity. In this sense, when workers do not present psychological incapacity, PSB is usually not a priority. In contrast, when there is psychological incapacity, this favors the adoption of actions that directly influence oral health. ^44^

Regarding the link between social disability and PSB of the hospital’s care workers, a significant correlation (p<0.05) was found, low (r = 0.242) and a significant influence of 12.4% of the dimension on PSB. On the other hand, the highest percentage was reached by workers with excellent QoL, but with low PSB (43.10%). Likewise, if the social disability is of a bad level, the PSB is exclusively outstanding (4.20%). These data can be contrasted with that evidenced by Espinoza (2017), who revealed the negative association of Oral Health in QoL, with a negative impact of 23.40% regarding social disability. ^42^ Therefore, when workers do not present social disability, PSB is not considered important; otherwise, if workers present social disability, this favors practical behaviors that influence SO. ^45^

Finally, regarding the link between disability and workers’ PSB, a significant correlation of low magnitude was identified (r = 0.298, p < 0.05), with a 13.1% influence of this dimension on the Perception of Oral Health (PSB). In addition, the highest percentage corresponded to workers with excellent quality of life and low PSB, reaching 50.0%. Similarly, when disability is present at moderate levels, PSB was distributed between excellent and regular, with 5.6% in each category. These data are similar to those reported by Espinoza et al. (2022), who showed a negative link between oral health and QoL, reflecting an impact of 17.0% in relation to disability. ^32^ Thus, when workers do not show signs of disability, PSB is often left in the background. On the contrary, in those cases where there is a disability, this encourages the adoption of specific actions and practices that have a direct impact on oral health. ^45^

### Limitations of the study

Although the present study provides relevant information on the relationship between Quality of Life (QoL) and the perception of Oral Health Perception (OHP) in hospital care staff, it is important to consider certain limitations. Firstly, the cross-sectional approach characteristic of exploratory studies such as this one restricts the ability to identify causal relationships between variables. However, the results present a good basis for further longitudinal research, since they could assess how these relationships vary over time.

Second, although the sample is representative of a level II-1 hospital in northern Peru, replicating this study in other institutions and other locations around the country could increase the generalizability of the results and enhance the identification of common patterns in different contexts.

Finally, self-assessment based on questionnaires, although validated and reliable, is subject to the subjective factors of the participant. However, the application of standardized and widely recognized instruments favors the comparability and validity of the data reported.

### Implications of the study

The findings highlight the consideration of oral health as one of the central components of the comprehensive well-being of hospital care staff. The significant correlation between QoL and PSB, the influence of certain dimensions such as physical disability and psychological disability, among others, highlights the need for interventions that can jointly address oral health and psychosocial factors, since these could help to perceive personal well-being, as well as the quality of the service offered to patients. Finally, the results have global implications, becoming the need for multidimensional and collaborative approaches in hospital health spaces that act on sustainable development and well-being.

## Conclusion

The study shows a significant correlation between Quality of Life (QoL) and Oral Health Perception in healthcare workers at a level II-1 hospital located in the north of Peru, with Spearman’s correlation coefficient of 0.391 and Nagelkerke’s Pseudo R squared of 0.198, both with significance at a p level of 0.001. Higher quality of life has a significant relationship with a more favorable oral health perception. In addition, significant correlations were found in the dimensions of physical disability (Rho = 0.319, p < 0.05, Nagelkerke = 0.167), psychological (Rho = 0.232, p = 0.05, Nagelkerke = 0.167), social (Rho = 0.242, p < 0.05, Nagelkerke = 0.124) and handicap (Rho = 0.298, p < 0.05, Nagelkerke = 0.131). These results underline the need to develop the QoL of workers, so that they can subsequently improve their perception and care of oral health, also impacting on the performance and well-being of the individual. Thus, the results present implications at a global level, highlighting the importance of integrative models throughout different areas of the world.

### Recommendations

Develop continuing education programs: Create oral health-focused training strategies for hospital staff, highlighting the relevance of oral hygiene, prevention of dental diseases and their impact on QoL and professional performance. These initiatives should be incorporated into existing workplace wellness plans.

Improve access to dental services: Implement frequent dental services within the hospital, with flexible schedules that adjust to staff shifts. This would facilitate prevention, timely treatment and contribute to a better perception of oral health.

Monitoring oral health and QoL: Integrate standardized tools such as the OHIP-14 and HU-DBI into regular staff assessments. These measurements would allow for assessing the impact of implemented interventions and ensure a comprehensive approach to promoting overall well-being.

## Ethics and consent

This study was conducted under strict principles of scientific integrity and in compliance with the Code of Ethics for Research of the César Vallejo University (Universidad César Vallejo, 2024). ^28^ Likewise, international ethical standards were rigorously respected, including the provisions of the Council of International Organizations of Medical Sciences (CIOMS, 2016), the guidelines of the Belmont Report (National Commission for the Protection of Human Subjects of Biomedical and Behavioral Research, 1979) and the principles of the Declaration of Helsinki (World Medical Association, 2013). ^29– 31^ The study was approved by the Research Ethics Committee of the Master’s Program in Health Services Management at César Vallejo University, through the Resolution of the Vice-rectorate for Research No. 405-2024-VI-UCV, issued on January 30, 2025, with official confirmation on February 11, 2025. The ethics committee was made up of: president: Dr. Roberto Santiago Bellido García, secretary: Dr. Diaz Mujica Juana Yris, External member: Mgtr. Melecio Belisario Carlos Reyes, Alternate member: Dr. Patricia Luz Figueroa Garrido, Added member: Mtro. Omar Arturo Lizárraga Carrasco, Added member: Dr. Meneses La Riva Mónica Elisa. Likewise, all participants signed a written informed consent, in which the objectives, procedures, benefits, and risks of the study were detailed. Only those who fully understood and accepted these conditions participated. Furthermore, the university’s ethical policies were respected, ensuring the originality and rigor of the work, with a level of textual similarity of less than 17% in the documents generated.

## Data Availability

Zenodo: Dataset: Calidad de vida y percepción de salud bucal del personal hospitalario en Trujillo, Perú (2024)., Doi: https://doi.org/10.5281/zenodo.14847738. ^46^ This project contains the following underlying data:
•
DATABASE es.en.xls
The project contains the following underlying data: a CSV file titled “QoL_OHP_Responses.csv” that includes the anonymized responses to the CV and PSB questionnaires, and a PDF file titled “QoL_OHP_Metadata.pdf” that provides the metadata and description of the variables. Data are available under the terms of the Creative Commons Attribution 4.0 International license (CC-BY 4.0).
