# Relationship between the perception of oral health and the quality of life of hospital staff

**DOI:** 10.12688/f1000research.161146.2

**Published:** 2025-05-02

**Authors:** Miryam Lora Loza, Sheyla del Pilar Alvarado-Romero, Katia Ninozca Flores Ledesma, Nancy Cuenca Robles, David Rene Rodríguez Díaz

**Affiliations:** 1 Graduate School, César Vallejo University, Trujillo, La Libertad, 13001, Peru; 2 Graduate School of, Cesar Vallejo University, Lima, La Libertad, 13001, Peru; 3 School of Human Medicine, Private University of the North, Trujillo, Lima Norte, 13001, Peru

**Keywords:** Quality of life, Perception, Oral health, Correlation, Hospital, Functional limitation.

## Abstract

**Introduction:**

Hospital staff’s perception of oral health directly impacts their overall oral health-related well-being (OHRQoL) and their job performance. This study seeks to analyze the relationship between these two dimensions, providing information for designing strategies that promote a healthier work environment.

**Aim:**

To determine the relationship between oral health-related quality of life (OHRQoL) and oral health perceptions in the staff of a level II-1 hospital located in northern Peru.

**Methods:**

The study had a quantitative approach, with a cross-sectional, applied, and correlational design. Seventy-two participants participated. The validated OHIP-14 and HU-DBI questionnaires were used, with reliability coefficients of 0.847 and 0.804, respectively. Spearman’s correlation coefficient, appropriate for ordinal variables, was used for data analysis.

**Results:**

A statistically significant association was found between health-related quality of life and subjective perception of oral status (Rho = 0.391, p < 0.05), with an explained variance of 19.8% according to Nagelkerke’s pseudo R-squared. The most frequently associated quality of life dimensions were physical disability (Rho = 0.319; p < 0.05) and social disability (Rho = 0.242; p < 0.05). Excellent quality of life was the most prevalent (38.9%), while poor oral health was the most common (52.8%).

**Conclusion:**

The findings show a significant relationship between self-perceived oral health and oral health-related quality of life in this group of professionals. Promoting oral health strategies tailored to the hospital setting is recommended to improve workplace well-being.

## Introduction

Oral health is an essential component of overall well-being, as it directly impacts oral health-related quality of life (OHRQoL) through the ability to perform basic functions such as communicating, eating well, and maintaining satisfactory social relationships. ^1^ Oral diseases not only affect oral functions but also psychological, social, and economic outcomes, causing discomfort, pain, and loss of self-esteem, and impacting the subjective perception of oral well-being. ^2, 3^ The impact of oral health on OHRQoL underlines its direct contribution to achieving Sustainable Development Goal 3, which aims to ensure that all people achieve good health, with oral health being a fundamental component.

In this context, oral health is considered one of the basic global priorities for international organizations such as the International Dental Federation (FDI) and the World Health Organization (WHO), since its impact on the global quality of life linked to the area of oral health is undoubtedly significant. The World Health Organization conceptualizes oral health as a comprehensive condition that encompasses the physical, mental and social well-being of the individual, in relation to the functionality and state of the oral cavity, and not simply the absence of diseases or ailments in this area (WHO, 2022) and the FDI (2021) introduces an integrative perspective that relates oral well-being with sustainable public health policies. ^4, 5^

Oral diseases constitute a global public health problem affecting approximately 3.5 billion people worldwide, with a higher prevalence in developing and middle-income countries, where around 75% of cases are concentrated. ^4^ Untreated cases of caries are the most prevalent condition, reflecting deep inequalities in access to preventive services and basic treatments. ^6, 7^ In Latin America, periodontal diseases represent an epidemic that significantly impairs oral health-related quality of life (OHRQoL). In countries such as Peru, the magnitude of the problem is exacerbated by the low priority given to oral health within health agendas, evidenced by low public investment and high oral cancer rates: 2.60 per 100,000 women and 1.97 in men between 2000 and 2017. ^8^ This scenario is associated with limited resource allocation, the absence of effective preventive strategies, and the lack of early detection programs. ^9^ Faced with this challenge, the WHO and other international organizations have promoted a series of initiatives. Hence the definitive IED Vision 2030 Report and the Resolution (2021) on oral health, which emphasize that oral health should be part of Universal Health Coverage (UHC) systems and in line with the global agenda to combat non-communicable diseases (NCDs).

According to the 2022 World Oral Health Report, ^4^ it is essential to reorient public health policies to give a central role to the promotion and production of scientific knowledge in oral health, through national plans supported by the vision and international strategies proposed by the WHO in the aforementioned report. ^9^ The support and collaboration of institutions such as UNESCO or UNDP are essential to include oral health factors within well-being and sustainable human development policies. ^10, 11^ The oral health of healthcare personnel can be measured through oral health perception (OHP), having found that the practice of OHP can also influence not only oral well-being, but can also condition the quality of life based on oral health (OHRQoL), as well as the quality of oral health. Similarly, OHP can be conditioned by other contextual factors such as the significant limitation of accessibility to dental services or the workload. ^12– 14^ Negative OHP will affect self-esteem and interpersonal relationships, and will justify its assessment through OHRQoL in the health of healthcare personnel. ^8, 15^

The purpose of this study is to examine the potential relationship between quality of life and oral health-related aspects (OHRQoL) and the perception of oral well-being in the staff of a type II-1 hospital in northern Peru, taking into account their dimensions and interactions. This research provides relevant evidence for designing strategies that promote the oral well-being of healthcare staff and, consequently, guarantee comprehensive, patient-centered care based on quality criteria. ^16, 17^

## Methods

### Research type and design

This paper developed applied research aimed at solving a specific problem in order to propose solutions to the problems faced by hospital staff. ^18– 20^ The scope of the research was correlational, as it sought to identify the relationship between the variables and analyze the strength and direction of this relationship. ^21^ A non-experimental cross-sectional design was adopted, which allowed data to be collected at a single point in time without manipulating the variables, thus preserving the naturalness of the observation context. ^22^

### Population

The group was initially comprised of 80 members of the professional and technical team providing care at a Level II-1 healthcare facility in northern Peru. This group included 21 physicians, 10 obstetricians, 1 dentist, 1 pharmaceutical chemist, 17 nurses, 2 psychologists, 3 biologists, 5 microbiologists, 2 medical technologists, 13 nursing technicians, 4 pharmacy technicians, and 1 laboratory technician. After applying the inclusion criteria, which took into consideration designated or assigned personnel and CAS (Administrative Contracting of Services), with more than 6 months of seniority, those who agreed to complete each of the questions formulated in both questionnaires, adding their stamp and signature on the document referring to the informed consent, and the exclusion criteria considered workers with less than six months of seniority, those who were on vacation or sick leave, those who worked under an outsourcing modality, those who were carrying out SERUMS (Rural and Marginal Urban Health Service) and those who did not agree to participate in the study. A non-probabilistic intentional convenience sampling was used, obtaining a final sample made up of 72 participants, allowing for obtaining sufficient data to perform correlational analyses, considering the feasibility and accessibility of personnel during the study period. Additionally, to estimate the sample size, a statistical power analysis was performed with G*Power, establishing as parameters an expected correlation of medium magnitude (ρ = 0.30), a confidence level of 95% and a power of 80%. Under these parameters, the minimum size required was 67 participants, so the sample reached (n = 72) was adequate for the proposed correlational analysis. ^23^

### Variables

In this study, two main variables were evaluated: oral health-related quality of life (OHRQoL) and oral health perception (OHP). Oral health-related quality of life is conceived as an individual’s personal appreciation of how their oral condition influences different aspects of their daily life, both physically and emotionally and socially. ^24^ The OHIP-14 questionnaire (Oral Health Impact Profile), developed by Slade and Spencer, was used for its evaluation. This questionnaire allows estimating how oral conditions affect different areas of daily life. This instrument includes seven dimensions: functional limitation, psychological discomfort, physical pain, psychological disability, physical disability, social disability, and handicap. The overall score obtained on the instrument indicates the level of impact that oral health specifically has on the individual’s oral quality of life, without extending its scope to general quality of life.

Likewise, oral health perception refers to the subjective evaluation of oral status and its impact on QoL ^25^; it could be addressed through a modified version of the Hiroshima University – Dental Behavioral Inventory (HU-DBI), initially developed by Kawamura (1988) and adapted to the Peruvian context by Midolo (2023). ^26, 27^ This version was internally validated by Alvarado and Lora in 2024, specifically for health personnel. The instrument considers three dimensions: perception of knowledge, perception of behavior, and perception of attitude. This variable was used as an indicator of the subjective level of awareness, disposition, and practice of personnel in relation to their oral health.

### Data collection technique and instrument

The survey technique was used, since it facilitated the systematic collection of relevant data provided by the hospital’s professional and technical staff through standardized questionnaires, without altering the environment or the object of study. ^28^

Regarding the instruments, the Quality of Life (QoL) Questionnaire was used, originally designed by Slade and Spencer in 1994, adapted by Espinoza in 2017 ^29^ and subsequently validated and published by Espinoza et al. (2022). ^30^ This instrument consists of 14 items organized into seven dimensions: functional limitation, psychological discomfort, physical pain, psychological disability, physical disability, social disability and general disability, with two items per dimension. A five-point Likert-type scale was applied (0 = never, 4 = very frequently), and the results were categorized according to the global scoring system of the original instrument, without structural modifications.

For the purposes of analysis and interpretation, three categories were established: “excellent” (0–2 points), “regular” (3–9 points) and “poor” quality of life (10 points or more), following the methodological criteria proposed by Espinoza (2017) ^29^ and taken up by Espinoza et al. (2022), ^30^ who applied this classification to older adults in similar contexts, preserving the validity of the original instrument.

Likewise, the Oral Health Perception Questionnaire (HU-DBI), originally developed by Kawamura in 1988 and subsequently adapted by Midolo (2023) for healthcare personnel in Lima, Peru, was used. This modified version was internally validated by Alvarado and Lora in 2024, specifically for application in hospital settings. The instrument consists of 20 items distributed in three dimensions: perception of knowledge (8 items), perception of behavior (6 items), and perception of attitude (6 items). Responses were recorded on a dichotomous scale (Yes = 1, No = 0), and the results were categorized into three levels: poor (0–9 points), regular (10 points), and excellent (11–20 points).

Both instruments underwent an exhaustive validation process through expert judgment, in which five evaluators with experience in the field of health and scientific methodologies participated, with proven experience in psychometric evaluation of instruments. The judges analyzed each item considering criteria of internal consistency, conceptual clarity, thematic relevance and informative sufficiency, achieving a level of total agreement reflected in an Aiken’s V coefficient equal to 1.00, which demonstrates high content validity. The reliability of the instruments was established by executing a pilot test prior to their final implementation. The analysis yielded a Cronbach’s alpha coefficient of 0.847 for the OHIP-14 questionnaire, and 0.804 for the HU-DBI instrument, results that demonstrate solid and acceptable internal consistency according to current methodological standards.

To ensure methodological traceability and facilitate future replications, the instruments used (OHIP-14 and the modified version of the HU-DBI) have been fully incorporated as complementary material, publicly available in the Zenodo repository: https://doi.org/10.5281/zenodo.15236712. This file includes the questionnaires used, the validation matrix prepared by five expert judges and the reliability indicators obtained during the pilot study, thus supporting the validity and applicability of the instruments in similar methodological contexts. ^31^

### Procedure

The procedure began with the submission of a formal cover letter issued by the Graduate School of César Vallejo University to the administrative department of La Libertad Hospital, Category II-1, requesting authorization to administer the research instruments. Once approval was received from the institution, personal attendance at the hospital was coordinated by scheduling specific dates and times for the application. During these information sessions, participants were provided with a clear and detailed explanation of the study’s objectives and purposes, ensuring the confidentiality of the information collected and the anonymity of their identities, in accordance with the ethical principles of scientific research. Each participant signed the corresponding informed consent form prior to administering the proposed questionnaires. The instruments were administered in separate spaces within their work areas and lasted approximately 8 minutes per person, taking care to minimize potential distractions or contextual biases.

### Information analysis

Statistical analysis of the data was performed using IBM SPSS Statistics, version 25 ( https://www.ibm.com/products/spss-statistics ). Initially, the Kolmogorov-Smirnov test was applied to determine the normal distribution of the variables; the results indicated a non-normal distribution (p < 0.05). Consequently, non-parametric tests were used. To examine the relationship between oral health-related quality of life (OHRQoL) and oral health perception (OHP), Spearman’s Rho correlation coefficient was used, as it is the appropriate statistic for the analysis of ordinal variables. An ordinal logistic regression model was also used to assess the relationship between the variables, including Nagelkerke’s Pseudo R-squared as the measure of fit. Confidence intervals (CIs) were also included, as they contribute to a better approximation of the results obtained in the analysis. As an alternative, the use of the statistical software R (The R Project for Statistical Computing) was also considered. The analysis identified a statistically significant association between the variables studied, providing empirical evidence of the interdependence between oral health-related quality of life and perceptions of oral health among hospital healthcare personnel. ^32– 35^

### Ethical implications

This study was conducted in strict compliance with the principles of scientific integrity and respect for the rights of participants, as established in institutional codes and current ethical regulations. International guidelines for research involving human subjects were considered, such as the guidelines of the Council for International Organizations of Medical Sciences (CIOMS, 2016), ^36^ the recommendations of the Belmont Report (1979), ^37^ and the principles established in the Declaration of Helsinki (World Medical Association, 2013). ^38^ The proposal was approved by a duly constituted institutional ethics committee, which evaluated and authorized its execution through a formal resolution issued in the first quarter of 2025. All phases of the study respected the criteria of confidentiality, informed consent, and voluntary participation of participants. Furthermore, all participants voluntarily agreed to sign written informed consent, which detailed the objectives, procedures, benefits, and risks of the study. Only those who fully understood and accepted these conditions participated. Furthermore, the university’s ethical policies were respected, as described in the institutional Research Ethics Code, ^39^ which ensured the originality, transparency, and methodological rigor of the research.

## Results

Table 1 It is observed that 38.90% of staff with excellent QoL reported a low OHP of 52.80%, while the 34.70% with poor QoL presented a more balanced distribution across OHP categories. Likewise, a low (r = 0.391), but significant (p = 0.001), positive correlation was evident between QoL and OHP. Likewise, ordinal logistic regression analysis, with a Nagelkerke pseudo R-squared of 0.198 (p = 0.001), confirmed a significant influence of QoL on OHP, accounting for 19.80%. To ensure inferential accuracy, confidence intervals (CI) and Nagelkerke pseudo R-squared values were included in all regression tests, as detailed in Tables 1 and 4.

**Table 1.** Relationship between Quality of Life and Perception of Oral Health of Health Personnel at a Level II-1 Hospital in Northern Peru, 2024.

Quality of life (CV)	Oral Health Perception (OHP)						Total	
	Low		Regular		Excellent			
	N	%	N	%	N	%	N	%
**Excellent**	21	29,20	6	8.30	1	1.40	28	38.90
**Regular**	8	11,10	1	1.40	10	13.90	19	26.40
**Bad**	9	12.50	6	8.30	10	13.90	25	34.70
**Total**	38	52.80	13	18,10	21	29,20	72	100,00

*Note:* Correlation between quality of life and oral health, data processed with SPSS, own elaboration.

Table 2 The findings show that quality of life is predominantly grouped into three distinct categories or levels: excellent (38.90%), poor (34.70%), and fair (26.40%). Furthermore, when analyzing the dimensions of quality of life, it is observed that all reached high percentages at the excellent level. Functional limitation was the most common, with 44.40%, followed by physical pain, which reached 52.80%. Psychological distress also reached 54.20%, while physical and psychological disability registered 69.40%. Social disability reached 76.40%, and finally, disability had the highest percentage, at 83.30%.

**Table 2.** Quality of Life Level and its dimensions of the health personnel of a Level II-1 Hospital in Northern Peru, 2024.

Rho Spearman	Next	Pseudo R Nagelkerke	Next
0.391	0.001	0.198	0.001

CV			CV Dimensions													
			Limitation functional		Pain physical		Discomfort psychological		Physical disability		Inability psychological		Social incapacity		Handicap	
Levels	N	%	N	%	N	%	N	%	N	%	N	%	N	%	N	%
**Excellent**	28	38.90	32	44.40	38	52.80	39	54.20	50	69.40	50	69.40	55	76.40	60	83.30
**Regular**	19	26.40	28	38.90	23	31.90	22	30.60	18	25.00	18	25.00	14	19.40	10	13.90
**Bad**	25	34.70	12	16.70	11	15.30	11	15.30	4	5.60	4	5.60	3	4.20	2	2.80
**Total**	72	100.00	72	100.00	72	100.00	72	100.00	72	100.00	72	100.00	72	100.00	72	100.00

*Note:* Statistical analysis of oral health perception, processed questionnaires, own elaboration.

Table 3 It indicates that the Oral Health Perception (OHP) is distributed mainly into three levels: low (52.80%), excellent (29.20%), and average (18.00%). When examining the different dimensions, it was identified that the Knowledge Perception registered 100% at a low level. On the other hand, the Attitude Perception showed 44.4% at a low level, while the Behavior Perception was located at 62.5% at a fair level.

**Table 3.** Level of oral perception and its dimensions in health personnel from a level II-1 hospital in northern Peru, 2024.

PSB			PSB Dimensions					
			Perception of knowledge		Attitude perception		Perception of behavior	
Levels	N	%	N	%	N	%	N	%
**Low**	38	52.80	72	100.00	32	44.40	15	20.80
**Regular**	13	18.00	0	0.00	31	43.10	45	62.50
**Excellent**	21	29.20	0	0.00	9	12.50	12	16.70
**Total**	72	100.00	72	100.00	72	100.00	72	100.00

*Note:* Statistical analysis of oral health perception, processed questionnaires, own elaboration.

Table 4 This paper presents a detailed analysis showing the relationships between the Quality of Life (QoL) dimensions and Oral Health Perception (OHP), highlighting important differences in the magnitude of these relationships. In particular, the psychological distress dimension showed the most significant connection with OHP, supported by a moderate correlation coefficient (r = 0.421) and a solid level of statistical significance (p = 0.000). Furthermore, Nagelkerke’s Pseudo R-squared obtained a value of 0.111 (p = 0.027), which explains that this dimension predicts 11.1% of the variability of OHP. Physical disability, on the other hand, showed a significant correlation with OHP, although with a smaller effect size than the previous dimension. The correlation coefficient was 0.319 (p = 0.006), while the Pseudo R-squared value was 0.167 (p = 0.004), indicating a 16.7% effect on OHP. In contrast, the dimension associated with functional limitations indicated a much weaker relationship with OHP, with a correlation coefficient of 0.096 (p = 0.424) and a Pseudo R-squared of 0.014 (p = 0.649), suggesting a practically null effect on the target variable.

**Table 4.** Relationship between the dimensions of the Quality of life with the perception of oral health of the staff of a level II-1 hospital in La Libertad, 2024.

Functional limitation	Oral Health Perception								Inferential analysis			
	Low		Regular		Excellent		Total		Rho Spearman	Next	Pseudo R Nagelkerke	Next
	N	%	N	%	N	%	N	%				
Excellent	18	25.00	7	9.70	7	9.70	32	44.40	0.096	0.424	0.014	0.649
Regular	15	20.80	3	4.20	10	13.90	28	38.90				
Bad	5	6.90	3	4.20	4	5.60	12	16.70				
**Total**	38	52.80	13	18.10	21	29.20	72	100.00				

Pain physical	PSB						Total					
	Low		Regular		Excellent							
	N	%	N	%	N	%	N	%				
Excellent	24	33.30	7	9.70	7	9.70	38	52.80	0.266	0.024	0.093	0.049
Regular	12	16.70	2	2.80	9	12.50	23	31.90				
Bad	2	2.80	4	5.60	5	6.90	11	15.30				
**Total**	38	52.80	13	18.10	21	29.20	72	100.00				

Discomfort psychological	PSB						Total					
	Low		Regular		Excellent							
	N	%	N	%	N	%	N	%				
Excellent	26	36.10	6	8.30	7	9.70	39	54.20	0.421	0.000	0.111	0.027
Regular	8	11.10	5	6.90	9	12.50	22	30.60				
Bad	4	5.60	2	2.80	5	6.90	11	15.30				
**Total**	38	52.80	13	18.10	21	29.20	72	100.00				

Physical disability	PSB						Total					
	Low		Regular		Excellent							
	N	%	N	%	N	%	N	%				
Excellent	30	41.70	8	11.10	12	16.70	50	69.40	0.319	0.006	0.167	0.004
Regular	8	11.10	5	6.90	5	6.90	18	25.00				
Bad	0	0.00	0	0.00	4	5.60	4	5.60				
**Total**	38	52.80	13	18.10	21	29.20	72	100.00				

Inability psychological	PSB						Total					
	Low		Regular		Excellent							
	N	%	N	%	N	%	N	%				
Excellent	30	41.70	8	11.10	12	16.70	50	69.40	0.232	0.050	0.167	0.004
Regular	8	11.10	5	6.90	5	6.90	18	25.00				
Bad	0	0.00	0	0.00	4	5.60	4	5.60				
**Total**	38	52.80	13	18.10	21	29.20	72	100.00				

Inability social	PSB						Total					
	Low		Regular		Excellent							
	N	%	N	%	N	%	N	%				
Excellent	31	43.10	11	15.30	13	18.10	55	76.40	0.242	0.040	0.124	0.017
Regular	7	9.70	2	2.80	5	6.90	14	19.40				
Bad	0	0.00	0	0.00	3	4.20	3	4.20				
**Total**	38	52.80	13	18.10	21	29.20	72	100.00				

Handicap	PSB						Total					
	Low		Regular		Excellent							
	N	%	N	%	N	%	N	%				
Excellent	36	50.00	9	12.50	15	20.80	60	83.30	0.298	0.011	0.131	0.013
Regular	2	2.80	4	5.60	4	5.60	10	13.90				
Bad	0	0.00	0	0.00	2	2.80	2	2.80				
**Total**	38	52.80	13	18.10	21	29.20	72	100.00				

*Note:* Association between dimensions of quality of life and oral health, analysis in SPSS, own elaboration.

Overall, these findings confirm the existence of a positive relationship between quality-of-life dimensions and oral health problems. However, it is evident that the dimensions of psychological distress and physical disability have a greater influence on oral health, underscoring the need to implement specific interventions targeting these critical areas.

## Discussion

Quality of life (QoL) and oral health perception (OHP) are essential elements that directly influence the general well-being of hospital staff. In this study, the relationship between these variables was established in a total of 72 workers from a hospital located in northern Peru during the year 2024, with the purpose of identifying and establishing areas of opportunity that allow the execution of strategies that address the specific needs of the group. First, these results suggest in Table 1 show that there is a low-level positive correlation (r = 0.391), but statistically significant (p < 0.05). Furthermore, it was shown that QoL influences OHP by 19.8%. It should be noted that the highest frequency of cases corresponds to healthcare personnel who reported an optimal QoL, despite experiencing low levels of OHP (29.2%). These findings are similar to those obtained by Miranda and Alcocer in 2021, who found that the sociodemographic characteristics of older adults influence the perception of quality of life and its link to oral health, without generating significant negative impacts. In their study, they identified that a high percentage of participants maintained excellent (45.4%) or moderate (34.6%) levels of quality of life. Therefore, when healthcare professionals experience a favorable quality of life, problems associated with oral health tend to go unnoticed, as they do not significantly interfere with their daily activities or work performance. ^2, 12^

Table 2 shows that quality of life (QoL) is distributed into three main categories: excellent at 38.9%, poor at 34.7%, and fair at 26.4%. Furthermore, when analyzing the seven dimensions of QoL, it is observed that all reached high percentages at the excellent level. First, Functional Limitation obtained 44.40%, followed by Physical Pain at 52.80%. Likewise, Psychological Distress reached 54.20%, while Physical and Psychological Disability registered 69.40%. On the other hand, Social Disability reached 76.40%, and finally, Handicap reached the highest percentage at 83.30%. The data are similar to those obtained by Espinoza et al. (2022), who conducted a study on the quality of life and its relationship with the oral health of the members of a geriatric center in Lima. Their findings revealed that quality of life was excellent in 66.8% of cases and that oral health did not negatively affect this perception. ^30^ These results may be explained by the importance healthcare professionals attach to oral health care, which is reflected in a positive perception of their quality of life. Theory suggests that good oral health is closely related to a better quality of life, as it reduces pain and discomfort, and improves functional and social capacity. ^40^

Table 3 presents the most relevant data on OHP and its dimensions among healthcare personnel at a hospital in northern Peru. OHP was mainly distributed into three levels: low (52.8%), excellent (29.2%), and regular (18.0%). Likewise, the analysis of the specific dimensions showed that the low level predominated with 100% in the Perception of Knowledge. Meanwhile, the Perception of Attitude registered 44.4%, also at a low level. Finally, the Perception of Behavior was mostly located at the regular level with 62.5%. These findings differ considerably from previous studies, such as that of López (2021), who indicated that the level of knowledge about OHP among healthcare workers at EsSalud Hospital II during the Covid-19 pandemic was high, reaching 81.5%, demonstrating a good command of this dimension in this context. ^41^ Therefore, the limited perception of knowledge could be linked to the lack of ongoing oral health (OH) training programs for healthcare personnel. This is consistent with previous studies highlighting that training and education are essential elements for strengthening knowledge and promoting appropriate occupational health practices. Furthermore, attitude and behavior reflect not only the level of knowledge but also cultural beliefs and traditions related to occupational health, which can influence the adoption of preventive habits and the search for appropriate treatments. ^42, 43^

Table 4 presents the results on the relationship between functional limitation and perceived oral health in workers. It was observed that the highest percentage corresponds to those with an excellent quality of life (QoL) but with low OLP, representing 25.0%. However, the statistical analysis showed that there is no significant correlation (r = 0.096) or a relevant influence between this dimension and the variable (p > 0.05). When comparing these results with previous research, similar findings are identified. As García-Cortés et al. (2020) reported that OLP did not show a statistically significant relationship with QoL in a study conducted with healthcare professionals in Spain. ^44^ Similarly, López-Jiménez et al. (2019) reported that, although functional limitations existed, these did not significantly affect OLP in a sample of nurses in Mexico. ^45^ On the other hand, some studies have found a significant relationship between these variables in different contexts. For example, Martínez-Rodríguez et al. (2018) found that functional limitations did affect OHP in a sample of older adults in Chile. ^46^

Regarding the influence of physical pain and the perception of oral health in workers, a low-intensity positive correlation was identified (r = 0.266), with a limited influence of 9.3% of this dimension on the OHP. Likewise, the highest percentage was found in workers with excellent QoL, but with low OHP (33.30%). These data are similar to the research by Campos et al. (2014) who conducted an analysis of how job performance is affected by alterations in the oral cavity and that, in relation to physical pain, this had a negative influence of 82.90%. ^47^ This indicates that the absence of physical pain attributed to alterations in the oral cavity may lead to the OHP going unnoticed. ^48^

Similarly, when evaluating the relationship between psychological distress and OHP in workers, a moderate correlation was observed (r = 0.421) with a high level of statistical significance (p < 0.01) and a relevant influence of 11.1% of this dimension on OHP. The highest percentage corresponds to workers with an excellent CV, but with a low OHP (10.41). These results are similar to those found by Espinoza (2017), who identified a negative impact of 61.4% in relation to psychological distress. ^30^ This suggests that the absence of psychological distress related to the oral cavity may lead to OHP not being considered. ^48^

Regarding the link between physical disability and OHP in workers, a significant, low-magnitude correlation was observed (r = 0.319, p < 0.05), with a notable influence of 16.7% of this dimension on Oral Health Perception (OHP). These findings contrast with the results of Bellamy and Moreno (2014), who reported that physical disability was one of the most affected dimensions in patients with removable prostheses and tooth loss, reaching 32.5%. ^49^ Thus, when there is no physical disability, OHP goes unnoticed; and, conversely, if this dimension is present, oral health is prioritized. ^50^

Likewise, in relation to psychological disability and the OHP of workers, a low correlation was identified (r=0.232) with a significant influence of 16.7% on the OHP. In addition, the highest percentage corresponded to those workers with an excellent quality of life and low OHP (41.7%). Similarly, when the disability psychological ^30^ is at a regular level, the OHP is at regular or excellent levels, with 6.9% in both cases. These findings are consistent with those reported by Espinoza (2017), who showed a negative influence of oral health on QoL, reflecting a 31.5% impact in relation to psychological disability. In this sense, when workers do not have psychological disability, OHP is not usually a priority. On the other hand, when there is psychological disability, it favors the adoption of actions that directly influence oral health. ^50^

Regarding the link between social disability and oral health (OHP) among hospital care workers, a significant (p<0.05) correlation was found, a low (r=0.242) correlation, and a significant influence of 12.4% of the dimension on oral health (OHP). It should be noted that the highest percentage was achieved by workers with excellent QoL but low OHP (43.10%). Likewise, if the social disability is poor, OHP is exclusively outstanding (4.20%). These data can be contrasted with those evidenced by Espinoza (2017), who found an inverse relationship between oral health and quality of life, highlighting a 23.40% share in the social disability dimension. ^30^ Therefore, when workers do not have social disabilities, the OHP is not considered important; otherwise, if workers have social disabilities, this favors practical behaviors that influence SO. ^51^

Finally, regarding the link between disability and workers’ CPS, a significant correlation of low magnitude was identified (r = 0.298, p < 0.05), with an influence of 13.1% of this dimension on the Perceived Oral Health (CPS). Furthermore, the highest percentage corresponded to workers with excellent quality of life and low CPS, reaching 50.0%. Similarly, when the disability is present at moderate levels, the CPS was distributed between excellent and regular, with 5.6% in each category. These data are similar to those reported by Espinoza et al. (2022), who showed a negative link between oral health and QoL, reflecting an impact of 17.0% in relation to disability. ^30^ Thus, when workers do not show signs of disability, CPS often takes a backseat. In contrast, when some type of disability is present, this condition can motivate the implementation of differentiated behaviors and practices that directly impact the state of oral health. ^51^

### Limitations of the study

The discussion of this study was based on the limitations of the design and sample size. Because it was conducted in a single Level II-1 health center in northern Peru, the results obtained cannot be extrapolated to other populations or different geographic contexts due to the specific characteristics of the sample and the study setting. Similarly, the sample size of 72 participants limits the possibility of generalizing any inferences to healthcare personnel. Therefore, the findings should be considered with caution and in the context of the research.

It is important to emphasize that the concept evaluated focuses specifically on oral health-related quality of life (OHRQoL). This is demonstrated by the use of validated instruments used to assess oral status in relation to quality of life, which confirms that the assessment is carried out with the necessary care in the oral health field, which is the responsibility of hospital staff.

Although this research has provided relevant information on the relationship between quality of life (QoL) and perceived oral health (POH) among hospital staff, it is important to consider a few limitations. First, the transversality of the design of exploratory studies such as this one does not allow for establishing relationships. However, the results provide a good approximation for future longitudinal investigations that will allow us to verify how these same relationships evolve over time. Furthermore, although the sample is representative of a specific hospital, replicating the research in different centers in Spain would help improve the generalizability of the results and identify similar patterns in the research across different contexts.

Finally, although self-assessment using questionnaires is valid and reliable, it undermines the subjectivity of the study subject. However, the use of standardized and recognized instruments ensures comparability and validity of the data.

### Implications of the study

The findings underscore the importance of oral health as one of the core elements of the overall well-being of hospital staff. The existence of a significant correlation between quality of life and psychological well-being, along with the involvement of certain dimensions such as physical disability, psychosocial disability, etc., highlights the need for interventions that address oral health as well as psychosocial factors, determinants of personal well-being and, therefore, of the quality of service offered to patients. Finally, although the results can be used to guide the development of strategies in other similar contexts, their implications should be considered with caution and within the framework of the local context under study. This encourages the adoption of multidimensional and collaborative approaches in hospital settings that would empower them to promote sustainable development and well-being.

## Conclusion

The study shows a statistically significant correlation between oral health-related quality of life (OHRQoL) and perceptions of oral health among healthcare professionals at a Level II-1 hospital located in northern Peru. The Spearman correlation coefficient was 0.391 and the Nagelkerke pseudo R-squared was 0.198, both with a statistical significance of 0.001. Higher oral health-related quality of life (OHRQoL) is significantly related to more favorable perceptions of oral health. Furthermore, relevant relationships were identified in the dimensions of physical disability (Rho = 0.319, p < 0.05; Nagelkerke = 0.167), psychological disability (Rho = 0.232, p = 0.05; Nagelkerke = 0.167), social disability (Rho = 0.242, p < 0.05; Nagelkerke = 0.124), and general disability (Rho = 0.298, p < 0.05; Nagelkerke = 0.131). These conclusions could corroborate that reinforcing the factors that positively impact teachers’ oral health-related quality of life could be related to a more favorable perception of their oral health. In this sense, the results provide contextual evidence that could be a reference for other similar contexts, without losing sight of the fact that sociocultural aspects are different in each context.

### Recommendations

Develop continuing education programs: Create training strategies focused on oral health for hospital staff, highlighting the importance of oral hygiene, the prevention of dental diseases, and their impact on quality of life and professional performance. It is essential to integrate these initiatives into existing workplace wellness programs.

Improve access to dental services: Implement frequent dental services within the hospital, with flexible schedules that fit staff shifts. This would facilitate prevention, timely treatment, and contribute to better perceptions of oral health.

Oral health and quality of life monitoring: Integrate standardized tools such as the OHIP-14 and HU-DBI into regular staff assessments. These measures would allow for evaluating the impact of implemented interventions and ensure a comprehensive approach to promoting overall well-being.

## Data Availability

Regarding the availability of the data used, these are publicly accessible on the Zenodo platform, under the terms of the Creative Commons Attribution 4.0 International (CC-BY 4.0) license. The main database can be consulted at: https://doi.org/10.5281/zenodo.14847738 This file in Excel format (.xls) contains the anonymized responses to the Quality of Life (QoL) and Oral Health Perception (OHP) questionnaires applied to hospital staff. ^52^ The complementary methodological appendix, which includes the complete instruments (OHIP-14 and HU-DBI in Spanish), as well as the expert judgment validation matrix and the reliability levels obtained, is available at: https://doi.org/10.5281/zenodo.15236712. ^31^ These files ensure the transparency and reproducibility of the data collection process.
