# Relationship between the perception of oral health and the quality of life of hospital staff

**DOI:** 10.12688/f1000research.161146.3

**Published:** 2025-06-16

**Authors:** Miryam Lora Loza, Sheyla del Pilar Alvarado-Romero, Katia Ninozca Flores Ledesma, Nancy Cuenca Robles, David Rene Rodríguez Díaz

**Affiliations:** 1 Graduate School, César Vallejo University, Trujillo, La Libertad, 13001, Peru; 2 Graduate School of, Cesar Vallejo University, Lima, La Libertad, 13001, Peru; 3 School of Human Medicine, Private University of the North, Trujillo, Lima Norte, 13001, Peru

**Keywords:** Quality of life, Perception, Oral health, Correlation, Hospital, Functional limitation.

## Abstract

**Introduction:**

Hospital staff’s perception of oral health directly impacts their overall oral health-related well-being (OHRQoL) and their job performance. This study seeks to analyze the relationship between these two dimensions, providing information for designing strategies that promote a healthier work environment.

**Aim:**

To determine the relationship between oral health-related quality of life (OHRQoL) and oral health perceptions in the staff of a level II-1 hospital located in northern Peru.

**Methods:**

The study had a quantitative approach, with a cross-sectional, applied, and correlational design. Seventy-two participants participated. The validated OHIP-14 and HU-DBI questionnaires were used, with reliability coefficients of 0.847 and 0.804, respectively. Spearman’s correlation coefficient, appropriate for ordinal variables, was used for data analysis.

**Results:**

A statistically significant association was found between health-related quality of life and subjective perception of oral status (Rho = 0.391, p < 0.05), with an explained variance of 19.8% according to Nagelkerke’s pseudo R-squared. The most frequently associated quality of life dimensions were physical disability (Rho = 0.319; p < 0.05) and social disability (Rho = 0.242; p < 0.05). Excellent quality of life was the most prevalent (38.9%), while poor oral health was the most common (52.8%).

**Conclusion:**

The findings show a significant relationship between self-perceived oral health and oral health-related quality of life in this group of professionals. Promoting oral health strategies tailored to the hospital setting is recommended to improve workplace well-being.

## Introduction

Oral health is an essential component of overall well-being, as it directly is associated with oral health-related quality of life (OHRQoL) through the ability to perform basic functions such as communicating, eating well, and maintaining satisfactory social relationships. ^1^ Oral diseases not only affect oral functions but also psychological, social, and economic outcomes, causing discomfort, pain, and loss of self-esteem, and associationing the subjective perception of oral well-being. ^2^ ^,^ ^3^ The association of oral health on OHRQoL underlines its direct contribution to achieving Sustainable Development Goal 3, which aims to ensure that all people achieve good health, with oral health being a fundamental component.

In this context, oral health is considered one of the basic global priorities for international organizations such as the International Dental Federation (FDI) and the World Health Organization (WHO), since its relationship with the global oral health-related quality of life (OHRQoL) linked to the area of oral health is undoubtedly significant. The World Health Organization conceptualizes oral health as a comprehensive condition that encompasses the physical, mental and social well-being of the individual, in relation to the functionality and state of the oral cavity, and not simply the absence of diseases or ailments in this area (WHO, 2022), and the FDI (2021) introduces an integrative perspective that relates oral well-being with sustainable public health policies. ^4^ ^,^ ^5^

Oral diseases constitute a global public health problem affecting approximately 3.5 billion people worldwide, with a higher prevalence in developing and middle-income countries, where around 75% of cases are concentrated. ^4^ Untreated cases of caries are the most prevalent condition, reflecting deep inequalities in access to preventive services and basic treatments. ^6^ ^,^ ^7^ In Latin America, periodontal diseases represent an epidemic that significantly impairs oral health-related quality of life (OHRQoL). In countries such as Peru, the magnitude of the problem is exacerbated by the low priority given to oral health within health agendas, evidenced by low public investment and high oral cancer rates: 2.60 per 100,000 women and 1.97 in men between 2000 and 2017. ^8^ This scenario is associated with limited resource allocation, the absence of effective preventive strategies, and the lack of early detection programs. ^9^ Faced with this challenge, the WHO and other international organizations have promoted a series of initiatives. Hence the definitive IED Vision 2030 Report and the Resolution (2021) on oral health, which emphasize that oral health should be part of Universal Health Coverage (UHC) systems and in line with the global agenda to combat non-communicable diseases (NCDs).

According to the 2022 World Oral Health Report, ^4^ it is essential to reorient public health policies to give a central role to the promotion and production of scientific knowledge in oral health, through national plans supported by the vision and international strategies proposed by the WHO in the aforementioned report. ^9^ The support and collaboration of institutions such as UNESCO or UNDP are essential to include oral health factors within well-being and sustainable human development policies. ^10^ ^,^ ^11^ The oral health of healthcare personnel can be measured through oral health perception (OHP), having found that the practice of OHP can also be associated with not only oral well-being, but can also condition the oral health-related quality of life (OHRQoL) as well as the quality of oral health. Similarly, OHP can be conditioned by other contextual factors such as the significant limitation of accessibility to dental services or the workload. ^12^ ^–^ ^14^ Negative OHP will affect self-esteem and interpersonal relationships, and will justify its assessment through OHRQoL in the health of healthcare personnel. ^8^ ^,^ ^15^

The purpose of this study is to examine the potential relationship between oral health-related quality of life (OHRQoL) and the perception of oral well-being in the staff of a type II-1 hospital in northern Peru, taking into account their dimensions and interactions. This research provides relevant evidence for designing strategies that promote the oral well-being of healthcare staff and, consequently, guarantee comprehensive, patient-centered care based on quality criteria. ^16^ ^,^ ^17^

## Methods

### Research type and design

This paper presents applied research aimed at solving a specific problem in order to propose solutions to the challenges faced by hospital staff. ^18^ ^–^ ^20^ The scope of the research was correlational, as it sought to identify the relationship between the variables and analyze the strength and direction of this relationship. ^21^ A non-experimental cross-sectional design was adopted, which allowed data to be collected at a single point in time without manipulating the variables, thus preserving the natural context of observation. ^22^

### Population

The group was initially comprised of 80 members of the professional and technical team providing care at a Level II-1 healthcare facility in northern Peru. This group included 21 physicians, 10 obstetricians, 1 dentist, 1 pharmaceutical chemist, 17 nurses, 2 psychologists, 3 biologists, 5 microbiologists, 2 medical technologists, 13 nursing technicians, 4 pharmacy technicians, and 1 laboratory technician. After applying the inclusion criteria, which took into consideration designated or assigned personnel and CAS (Administrative Contracting of Services), with more than 6 months of seniority, those who agreed to complete each of the questions formulated in both questionnaires, signed and stamped the informed consent form. The **exclusion criteria** considered workers with less than six months of seniority, those who were on vacation or sick leave, those who worked under an outsourcing modality, those who were carrying out SERUMS (Rural and Marginal Urban Health Service) and those who did not agree to participate in the study. A non-probabilistic intentional convenience sampling was used, obtaining a final sample made up of 72 participants, which allowed for the collection of sufficient data to perform correlational analyses, considering the feasibility and accessibility of personnel during the study period. Additionally, to estimate the sample size, a statistical power analysis was performed with G*Power, establishing as parameters an expected correlation of medium magnitude (ρ = 0.30), a confidence level of 95% and a power of 80%. Under these parameters, the minimum size required was 67 participants, so the sample reached (n = 72) was adequate for the proposed correlational analysis. ^23^

### Variables

In this study, two main variables were evaluated: oral health-related quality of life (OHRQoL) and oral health perception (OHP). Oral health-related quality of life (OHRQoL) is conceived as an individual’s personal appreciation of how their oral condition is associated with different aspects of their daily life, both physically and emotionally and socially. ^24^ The OHIP-14 questionnaire (Oral Health Impact Profile), developed by Slade and Spencer, was used for its evaluation. This questionnaire allows estimating how oral conditions affect different areas of daily life. This instrument includes seven dimensions: functional limitation, psychological discomfort, physical pain, psychological disability, physical disability, social disability, and handicap. The overall score obtained on the instrument indicates the level of association that oral health specifically has on the individual’s oral health-related quality of life (OHRQoL), without extending its scope to general quality of life (QoL).

Likewise, oral health perception refers to the subjective evaluation of oral status and its **relationship with** QoL ^25^; it could be addressed through a modified version of the Hiroshima University – Dental Behavioral Inventory (HU-DBI), initially developed by Kawamura (1988) and adapted to the Peruvian context by Midolo (2023). ^26^ ^,^ ^27^ This version was internally validated by Alvarado and Lora in 2024, specifically for health personnel. The instrument considers three dimensions: perception of knowledge, perception of behavior, and perception of attitude. This variable was used as an indicator of the subjective level of awareness, disposition, and practice of personnel in relation to their oral health.

### Data collection technique and instrument

The survey technique was used, since it facilitated the systematic collection of relevant data provided by the hospital’s professional and technical staff through standardized questionnaires, without altering the environment or the object of study. ^28^

Regarding the instruments, the oral health-related quality of life (OHRQoL) Questionnaire was used, originally designed by Slade and Spencer in 1994, adapted by Espinoza in 2017 ^29^ and subsequently validated and published by Espinoza et al. (2022). ^30^ This instrument consists of 14 items organized into seven dimensions: functional limitation, psychological discomfort, physical pain, psychological disability, physical disability, social disability, and general disability, with two items per dimension. A five-point Likert-type scale was applied (0 = never, 4 = very frequently), and the results were categorized according to the global scoring system of the original instrument, without structural modifications.

For the purposes of analysis and interpretation, three categories were established: “excellent” (0–2 points), “regular” (3–9 points), and “poor” oral health-related quality of life (OHRQoL) (10 points or more), following the methodological criteria proposed by Espinoza (2017) ^29^ and taken up by Espinoza et al. (2022), ^30^ who applied this classification to older adults in similar contexts, preserving the validity of the original instrument.

Likewise, the Oral Health Perception Questionnaire (modified HU-DBI),originally developed by Kawamura in 1988 and subsequently adapted by Midolo (2023) for healthcare personnel in Lima, Peru, was used. This modified version was internally validated by Alvarado and Lora in 2024, specifically for application in hospital settings. The instrument consists of 20 items distributed in three dimensions: perception of knowledge (8 items), perception of behavior (6 items), and perception of attitude (6 items). Responses were recorded on a dichotomous scale (Yes = 1, No = 0), and results were categorized into three levels: poor (0–9 points), regular (10 points), and excellent (11–20 points).

Both instruments underwent an exhaustive validation process through expert judgment, in which five evaluators with experience in the field of health and scientific methodologies participated, with proven experience in the psychometric evaluation of measurement tools. The judges analyzed each item considering criteria of internal consistency, conceptual clarity, thematic relevance, and informative sufficiency, achieving a level of total agreement reflected in an Aiken’s V coefficient equal to 1.00, which demonstrates high content validity. The reliability of the instruments was established by conducting a pilot test prior to their final implementation. The analysis yielded a Cronbach’s alpha coefficient of 0.847 for the OHIP-14 questionnaire, and 0.804 for the HU-DBI instrument, results that demonstrate solid and acceptable internal consistency according to current methodological standards.

To ensure methodological traceability and facilitate future replications, the instruments used (OHIP-14 and the modified HU-DBI) have been fully incorporated as complementary material, publicly available in the Zenodo repository: https://doi.org/10.5281/zenodo.15236712. This file includes the questionnaires used, the validation matrix reviewed by five expert judges and the reliability indicators obtained during the pilot study, thus supporting the validity and applicability of the instruments in similar methodological contexts. ^31^

### Procedure

The procedure began with the submission of a formal cover letter issued by the Graduate School of César Vallejo University to the administrative department of La Libertad Hospital, Category II-1, requesting authorization to administer the research instruments. Once approval was received from the institution, in-person attendance at the hospital was coordinated by scheduling specific dates and times for the application. During these information sessions, participants were provided with a clear and detailed explanation of the study’s objectives and purposes, ensuring the confidentiality of the information collected and the anonymity of their identities, in accordance with the ethical principles of scientific research. Each participant signed the corresponding informed consent form prior to administration of the proposed questionnaires. The instruments were administered in separate spaces within their work areas and required approximately 8 minutes per person, taking care to minimize potential distractions or contextual biases.

### Information analysis

Statistical analysis of the data was performed using IBM SPSS Statistics, version 25 ( https://www.ibm.com/products/spss-statistics ). Initially, the Kolmogorov-Smirnov test was applied to determine the normal distribution of the variables; the results indicated a non-normal distribution ( *p* < 0.05). Consequently, non-parametric tests were used. To examine the relationship between oral health-related quality of life (OHRQoL) and oral health perception (OHP), Spearman’s Rho correlation coefficient was used, as it is the appropriate statistic for the analysis of ordinal variables. An ordinal logistic regression model was also used to assess the relationship between the variables, including Nagelkerke’s Pseudo R-squared as the measure of fit. Confidence intervals (CIs) were also included, as they contribute to a better approximation of the results obtained in the analysis. As an alternative, the use of the statistical software R (The R Project for Statistical Computing) was also considered. The analysis identified a statistically significant association between the variables studied, providing empirical evidence of the interdependence between oral health-related quality of life (OHRQoL) and perceptions of oral health among hospital healthcare personnel. ^32^ ^–^ ^35^

### Ethical implications

This study was conducted in strict compliance with the principles of scientific integrity and respect for participants’ rights, as established in institutional codes and current ethical regulations. International guidelines for research involving human subjects were considered, such as the guidelines of the Council for International Organizations of Medical Sciences (CIOMS, 2016), ^36^ the recommendations of the Belmont Report (1979), ^37^ and the principles set forth in the Declaration of Helsinki (World Medical Association, 2013). ^38^ The proposal was approved by a duly constituted institutional ethics committee, which evaluated and authorized its execution through a formal resolution issued in the first quarter of 2025. All phases of the study respected the criteria of confidentiality, informed consent, and voluntary participation of participants. Furthermore, all participants voluntarily signed a written informed consent form, which detailed the objectives, procedures, benefits, and risks of the study. Only those who fully understood and accepted these conditions were included. Finally, the university’s ethical policies were respected, as described in the institutional Research Ethics Code, ^39^ which ensured the originality, transparency, and methodological rigor of the research.

## Results

Table 1 it is observed that 38.90% of staff with excellent OHRQoL reported a low level of oral health perception (OHP) at 52.80%, while the 34.70% with poor OHRQoL presented a more balanced distribution across OHP categories. Likewise, a low (r = 0.391), but significant ( *p* = 0.001), positive correlation was evident between OHRQoL and OHP.

**Table 1.** Relationship between Oral Health-Related Quality of Life (OHRQoL) and Oral Health Perception (OHP) among Health Personnel at a Level II-1 Hospital in Northern Peru, 2024.

Oral Health-Related Quality of Life (OHRQoL)	Oral Health Perception (OHP)						Total	
	Low		Regular		Excellent			
	N	%	N	%	N	%	N	%
**Excellent**	21	29.20	6	8.30	1	1.40	28	38.90
**Regular**	8	11.10	1	1.40	10	13.90	19	26.40
**Bad**	9	12.50	6	8.30	10	13.90	25	34.70
**Total**	38	52.80	13	18.10	21	29.20	72	100.00

Rho Spearman	Next	Pseudo R Nagelkerke	Next
0.391	0.001	0.198	0.001

Note: Data processed using IBM SPSS Statistics v25. Includes Spearman’s Rho correlation, significance level ( *p*), and Nagelkerke’s Pseudo R ^2^. Own elaboration.

Likewise, ordinal logistic regression analysis, with a Nagelkerke pseudo R-squared of 0.198 (p = 0.001), confirmed a significant association of QoL on OHP, accounting for 19.80%. To ensure inferential accuracy, confidence intervals (CI) and Nagelkerke pseudo R-squared values were included in all regression tests, as detailed in Tables 1 and 4.

Table 2 the findings show that oral health-related quality of life (OHRQoL) is predominantly grouped into three distinct categories or levels: excellent (38.90%), poor (34.70%), and fair (26.40%). Furthermore, when analyzing the dimensions of oral health-related quality of life (OHRQoL), it is observed that all reached high percentages at the excellent level. Functional limitation was the most common, with 44.40%, followed by physical pain, 52.80% and Psychological distress (54.20%), Physical and psychological disability registered 69.40% ,while Social disability reached 76.40%, finally, handicap (general disability) showed the highest percentage, at 83.30%.

**Table 2.** Quality of Life Level and its dimensions of the health personnel of a Level II-1 Hospital in Northern Peru, 2024.

OHRQoL	OHRQoL Dimensions															
	Functional limitation		Physical pain	Psychological discomfort				Psychological disability		Psychological inability			Social disability	Handicap		
Levels	N	%	N	%	N	%	N	%	N	%	N	%	N	%	N	%
**Excellent**	28	38.90	32	44.40	38	52.80	39	54.20	50	69.40	50	69.40	55	76.40	60	83.30
**Regular**	19	26.40	28	38.90	23	31.90	22	30.60	18	25.00	18	25.00	14	19.40	10	13.90
**Bad**	25	34.70	12	16.70	11	15.30	11	15.30	4	5.60	4	5.60	3	4.20	2	2.80
**Total**	72	100.00	72	100.00	72	100.00	72	100.00	72	100.00	72	100.00	72	100.00	72	100.00

Note: Statistical analysis of oral health perception, processed questionnaires, own elaboration.

Table 3 it indicates that the Oral Health Perception (OHP) is distributed mainly into three levels: low (52.80%), excellent (29.20%), and regular (18.00%). When analyzing the specific dimensions, it was observed that the Perception of Knowledge registered 100% at a low level. In contrast, the Perception of Attitude showed 44.4% at the low level, while Perception of Behavior recorded its highest concentration at the regular level (62.50%).

**Table 3.** Level of oral perception and its dimensions in health personnel from a level II-1 hospital in northern Peru, 2024.

Oral Health Perception (OHP)			(OHP) Dimensions					
			Perception of knowledge		Attitude perception		Perception of behavior	
Levels	N	%	N	%	N	%	N	%
**Low**	38	52.80	72	100.00	32	44.40	15	20.80
**Regular**	13	18.00	0	0.00	31	43.10	45	62.50
**Excellent**	21	29.20	0	0.00	9	12.50	12	16.70
**Total**	72	100.00	72	100.00	72	100.00	72	100.00

Note: Data analyzed using IBM SPSS Statistics v25. Results correspond to the distribution of oral health perception (OHP) and its dimensions based on the validated instrument. Own elaboration.

Table 4 presents a detailed analysis of the relationships between the dimensions of oral health-related quality of life (OHRQoL) and oral health perception (OHP), highlighting key differences in the magnitude of these associations. In particular, the psychological distress dimension showed the stronges connection with OHP, supported by a moderate correlation coefficient (r = 0.421) and a high level of statistical significance ( *p* = 0.000). Additionally, Nagelkerke’s Pseudo R-squared was 0.111 (p = 0.027), indicating that this dimension explains 11.1% of the variability of OHP. The Physical disability, dimension also exhibited a statistically significant association, albeit with a slightly smaller effect size. The correlation coefficient was 0.319 (p = 0.006), and the Pseudo R-squared value reached 0.167 (p = 0.004), suggesting a 16.7% contribution to the variability in OHP. In contrast, the functional limitation dimension demonstrated a much weaker relationship with OHP, yielding a correlation coefficient of 0.096 (p = 0.424) and a Pseudo R-squared of 0.014 (p = 0.649), indicating a negligible effect on the dependent variable.

**Table 4.** Relationship between the dimensions of the Quality of life with the perception of oral health of the staff of a level II-1 hospital in La Libertad, 2024.

Functional limitation	Oral Health Perception (OHP)								Inferential analysis			
	Low		Regular		Excellent		Total		Spearman's Rho	p-value	Nagelkerke's Pseudo R	Next
	N	%	N	%	N	%	N	%				
Excellent	18	25.00	7	9.70	7	9.70	32	44.40	0.096	0.424	0.014	0.649
Regular	15	20.80	3	4.20	10	13.90	28	38.90				
Bad	5	6.90	3	4.20	4	5.60	12	16.70				
**Total**	38	52.80	13	18.10	21	29.20	72	100.00				

Physical pain	OHP						Total					
	Low		Regular		Excellent							
	N	%	N	%	N	%	N	%				
Excellent	24	33.30	7	9.70	7	9.70	38	52.80	0.266	0.024	0.093	0.049
Regular	12	16.70	2	2.80	9	12.50	23	31.90				
Bad	2	2.80	4	5.60	5	6.90	11	15.30				
**Total**	38	52.80	13	18.10	21	29.20	72	100.00				

Psychological discomfort	OHP						Total					
	Low		Regular		Excellent							
	N	%	N	%	N	%	N	%				
Excellent	26	36.10	6	8.30	7	9.70	39	54.20	0.421	0.000	0.111	0.027
Regular	8	11.10	5	6.90	9	12.50	22	30.60				
Bad	4	5.60	2	2.80	5	6.90	11	15.30				
**Total**	38	52.80	13	18.10	21	29.20	72	100.00				

Physical disability	OHP						Total					
	Low		Regular		Excellent							
	N	%	N	%	N	%	N	%				
Excellent	30	41.70	8	11.10	12	16.70	50	69.40	0.319	0.006	0.167	0.004
Regular	8	11.10	5	6.90	5	6.90	18	25.00				
Bad	0	0.00	0	0.00	4	5.60	4	5.60				
**Total**	38	52.80	13	18.10	21	29.20	72	100.00				

Psychological Inability	OHP						Total					
	Low		Regular		Excellent							
	N	%	N	%	N	%	N	%				
Excellent	30	41.70	8	11.10	12	16.70	50	69.40	0.232	0.050	0.167	0.004
Regular	8	11.10	5	6.90	5	6.90	18	25.00				
Bad	0	0.00	0	0.00	4	5.60	4	5.60				
**Total**	38	52.80	13	18.10	21	29.20	72	100.00				

Social Inability	OHP						Total					
	Low		Regular		Excellent							
	N	%	N	%	N	%	N	%				
Excellent	31	43.10	11	15.30	13	18.10	55	76.40	0.242	0.040	0.124	0.017
Regular	7	9.70	2	2.80	5	6.90	14	19.40				
Bad	0	0.00	0	0.00	3	4.20	3	4.20				
**Total**	38	52.80	13	18.10	21	29.20	72	100.00				

Handicap	OHP						Total					
	Low		Regular		Excellent							
	N	%	N	%	N	%	N	%				
Excellent	36	50.00	9	12.50	15	20.80	60	83.30	0.298	0.011	0.131	0.013
Regular	2	2.80	4	5.60	4	5.60	10	13.90				
Bad	0	0.00	0	0.00	2	2.80	2	2.80				
**Total**	38	52.80	13	18.10	21	29.20	72	100.00				

Note: Inferential analysis based on Spearman’s Rho correlation and ordinal logistic regression. Includes p-values and Nagelkerke’s Pseudo R ^2^. Data processed using IBM SPSS Statistics v25. Own elaboration.

Overall, these findings confirm the existence of a positive relationship between quality-of-life dimensions and oral health perception. However, the dimensions of psychological distress and physical disability exhibited stronger associations with OHP, highlighting the need to implement targeted interventions that address these critical psychosocial and functional domains.

## Discussion

Oral health-related quality of life (OHRQoL) and oral health perception (OHP) are essential elements that directly associated with the overall well-being of hospital staff. This study, examined the relationship between these variables was established in a total of 72 workers from a hospital located in northern Peru during the year 2024, with the purpose of identifying and establishing areas of opportunity that allow the execution of strategies that address the specific needs of the group. First, these results suggest in Table 1 show that there is a low-level positive correlation (r = 0.391), but statistically significant (p < 0.05). Furthermore, the analysis showed that OHRQoL accounts for 19.8% of the variability in OHP. Notably the highest frequency of cases corresponds to healthcare personnel who reported an optimal OHRQoL, despite experiencing low levels of OHP (29.2%). These findings are similar to those obtained by Miranda and Alcocer in 2021, who found that the sociodemographic characteristics of older adults association the perception of oral health-related quality of life (OHRQoL) and its link to oral health, without generating significant negative associations. In their study, they identified that a high percentage of participants maintained excellent (45.4%) or moderate (34.6%) levels of oral health-related quality of life (OHRQoL). Therefore, when healthcare professionals experience a favorable oral health-related quality of life (OHRQoL), problems associated with oral health tend to go unnoticed, as they do not significantly interfere with their daily activities or work performance. ^2^ ^,^ ^12^

Table 2 shows that oral health-related quality of life (OHRQoL) among hospital staff was distributed into three main categories: *excellent* (38.9%), *poor* (34.7%), and *fair* (26.4%).

Furthermore, the analysis of the seven OHRQoL dimensions revealed consistently high percentages at the excellent level: *functional limitation* (44.4%), *physical pain* (52.8%), *psychological distress* (54.2%), *physical and psychological disability* (69.4%), *social disability* (76.4%), and *handicap* (83.3%).

These findings are consistent with those reported by Espinoza et al. (2022), who investigated OHRQoL and its association with oral health among residents of a geriatric center in Lima. In their study, 66.8% of participants reported excellent OHRQoL, and oral health problems did not significantly affect their perceived quality of life. ^30^

This trend may reflect the importance that healthcare professionals place on oral care, which is reflected in a positive perception of OHRQoL. Previous literature supports the association between good oral health and higher OHRQoL, as it reduces pain and discomfort while improving functionality and social interaction. ^40^

Table 3 presents the most relevant data on oral health perception (OHP) and its dimensions among healthcare personnel at a hospital in northern Peru. OHP was primarily distributed across three levels: low (52.8%), excellent (29.2%), and regular (18.0%). In the specific dimensions, the low level predominated in the Perception of Knowledge, with 100% of participants in this category. The Perception of Attitude registered 44.4% at the low level, while the Perception of Behavior was mostly categorized as regular, with 62.5%. These findings contrast with previous studies, such as that by López (2021), which reported that the level of knowledge regarding OHP among healthcare workers at EsSalud Hospital II during the COVID-19 pandemic was high (81.5%), indicating a solid command of this dimension. ^41^ The limited perception of knowledge observed in this study may be attributed to the lack of continuous oral health (OH) training programs targeting healthcare personnel. This aligns with prior research emphasizing that training and education are essential for reinforcing knowledge and promoting adequate occupational health practices. Moreover, attitude and behavior are not only influenced by knowledge but also by cultural beliefs and traditions concerning occupational health, which may impact the adoption of preventive habits and the pursuit of timely treatment. ^42^ ^,^ ^43^

Table 4 presents the results regarding the relationship between functional limitation and perceived oral health in hospital personnel. The highest proportion corresponded to those who reported excellent oral health-related quality of life (OHRQoL) but low oral health perception (OHP), representing 25.0%. However, statistical analysis indicated no significant correlation (r = 0.096) or relevant association between this dimension and the variable (p > 0.05).

When comparing these results with previous research, similar patterns are observed. García-Cortés et al. (2020) reported that OHP did not show a statistically significant relationship with OHRQoL in a study conducted with healthcare professionals in Spain. ^44^ Likewise, López-Jiménez et al. (2019) found that, although functional limitations were present, these did not significantly affect OHP in a sample of nurses in Mexico. ^45^

In contrast, other studies conducted in different populations have shown significant associations between these variables. For instance, Martínez-Rodríguez et al. (2018) found that functional limitations negatively impacted OHP in a sample of older adults in Chile. ^46^

Regarding the association between physical pain and the perception of oral health among workers, a low-intensity positive correlation was identified (r = 0.266), with a limited explanatory power of 9.3% for this dimension on OHP. Notably, the highest proportion was recorded among workers with excellent OHRQoL but low OHP (33.3%). These results align with those reported by Campos et al. (2014), who analyzed the impact of oral conditions on job performance and found that physical pain had a negative association of 82.9% with occupational outcomes. ^47^ This suggests that the absence of oral pain may lead individuals to underestimate or overlook their oral health perception. ^48^

In turn, the relationship between psychological distress and OHP revealed a moderate positive correlation (r = 0.421) with high statistical significance (p < 0.01), and an explanatory power of 11.1%. Interestingly, the highest percentage was observed in individuals with excellent OHRQoL but low OHP (10.4%). These findings are consistent with Espinoza (2017), who reported a negative association of 61.4% between psychological distress and OHP. ^30^ The data suggest that the absence of psychological distress may similarly cause workers to underestimate their oral health status. ^48^

As for the relationship between physical disability and OHP, a statistically significant correlation of low magnitude was found (r = 0.319, p < 0.05), explaining 16.7% of the variation in OHP. These findings contrast with those of Bellamy and Moreno (2014), who reported that physical disability was one of the most affected dimensions in patients with removable prostheses and tooth loss, highlighting the importance of oral function in perceived well-being. ^49^ Thus, when there is no physical disability, OHP goes unnoticed; and, conversely, if this dimension is present, oral health is prioritized. ^50^

Regarding the relationship between psychological disability and OHP among healthcare workers, a low positive correlation was observed (r = 0.232), with a statistically significant association of 16.7%. Notably, the highest proportion was recorded among those with excellent OHRQoL but low OHP (41.7%). Additionally, when psychological disability was classified at a regular level, OHP was also distributed between regular and excellent categories (6.9% each). These findings are consistent with Espinoza (2017), who identified a 31.5% inverse association between oral health and psychological disability. ^30^ This suggests that in the absence of psychological disability, oral health perception tends to be deprioritized, whereas its presence may promote a greater awareness and engagement with oral health.

In terms of social disability and its association with OHP, a statistically significant yet weak correlation was found (r = 0.242, p < 0.05), explaining 12.4% of the variation in perceived oral health. Interestingly, the highest proportion was recorded among workers with excellent OHRQoL but low OHP (43.1%). Conversely, when social disability was poor, OHP was consistently excellent (4.2%). These data align with findings by Espinoza (2017), who reported a 23.4% inverse relationship between social disability and OHRQoL. ^30^ Therefore, a lack of social disability may reduce the perceived relevance of oral health, while its presence can lead to more proactive health-seeking behaviors.

Finally, regarding general disability (handicap) and its link with OHP, a statistically significant low-magnitude correlation was found (r = 0.298, p < 0.05), with an association of 13.1%. The highest percentage was observed in workers with excellent OHRQoL and low OHP (50.0%). In cases of moderate disability, OHP was distributed equally between excellent and regular levels (5.6% each). These results are comparable to those reported by Espinoza et al. (2022), who found a 17.0% negative association between oral health and perceived disability. ^30^ This indicates that when workers do not perceive limitations, they may overlook oral health needs; in contrast, the presence of disability can encourage the adoption of specific care strategies and behaviors aimed at maintaining oral health. ^51^

### Limitations of the study

The discussion of this study is framed by certain methodological limitations related to its design and sample size. As the research was conducted in a single Level II-1 hospital in northern Peru, the findings cannot be extrapolated to broader populations or other geographic settings due to the contextual particularities of the sample and institution. Similarly, the limited sample size of 72 participants constrains the generalizability of the findings to other healthcare professionals.

Moreover, this research employed instruments that were specifically validated to measure oral health-related quality of life (OHRQoL) and perceived oral health (POH) in healthcare personnel. While these tools enhance validity and comparability, they are inherently limited by their reliance on self-reported data. Self-assessment methods, although standardized and widely accepted, may introduce subjectivity and social desirability bias in responses.

Another key limitation lies in the cross-sectional design of the study. Since data were collected at a single point in time, causal relationships between OHRQoL and POH cannot be established. Nonetheless, the findings offer valuable insight for future longitudinal research aimed at exploring how these variables evolve over time and across healthcare settings.

Finally, while the sample was adequate for the study’s scope, expanding the research to include other hospitals in different regions or countries—such as in Spain or other Latin American contexts—would strengthen the external validity of the results and help identify consistent patterns or divergences in similar healthcare environments.

### Implications of the study

The findings underscore the importance of oral health as one of the core elements of the overall well-being of hospital staff. The existence of a significant correlation between oral health-related quality of life (OHRQoL) and psychological well-being, along with the involvement of certain dimensions such as physical disability, psychosocial disability, etc., highlights the need for interventions that address oral health as well as psychosocial factors, determinants of personal well-being and, therefore, of the quality of service offered to patients. Finally, although the results can be used to guide the development of strategies in other similar contexts, their implications should be considered with caution and within the framework of the local context under study. This encourages the adoption of multidimensional and collaborative approaches in hospital settings that would empower them to promote sustainable development and well-being.

## Conclusion

The study demonstrates a statistically significant correlation between oral health-related quality of life (OHRQoL) and oral health perception (OHP) among healthcare professionals at a Level II-1 hospital in northern Peru. The Spearman’s correlation coefficient was 0.391, and Nagelkerke’s pseudo R-squared was 0.198, both with statistical significance (p = 0.001). Higher levels of OHRQoL were significantly associated with more favorable OHP. In addition, meaningful associations were observed in the dimensions of physical disability (Rho = 0.319, p < 0.05; Nagelkerke = 0.167), psychological disability (Rho = 0.232, p = 0.05; Nagelkerke = 0.167), social disability (Rho = 0.242, p < 0.05; Nagelkerke = 0.124), and general disability (Rho = 0.298, p < 0.05; Nagelkerke = 0.131). These results suggest that strengthening factors that contribute positively to OHRQoL could lead to more favorable perceptions of oral health among hospital staff. Thus, this evidence offers contextual support that may serve as a reference for similar institutional environments, while acknowledging the influence of sociocultural differences in each setting.

### Recommendations

**Develop continuing education programs:** Design and implement educational strategies focused on oral health for hospital personnel. These programs should emphasize the importance of oral hygiene, prevention of oral diseases, and their relationship with oral health-related quality of life (OHRQoL) and professional performance. Integration into existing workplace wellness programs is recommended to ensure sustainability and institutional support.

**Enhance access to dental services:** Establish in-hospital dental services with flexible hours that accommodate healthcare staff work shifts. Facilitating timely preventive care and treatment may contribute to improving perceptions and outcomes related to oral health.

**Monitor oral health and OHRQoL:** Incorporate validated instruments such as the OHIP-14 and HU-DBI into regular assessments of hospital staff. This approach would enable systematic evaluation of the impact of oral health interventions, while supporting a comprehensive strategy to promote occupational well-being.

## Data availability

### Underlying data

Regarding the availability of the data used, these are publicly accessible on the Zenodo platform, under the terms of the Creative Commons Attribution 4.0 International (CC-BY 4.0) license.

The main database can be consulted at: https://doi.org/10.5281/zenodo.14847738

This file in Excel format (.xls) contains the anonymized responses to the Quality of Life (OHRQoL) and Oral Health Perception (OHP) questionnaires applied to hospital staff. ^52^

The complementary methodological appendix, which includes the complete instruments (OHIP-14 and HU-DBI in Spanish), as well as the expert judgment validation matrix and the reliability levels obtained, is available at: https://doi.org/10.5281/zenodo.15236712. ^31^

These files ensure the transparency and reproducibility of the data collection process.
