# Relationship between the perception of oral health and the quality of life of hospital staff

**DOI:** 10.12688/f1000research.161146.4

**Published:** 2025-10-13

**Authors:** Miryam Lora Loza, Sheyla del Pilar Alvarado-Romero, Katia Ninozca Flores Ledesma, Nancy Cuenca Robles, David Rene Rodríguez Díaz

**Affiliations:** 1 Graduate School, César Vallejo University, Lima, 15311, Peru; 2 School of Human Medicine, Private University of the North, Trujillo, Lima Norte, 13001, Peru

**Keywords:** Quality of life, Perception, Oral health, Correlation, Hospital, Functional limitation.

## Abstract

**Introduction:**

Hospital staff’s perception of oral health directly impacts their overall oral health-related well-being (OHRQoL) and their job performance. This study seeks to analyze the relationship between these two dimensions, providing information for designing strategies that promote a healthier work environment.

**Aim:**

To determine the relationship between oral health-related quality of life (OHRQoL) and oral health perceptions in the staff of a level II-1 hospital located in northern Peru.

**Methods:**

The study had a quantitative approach, with a cross-sectional, applied, and correlational design. Seventy-two participants participated. The validated OHIP-14 and HU-DBI questionnaires were used, with reliability coefficients of 0.847 and 0.804, respectively. Spearman’s correlation coefficient, appropriate for ordinal variables, was used for data analysis.

**Results:**

A statistically significant association was found between health-related quality of life and subjective perception of oral status (Rho = 0.391, p < 0.05), with an explained variance of 19.8% according to Nagelkerke’s pseudo R-squared. The most frequently associated quality of life dimensions were physical disability (Rho = 0.319; p < 0.05) and social disability (Rho = 0.242; p < 0.05). Excellent quality of life was the most prevalent (38.9%), while poor oral health was the most common (52.8%).

**Conclusion:**

The findings show a significant relationship between self-perceived oral health and oral health-related quality of life in this group of professionals. Promoting oral health strategies tailored to the hospital setting is recommended to improve workplace well-being.

**Abreviaturas**
FDI: Federación Dental Internacional
UNESCO: Organización de las Naciones Unidas para la Educación la Ciencia y la Cultura
PNUD: Programa de las Naciones Unidas para el Desarrollo

## Introducción

Oral health is an essential component of overall well-being and is directly associated with oral health–related quality of life (OHRQoL) through the ability to perform basic functions such as communicating, eating properly, and maintaining satisfactory social relationships. ^1^ Oral diseases affect not only oral functions but also psychological, social, and economic outcomes, causing discomfort, pain, and loss of self-esteem, and shaping the subjective perception of oral well-being. ^2^^,^^3^ The association between oral health and OHRQoL underscores its direct contribution to achieving Sustainable Development Goal 3 (good health and well-being), in which oral health is a fundamental component.

Within this framework, oral health is considered a global priority by international organizations such as the FDI World Dental Federation (FDI) and the World Health Organization (WHO), given its clear relationship with OHRQoL. The WHO conceptualizes oral health as a comprehensive state that encompasses physical, mental, and social well-being in relation to the functionality and condition of the oral cavity, not merely the absence of disease (WHO, 2022), while FDI Vision 2030 (2021) advances an integrative perspective that links oral well-being with sustainable public health policies. ^4^^,^^5^

Oral diseases constitute a global public health problem affecting approximately 3.5 billion people worldwide, with higher prevalence in low- and middle-income countries, where about 75% of cases are concentrated. ^4^ Untreated dental caries is the most prevalent condition, reflecting deep inequities in access to preventive services and basic treatments. ^6^^,^^7^ In Latin America, periodontal diseases represent a widespread burden that significantly impairs OHRQoL. In countries such as Peru, the magnitude of the problem is exacerbated by the low priority given to oral health within health agendas, evidenced by limited public investment and elevated oral cancer rates (2.60 per 100,000 women and 1.97 per 100,000 men between 2000 and 2017). ^8^ This scenario is associated with constrained resource allocation, the absence of effective preventive strategies, and insufficient early-detection programs. ^9^ In response, WHO and other organizations have promoted key initiatives—most notably FDI Vision 2030 and the 2021 World Health Assembly Resolution on oral health—which emphasize integrating oral health into Universal Health Coverage (UHC) and aligning actions with the global agenda against non-communicable diseases (NCDs).

According to the WHO Global Oral Health Status Report (2022), ^4^ it is essential to reorient public policies to prioritize the promotion and production of scientific knowledge in oral health through national plans aligned with WHO’s strategic vision. ^9^ Collaboration with institutions such as UNESCO and UNDP is also relevant to embed oral health determinants within well-being and sustainable human development policies. ^10^^,^^11^ Among healthcare personnel, **perceived oral health (POH)** can be measured and has been associated not only with oral well-being but also with **OHRQoL**. POH may be influenced by contextual factors such as limited accessibility to dental services and workload. ^12^^–^^14^ Negative POH can affect self-esteem and interpersonal relationships, which justifies evaluating staff well-being using OHRQoL metrics. ^8^^,^^15^

**Purpose.** This study examines the relationship between **OHRQoL** and **POH** among staff at a Level II-1 hospital in northern Peru, considering their dimensions and interactions. The findings provide evidence to guide strategies that promote the oral well-being of healthcare staff and, consequently, support comprehensive, patient-centered care based on quality criteria. ^16^^,^^17^

## Methods

**Research type and design.** This study employed **applied research** aimed at solving a specific problem in order to propose solutions to the challenges faced by hospital staff. ^18^^–^^20^ The research scope was **correlational**, as it sought to identify the relationship between the variables and analyze the strength and direction of this relationship. ^21^ A **non-experimental, cross-sectional** design was adopted, which allowed data to be collected at a single point in time without manipulating the variables, thus preserving the **natural context** of observation. ^22^

**Population.** The group was initially comprised of **80** members of the professional and technical team providing care at a **Level II-1** healthcare facility in northern Peru. This group included **21 physicians, 10 obstetricians, 1 dentist, 1 pharmaceutical chemist, 17 nurses, 2 psychologists, 3 biologists, 5 microbiologists, 2 medical technologists, 13 nursing technicians, 4 pharmacy technicians, and 1 laboratory technician**. After applying the **inclusion criteria** —designated or assigned personnel and CAS (Administrative Contracting of Services), ≥6 months of seniority, agreement to complete both questionnaires, and signed/stamped informed consent—the **exclusion criteria** considered workers with <6 months of seniority, vacation or sick leave, outsourced modality, those carrying out **SERUMS** (Rural and Marginal Urban Health Service), and those who did not agree to participate. A **non-probabilistic intentional convenience sampling** strategy was used, obtaining a final sample of **n = 72** participants, which allowed the collection of sufficient data for correlational analyses given feasibility and accessibility during the study period. Additionally, to estimate the sample size, a power analysis (G*Power) was performed, establishing as parameters an expected **medium correlation (ρ = 0.30)**, **95% confidence level**, and **80% power**. Under these parameters, the minimum required size was **67** participants; therefore, the achieved sample ( **n = 72**) was adequate for the planned correlational analysis. ^23^

**Variables.** Two main variables were evaluated: **oral health–related quality of life (OHRQoL)** and **perceived oral health (POH)**. **OHRQoL** is conceived as the individual’s appraisal of how oral conditions are associated with different aspects of daily life—physical, emotional, and social. ^24^ For its assessment, the **Oral Health Impact Profile (OHIP-14)** developed by Slade and Spencer was used. This instrument comprises **14 items** organized into **seven domains**: functional limitation, psychological discomfort, physical pain, psychological disability, physical disability, social disability, and handicap. The overall score indicates the extent to which oral health is associated with OHRQoL, without extending to general QoL.

**POH** refers to the **subjective evaluation** of oral status and its relationship with QoL, ^25^ and in this study it was addressed using a **modified version of the Hiroshima University–Dental Behavioral Inventory (HU-DBI)**, initially developed by Kawamura (1988) and adapted to the Peruvian context by Midolo (2023). ^26^^,^^27^ This version was internally validated by **Alvarado and Lora (2024)** for healthcare personnel. The instrument considers **three dimensions**: perception of knowledge, perception of behavior, and perception of attitude. POH served as an indicator of awareness, disposition, and practice regarding one’s oral health.

**Data collection technique and instruments.** The **survey** technique was used to facilitate systematic data collection from hospital professionals and technical staff via standardized questionnaires, **without altering** the environment or the object of study. ^28^

For **OHRQoL**, the **OHIP-14**—originally designed by Slade and Spencer (1994), adapted by **Espinoza (2017)** ^29^ and subsequently validated and published by **Espinoza et al. (2022)** ^30^—was administered (14 items; seven domains; **Likert 0 = never to 4 = very frequently**). For interpretability, three categories were used following Espinoza’s methodological criteria: **excellent (0–2 points)**, **regular (3–9 points)**, and **poor (≥10 points)** OHRQoL.

For **POH**, the **modified HU-DBI** ^31^^,^^32^ was used, validated for hospital settings by **Alvarado and Lora, 2024**. It consists of **20 dichotomous items** (Yes = 1; No = 0) across the three dimensions noted above, and total scores were categorized as **poor (0–9)**, **regular (10)**, and **excellent (11–20)**.

**Content validity** for both instruments was established by a five-member expert panel, assessing internal consistency, conceptual clarity, thematic relevance, and informative sufficiency, yielding **Aiken’s V = 1.00**. **Reliability** from the pilot showed **Cronbach’s α = 0.847** (OHIP-14) and **α = 0.804** (HU-DBI), indicating acceptable internal consistency.

For methodological transparency and replication, all materials ( **OHIP-14**, modified **HU-DBI **, expert-review matrix, and pilot reliability outputs) are publicly available on **Zenodo**: https://doi.org/10.5281/zenodo.15236712. ^31^

**Procedure.** The study began with a formal cover letter from the **Graduate School, Universidad César Vallejo**, to the administrative department of the La Libertad Level II-1 Hospital requesting authorization to administer the instruments. After approval, on-site sessions were scheduled; the objectives, procedures, confidentiality safeguards, and anonymity were explained, and participants provided **written informed consent**. Instruments were completed in discrete areas within workspaces and required approximately **8 minutes** per person, minimizing distractions and contextual bias.

**Information analysis.** Statistical analysis was performed in **IBM SPSS Statistics v25** ( https://www.ibm.com/products/spss-statistics ). Normality was tested with **Kolmogorov–Smirnov**; results indicated non-normality ( **p < 0.05**), so **non-parametric** tests were used. Associations between **OHRQoL** and **POH** were examined with **Spearman’s ρ** (ordinal data). **Ordinal logistic regression** was then fitted; **Nagelkerke’s pseudo-R** ^**2**^ and **95% confidence intervals** were reported. As an alternative, **R** (The R Project for Statistical Computing) was considered. The analysis identified a statistically significant association between **OHRQoL** and **POH** among hospital staff. ^32^^–^^35^

**Ethics.** The study protocol, entitled *“Quality of life and perception of oral health of the staff of a Level II-1 hospital in La Libertad, 2024”*, received a **favorable opinion** from the **Research Ethics Committee of the Master’s Program in Health Services Management, Universidad César Vallejo** (Report No. 00298-2024/CEI-PMGSS, January 30, 2025). The committee authorized its implementation in accordance with institutional and international ethical standards, including **CIOMS (2016)**, ^38^ the **Belmont Report (1979)**, ^39^ and the **Declaration of Helsinki (WMA, 2013)**. ^40^ All participants signed **written informed consent** before data collection, ensuring confidentiality, voluntary participation, and the right to withdraw at any time. Furthermore, the study followed the university’s **Research Ethics Code**, ^41^ guaranteeing originality, transparency, and methodological rigor throughout all phases.

## Resultados

**Overall findings.** Among staff, **OHRQoL** showed a predominance of the **“Excellent”** category ( **38.90%**), while **POH** concentrated at the **regular** level ( **62.50%**). At the bivariate level, **OHRQoL and POH were positively associated** ( **Spearman’s ρ = 0.391; p = 0.001**), indicating that better perceived oral health was linked to better OHRQoL.

**Table 1.** Relationship between Oral Health–Related Quality of Life (OHRQoL) and Oral Health Perception (POH) among Health Personnel at a Level II-1 Hospital in Northern Peru, 2024.

Oral Health–Related Quality of Life (OHRQoL)	Oral Health Perception (POH)						Total	
	Low		Fair		Excellent			
	n	%	n	%	n	%	n	%
**Excellent**	21	29.20	6	8.30	1	1.40	28	38.90
**Fair**	8	11.10	1	1.40	10	13.90	19	26.40
**Poor**	9	12.50	6	8.30	10	13.90	25	34.70
**Total**	38	52.80	13	18.10	21	29.20	72	100.00

Spearman’s ρ	p	Nagelkerke pseudo R ^2^	p
0.391	0.001	0.198	0.001

Note: Ordinal logistic regression analysis, with a Nagelkerke pseudo R ^2^-squared of **0.198** ( *p* = 0.001), confirmed a **significant association** between **OHRQoL** and **POH**, explaining **19.80%** of the variance. To ensure **inferential accuracy**, both **confidence intervals (CI)** and **Nagelkerke pseudo R-squared** values were included in all regression tests, as detailed in **Tables 1** **and** **4**.

**Table 2.** Level of oral health–related quality of life (OHRQoL) and its OHIP-14 domains among hospital staff at a Level II-1 facility in northern Peru.

OHRQoL level	Dimensions of Oral Health–Related Quality of Life (OHRQoL)													
	Functional limitation		Physical pain		Psychological discomfort		Psychological disability		Physical disability		Social disability		Handicap	
Levels	n	%	n	%	n	%	n	%	n	%	n	%	n	%
**Excellent**	28	38.90	32	44.40	38	52.80	39	54.20	50	69.40	55	76.40	60	83.30
**Fair**	19	26.40	28	38.90	23	31.90	22	30.60	18	25.00	14	19.40	10	13.90
**Poor**	25	34.70	12	16.70	11	15.30	11	15.30	4	5.60	3	4.20	2	2.80
**Total**	72	100.00	72	100.00	72	100.00	72	100.00	72	100.00	72	100.00	72	100.00

**Table 3.** Level of oral health perception (POH) and its dimensions among healthcare staff at a Level II-1 hospital in northern Peru, 2024.

Oral Health Perception (POH)			Dimensions (POH)					
			Knowledge Perception		Attitude Perception		Behavior Perception	
Levels	n	%	n	%	n	%	n	%
**Low**	38	52.80	72	100.00	32	44.40	15	20.80
**Fair**	13	18.00	0	0.00	31	43.10	45	62.50
**Excellent**	21	29.20	0	0.00	9	12.50	12	16.70
**Total**	72	100.00	72	100.00	72	100.00	72	100.00

**Table 4.** Association between OHIP-14 dimensions and oral health perception (POH) among healthcare personnel at a Level II-1 hospital in La Libertad.

Functional limitation	Handicap								Inferential analysis			
	Low		Fair		Excellent		Total		Spearman’s ρ	p-value	Nagelkerke pseudo R ^2^	*p*
	n	%	n	%	n	%	n	%				
Excellent	18	25.00	7	9.70	7	9.70	32	44.40	0.096	0.424	0.014	0.649
Fair	15	20.80	3	4.20	10	13.90	28	38.90				
Poor	5	6.90	3	4.20	4	5.60	12	16.70				
**Total**	38	52.80	13	18.10	21	29.20	72	100.00				

	Handicap											
	Low		Fair		Excellent		Total					
	n	%	n	%	n	%	n	%				
Excellent	24	33.30	7	9.70	7	9.70	38	52.80	0.266	0.024	0.093	0.049
Fair	12	16.70	2	2.80	9	12.50	23	31.90				
Poor	2	2.80	4	5.60	5	6.90	11	15.30				
**Total**	38	52.80	13	18.10	21	29.20	72	100.00				

	Malestar psicológico											
	Low		Fair		Excellent		Total					
	n	%	n	%	n	%	n	%				
Excellent	26	36.10	6	8.30	7	9.70	39	54.20	0.421	0.000	0.111	0.027
Fair	8	11.10	5	6.90	9	12.50	22	30.60				
Poor	4	5.60	2	2.80	5	6.90	11	15.30				
**Total**	38	52.80	13	18.10	21	29.20	72	100.00				

	Physical disability											
	Low		Fair		Excellent		Total					
	n	%	n	%	n	%	n	%				
Excellent	30	41.70	8	11.10	12	16.70	50	69.40	0.319	0.006	0.167	0.004
Fair	8	11.10	5	6.90	5	6.90	18	25.00				
Poor	0	0.00	0	0.00	4	5.60	4	5.60				
**Total**	38	52.80	13	18.10	21	29.20	72	100.00				

	Psychological disability											
	Low		Fair		Excellent		Total					
	n	%	n	%	n	%	n	%				
Excellent	30	41.70	8	11.10	12	16.70	50	69.40	0.232	0.050	0.167	0.004
Fair	8	11.10	5	6.90	5	6.90	18	25.00				
Poor	0	0.00	0	0.00	4	5.60	4	5.60				
**Total**	38	52.80	13	18.10	21	29.20	72	100.00				

	Social disability											
	Low		Fair		Excellent		Total					
	n	%	n	%	n	%	n	%				
Excellent	31	43.10	11	15.30	13	18.10	55	76.40	0.242	0.040	0.124	0.017
Fair	7	9.70	2	2.80	5	6.90	14	19.40				
Poor	0	0.00	0	0.00	3	4.20	3	4.20				
**Total**	38	52.80	13	18.10	21	29.20	72	100.00				

	Handicap											
	Low		Fair		Excellent		Total					
	n	%	n	%	n	%	n	%				
Excellent	36	50.00	9	12.50	15	20.80	60	83.30	0.298	0.011	0.131	0.013
Fair	2	2.80	4	5.60	4	5.60	10	13.90				
Poor	0	0.00	0	0.00	2	2.80	2	2.80				
**Total**	38	52.80	13	18.10	21	29.20	72	100.00				

Table 2 shows that oral health–related quality of life (OHRQoL) was primarily distributed across three levels: **Excellent (38.90%)**, **Regular (26.40%)**, and **Poor (34.70%)**. Among the **OHIP-14** domains, **social disability (76.40%)** and **handicap (83.30%)** reached the highest percentages in the **Excellent** level, while **functional limitation** recorded **44.40%** in the **Poor** level. This pattern reveals heterogeneity across domains, with greater impact on basic functions despite comparatively favorable social and global performance. Overall, the results suggest prioritizing interventions targeted by dimension (e.g., education for self-care, pain management, and functional rehabilitation) along with general strategies for the promotion of oral health among hospital staff.

Table 3 shows that oral health perception (POH) was primarily distributed across three levels, with a predominance of the Low level (52.80%), followed by Excellent (29.20%) and Regular (18.00%). When analyzing the dimensions, Knowledge Perception showed 100.00% at the Low level, revealing a critical knowledge gap among the evaluated staff. In contrast, Attitude Perception presented 44.40% at the Low level and 43.10% at the Regular level, reflecting a polarization between incipient and moderate attitudes. Meanwhile, Behavior Perception concentrated its highest proportion at the Regular level (62.50%), suggesting acceptable but suboptimal habitual practices.

This pattern of markedly low knowledge, intermediate attitudes, and mostly regular behaviors indicates a knowledge–attitude–behavior gap associated with unstructured learning processes and operational barriers (e.g., service access times, organizational culture). Overall, the findings highlight the need for a stepwise intervention approach: strengthening knowledge (targeted training), reframing attitudes (motivational messaging and service standards), and reinforcing behaviors (reminders, access facilities, follow-up).

**Table 4** presents an analysis of the associations between the OHIP-14 dimensions and oral health perception (POH) (Spearman’s coefficient and ordinal logistic regression). The *psychological discomfort* dimension showed the highest correlation with POH (ρ = 0.421; p < 0.001) and a Nagelkerke pseudo R ^2^ = 0.111; p = 0.027. *Physical disability* also presented a significant association (ρ = 0.319; p = 0.006) with pseudo R ^2^ = 0.167; p = 0.004. *Social disability* and *handicap* revealed moderate associations (ρ = 0.242; p = 0.040 and ρ = 0.298; p = 0.011, respectively) with pseudo R ^2^ = 0.124; p = 0.017 and pseudo R ^2^ = 0.131; p = 0.013, underscoring their predictive relevance for POH. In contrast, *functional limitation* showed a weak and non-significant relationship (ρ = 0.096; p = 0.424; pseudo R ^2^ = 0.014; p = 0.649), indicating a minimal contribution to POH variability.

Overall, the findings confirm a positive association between the OHIP-14 dimensions and POH, with the greatest magnitudes in *psychological discomfort* and *physical disability*; therefore, psychosocial and functional interventions are prioritized for healthcare personnel.

## Discusión

Oral health–related quality of life (OHRQoL) and oral health perception (POH) are essential determinants of healthcare personnel’s overall well-being. This study analyzed the relationship between these variables in 72 healthcare workers from northern Peru in 2024, aiming to identify priority areas for oral health interventions.

**Table 1** revealed a significant association between OHRQoL and POH: 38.9% of participants with excellent OHRQoL reported a high perception of their oral health, whereas 34.7% of those with poor OHRQoL reported a low POH. The positive correlation (Spearman’s ρ = 0.391; p = 0.001) explained 19.8% of the variance according to the Nagelkerke pseudo R ^2^, highlighting the interdependence of clinical and subjective oral health factors.

These findings align with Díaz-Reissner et al., ^13^ who demonstrated that sociodemographic and clinical determinants significantly influence OHRQoL and, consequently, POH. However, Espinoza et al. (2022) reported a 66.8% prevalence of excellent OHRQoL, markedly higher than the 38.9% observed in our study, possibly due to contextual differences such as institutional infrastructure, access to dental services, and occupational health policies. Similarly, the World Health Organization emphasizes that oral health is intrinsically linked to overall well-being, affecting both quality of life and subjective health perceptions. ^43^

Taken together, these results underscore the importance of designing **multilevel interventions**: improving access to preventive dental care, implementing workplace-based oral health programs, and providing continuous education on oral self-care practices. Such strategies could enhance OHRQoL, promote preventive behaviors, and contribute to better occupational performance among healthcare personnel.

Regarding **Table 2**, the staff’s oral health–related quality of life ( **OHRQoL**) was classified mainly as **Excellent (38.9%)**, followed by **Poor (34.7%)** and **Regular (26.4%)**. The domains with the highest proportions within the **Excellent** level were **social disability (76.4%)** and **handicap (83.3%)**, whereas **functional limitation** showed lower values. Although this suggests the presence of some functional constraints, the overall pattern indicates a reduced social and participation impact, pointing to good integration and adaptation. This situation may be explained by the fact that healthcare professionals typically have greater access to information, resources, and dental services, which facilitates maintaining adequate oral health and minimizes its social impact. The literature supports that better oral health is associated with **reduced physical pain, improved function, and better performance in daily and work activities**, ^2^ consistent with the positive perception observed in most personnel. Similar findings were reported by Espinoza et al. ^30^^,^^44^ in a geriatric center in Lima and by Díaz-Reissner et al., ^13^^,^^42^ who emphasize that clinical and sociodemographic factors significantly influence OHRQoL, reinforcing the need to consider both **functional** and **psychosocial** aspects in interventions. In sum, maintaining adequate oral care and access to dental resources benefits not only functional status but also the social integration of hospital staff, strengthening their quality of life and professional performance. ^2^^,^^13^^,^^30^

**Additionally,** **Table 3** presents the distribution of **oral health perception (POH)** and its dimensions among healthcare personnel in northern Peru. POH was mainly concentrated in the **Low** level ( **52.8%**), followed by **Excellent** ( **29.2%**) and **Regular** ( **18.0%**). At the dimensional level, **knowledge** was the category with the highest proportion in the **Low** level, revealing limitations in the understanding of basic oral health concepts. This predominance of low perceptions may be related to the absence of permanent programs for professional development and training in oral health in the regional context. Organizational constraints, limited access to information, and lack of incentives for continuous education could contribute to maintaining low perceptions and restricted knowledge among personnel.

Franco-Giraldo emphasizes that **oral health should be approached as an integral part of systemic and public health**, reinforcing the importance of professional training to modify attitudes and preventive practices. ^2^ However, these results contrast with **López García**, ^45^ who reported that **81.5%** of healthcare staff at the *EsSalud Hospital II – Abancay* had a high level of POH knowledge during the COVID-19 pandemic, suggesting **contextual, organizational, or informational differences**. Likewise, reviews such as that of Pineda Rivera et al. ^46^ point out that deficits in oral health knowledge and attitudes are common among healthcare personnel in the absence of systematic continuing education programs, and Espinoza et al. ^30^ highlight that continuous education is essential to **strengthen knowledge, modify attitudes, and improve preventive practices**.

Overall, these findings highlight the **urgency of implementing educational strategies and occupational health policies** that promote better oral health perception and knowledge, integrating culturally relevant content and reinforcing continuous training as a central axis of intervention. ^2^^,^^30^^,^^46^

**Similarly,** **Table 4** presents a detailed analysis of the relationships between the **dimensions of oral health–related quality of life (OHRQoL)** and **oral health perception (POH)** among hospital staff.

**Functional limitation** showed the highest proportion among participants with excellent OHRQoL but low POH, although the correlation was not statistically significant (ρ = 0.096; p > 0.05). This finding is consistent with **López García**, ^45^ who reported that actual oral health status or knowledge does not always translate into favorable perceptions, and with **Remuzgo Huamán and Remuzgo Huamán**, ^52^ who argue that administrative barriers and insufficient public policy management can mediate the relationship between functional status and perception in healthcare professionals. Conversely, Espinoza et al. ^30^ observed in older adults that functional limitations negatively impacted POH, suggesting that age and comorbidities may strengthen this association in specific populations. Other studies on healthcare personnel (López-Jiménez et al. ^48^) and vulnerable groups (León Mantero et al. ^50^; **Carrillo Espichán** ^51^) also highlight the influence of organizational and contextual factors on oral health perception and quality of life.

Regarding **physical pain**, the analysis revealed a **low but significant positive correlation** (ρ = 0.266) explaining 9.3% of the model variability (Nagelkerke pseudo R ^2^ = 0.093). The highest proportion was found among workers with excellent OHRQoL but low POH, suggesting that the absence of pain may reduce awareness of oral health care needs. Similar results were reported by Campos Guerra et al., ^49^ who documented a strong negative association between oral pain and job performance, emphasizing the influence of physical discomfort on health perception. Espinoza et al. ^30^ further suggested that individuals without oral pain may underestimate their oral health status since discomfort often triggers health-seeking behaviors.

**Psychological discomfort** showed a **moderate positive correlation** with POH (ρ = 0.421; p < 0.001), explaining 11.1% of its variability. The highest frequency was again observed among participants with excellent OHRQoL but low POH, indicating that the absence of psychological discomfort could reduce oral health awareness. These findings align with Espinoza et al., ^30^ who reported a negative association between psychological discomfort and POH, emphasizing the psychological determinants of health perception.

**Physical disability** was also significantly correlated with POH (ρ = 0.319; p < 0.05), explaining 16.7% of the variation. Our findings differ from **Bellamy Ortiz and Moreno**, ^47^ who identified physical disability as one of the most affected dimensions among patients with removable dentures and tooth loss—populations in which oral functionality plays a central role in health perception. In our context, the absence of physical disability may reduce attention to oral health, whereas its presence may increase awareness and prioritization of oral care needs.

**Psychological disability** showed a **low positive correlation** with POH (ρ = 0.232; p < 0.050), explaining 16.7% of the variation. The highest proportion was recorded among participants with excellent OHRQoL but low POH, suggesting that in the absence of psychological impairment, oral health may be perceived as less relevant. These findings are consistent with Espinoza et al., ^30^ who found an inverse association between psychological disability and oral health, indicating that mental well-being mediates the subjective evaluation of health status.

**Social disability** exhibited a **weak but statistically significant correlation** with POH (ρ = 0.242; p < 0.05), explaining 12.4% of its variability. Workers with excellent OHRQoL but low POH predominated in this category, while those with low social disability frequently reported excellent oral health perception. Espinoza et al. ^30^ documented an inverse relationship between social disability and POH, suggesting that social restrictions may intensify health problems and motivate preventive behaviors.

Finally, **general disability (handicap)** showed a **low but significant association** with POH (ρ = 0.298; p < 0.05), explaining 13.1% of the variance. Participants with excellent OHRQoL but low POH constituted the largest group in this dimension. Similar findings were reported by Espinoza et al., ^30^ who identified a negative association between oral health and perceived disability in Peruvian populations.

Overall, these results highlight that while some dimensions, such as functional limitation, show minimal association with oral health perception, others—particularly psychological discomfort, physical disability, and social disability—present stronger relationships. This evidence underscores the need for **occupational health strategies addressing the physical and psychosocial determinants of oral health perception**, ensuring early detection, preventive education, and comprehensive care approaches for healthcare personnel.

### Study limitations

The discussion of this study is framed within certain methodological limitations related to its design and sample size. Since the research was conducted in a single Level II-1 hospital in northern Peru, the findings cannot be generalized to larger populations or other geographic settings due to the contextual specificities of the sample and the institution. Similarly, the limited sample size (n = 72) restricts generalization to other healthcare professionals, and the non-probabilistic convenience sampling introduces a potential selection bias.

Moreover, instruments specifically validated for measuring oral health–related quality of life (OHRQoL) and oral health perception (POH) in healthcare personnel were used. While these tools improve validity and comparability, they present inherent limitations due to their self-reported nature, making them susceptible to subjectivity and social desirability biases; objective clinical measures that could have allowed triangulation of self-reported data were not included.

Another key limitation lies in the cross-sectional design of the study. Because data were collected at a single point in time, it is not possible to establish causal relationships between OHRQoL and POH or to assess changes over time. Nevertheless, the findings provide valuable inputs for the design of longitudinal studies and repeated-measures research aimed at exploring the evolution of these variables in different healthcare settings.

Finally, although the sample was adequate for the proposed scope, expanding the research to include other hospitals in different regions or countries (e.g., Spain or other Latin American contexts) would strengthen external validity and help identify consistent patterns or divergences across comparable healthcare systems.

### Study implications

The findings highlight the importance of oral health as a central component of the overall well-being of hospital staff. The existence of a statistically significant association between oral health–related quality of life (OHRQoL) and oral health perception (POH), together with the contribution of OHIP-14 domains such as psychological discomfort, physical disability, and social disability, underscores the need for integrated interventions addressing both the clinical dimension of oral health and the psychosocial factors determining staff well-being and, consequently, the quality of patient care.

While these results can guide the design of strategies in similar settings, their implications should be interpreted cautiously and in light of institutional and local specificities. In summary, **multidimensional and collaborative approaches**—including continuous education, periodic screening, timely access to dental care, psychosocial support, and ongoing monitoring of OHRQoL/POH—are advocated to strengthen staff well-being and contribute to sustainable institutional development.

## Conclusion

The study demonstrates a statistically significant association between **oral health–related quality of life (OHRQoL)** and **oral health perception (POH)** among healthcare professionals at a Level II-1 hospital in northern Peru. Spearman’s correlation was ρ = 0.391; p = 0.001, and the ordinal logistic regression model yielded a Nagelkerke pseudo R = 0.198, indicating a low but consistent association; higher levels of OHRQoL were associated with a more favorable POH.

Moreover, significant associations were confirmed for specific OHIP-14 dimensions: **physical disability** (ρ = 0.319; p = 0.006; pseudo R ^2^ = 0.167), **psychological disability** (ρ = 0.232; p = 0.050; pseudo R ^2^ = 0.167), **social disability** (ρ = 0.242; p = 0.040; pseudo R ^2^ = 0.124), and **handicap** (ρ = 0.298; p = 0.011; pseudo R ^2^ = 0.131).

These findings suggest that strengthening factors that improve OHRQoL—through **continuous education, periodic screening, timely access to dental care, and psychosocial support**—could lead to more favorable oral health perceptions among hospital staff. The evidence provides contextual references for similar institutional settings, recognizing the influence of sociocultural and institutional variables on these outcomes.

### Recommendations

**Develop continuing education programs:** Design and implement educational strategies focused on oral health for hospital staff. These programs should emphasize the importance of oral hygiene, the prevention of oral diseases, and their relationship with oral health–related quality of life (OHRQoL) and professional performance. Integration into existing occupational wellness programs is recommended to ensure sustainability and institutional support.

**Improve access to dental services:** Establish hospital-based dental services with flexible schedules adapted to the work shifts of healthcare personnel. Facilitating timely preventive care and treatment may contribute to improving oral health perception and outcomes.

**Monitor oral health and quality of life in the workplace:** Incorporate validated instruments such as the OHIP-14 and HU-DBI into the periodic evaluations of hospital staff. This approach would enable the systematic assessment of the impact of oral health interventions while supporting a comprehensive strategy to promote occupational well-being.

## Data availability

### Underlying data

Regarding the availability of the data used, these are publicly accessible on the Zenodo platform under the terms of the Creative Commons Attribution 4.0 International License (CC-BY 4.0).

The main database can be accessed at: https://doi.org/10.5281/zenodo.14847738.

This Excel (.xls) file contains anonymized responses to the Oral Health–Related Quality of Life (OHRQoL) and Oral Health Perception (POH) questionnaires administered to hospital staff.

The complementary methodological appendix, which includes the full instruments (OHIP-14 and HU-DBI in Spanish), as well as the expert judgment validation matrix and the reliability levels obtained, is available at: https://doi.org/10.5281/zenodo.15236712. ^34^

These files ensure the transparency and reproducibility of the data collection process.
