# Association between perceived oral health and oral health–related quality of life among hospital staff

**DOI:** 10.12688/f1000research.161146.6

**Published:** 2026-01-28

**Authors:** Miryam Lora Loza, Sheyla del Pilar Alvarado-Romero, Katia Ninozca Flores Ledesma, Nancy Cuenca Robles, David Rene Rodríguez Díaz

**Affiliations:** 1 Graduate School, César Vallejo University, Lima, 15311, Peru; 2 School of Human Medicine, Private University of the North, Trujillo, Lima Norte, 13001, Peru

**Keywords:** Quality of life, Perception, Oral health, Correlation, Hospital, Functional limitation.

## Abstract

**Background:**

Oral health is closely linked to well-being at work; however, evidence in hospital personnel remains limited.

**Methods:**

Cross-sectional study in a Level II-1 hospital (n = 72). Oral-health–related quality of life (OHRQoL) was measured with OHIP-14 and perceived oral health (POH) with a modified HU-DBI. Bivariate associations were estimated with Spearman’s ρ and 95% confidence intervals; domain-level relationships were examined with proportional-odds ordinal logistic regression (Nagelkerke’s pseudo-R ^2^).

**Results:**

OHRQoL was distributed as 38.9% Excellent, 26.4% Fair and 34.7% Poor; POH concentrated in the Low level (52.8%), followed by Excellent (29.2%) and Fair (18.1%). POH correlated positively with OHRQoL (ρ = 0.391; 95% CI 0.18–0.57; p = 0.001). Domain-level analyses showed the strongest links for psychological discomfort (ρ = 0.421; p < 0.001; pseudo-R ^2^ = 0.111; p = 0.027) and physical disability (ρ = 0.319; p = 0.006; pseudo-R ^2^ = 0.167; p = 0.004); social disability (ρ = 0.242; p = 0.040; pseudo-R ^2^ = 0.124; p = 0.017) and handicap (ρ = 0.298; p = 0.011; pseudo-R ^2^ = 0.131; p = 0.013) were smaller but significant, whereas functional limitation was non-significant (ρ = 0.096; p = 0.424; pseudo-R ^2^ = 0.014; p = 0.6).

**Conclusions:**

Better perceived oral health is significantly associated with higher oral-health-related quality of life among hospital staff. Consequently, targeted workplace strategies, including education for self-care, pain management, and functional support, could enhance oral well-being. Moreover, open instruments and pilot reliability outputs are available to ensure transparency and reproducibility.

**Abbreviations**
CI: Confidence interval
FDI: FDI World Dental Federation
HU-DBI
: Hiroshima University–Dental Behavioral Inventory
OHIP-14: Oral Health Impact Profile-14
OHRQoL: Oral health-related quality of life
POH: Perceived oral health
WHO: World Health Organization

## Introduction

Oral health is an essential component of overall well-being and is closely associated with oral health–related quality of life (OHRQoL) through the ability to perform basic functions such as communicating, eating properly, and maintaining satisfactory social relationships. ^1^ Oral diseases affect not only oral functions but also psychological, social, and economic outcomes, causing discomfort, pain, and loss of self-esteem, and shaping the subjective perception of oral well-being. ^2, 3^ The association between oral health and OHRQoL underscores its contribution to Sustainable Development Goal 3, in which oral health is a fundamental component.

Within this framework, international organizations such as the FDI World Dental Federation and the World Health Organization (WHO) consider oral health a global priority because of its clear relationship with OHRQoL. WHO conceptualizes oral health as a comprehensive state that includes physical, mental, and social well-being in relation to the functionality and condition of the oral cavity, rather than the mere absence of disease, ^4^ while FDI Vision 2030 advances an integrative perspective that links oral well-being with sustainable public health policies. ^1, 5^ Taken together, these definitions provide the policy lens through which the global burden of oral diseases must be interpreted.

Accordingly, oral diseases constitute a major global public health problem that affects approximately 3.5 billion people, with a higher burden in low- and middle-income countries where about 75% of cases are concentrated. ^4^ Untreated dental caries is the most prevalent condition, reflecting deep inequities in access to preventive services and basic treatments. ^4, 6, 7^ In Latin America, periodontal diseases represent a widespread burden that significantly impairs OHRQoL. In Peru, the magnitude of the problem is exacerbated by the low priority assigned to oral health within health agendas, limited public investment, and an elevated oral cancer burden, which together indicate persistent gaps in prevention and early detection. ^7^ In response, global initiatives—most notably FDI Vision 2030 and the Seventy-fourth World Health Assembly resolution on oral health—emphasize integrating oral health into Universal Health Coverage and aligning actions with the noncommunicable disease agenda. ^5, 8^

Consistent with that agenda, the WHO Global Oral Health Status Report (2022) calls for reorienting public policies to prioritize oral-health promotion and the production of scientific knowledge through national plans aligned with WHO’s strategic vision. ^4, 7^ In line with this approach, WHO and FDI advocate intersectoral collaboration within broader development agendas. ^4, 5, 9^ Within healthcare systems, perceived oral health (POH) is a measurable construct that correlates with OHRQoL ^10, 11^ and can be shaped by contextual factors such as limited access to dental services and workload. ^12^ Because negative POH undermines self-esteem and interpersonal relationships, monitoring staff well-being through OHRQoL metrics is warranted. ^9, 13^

**Figure 1.**
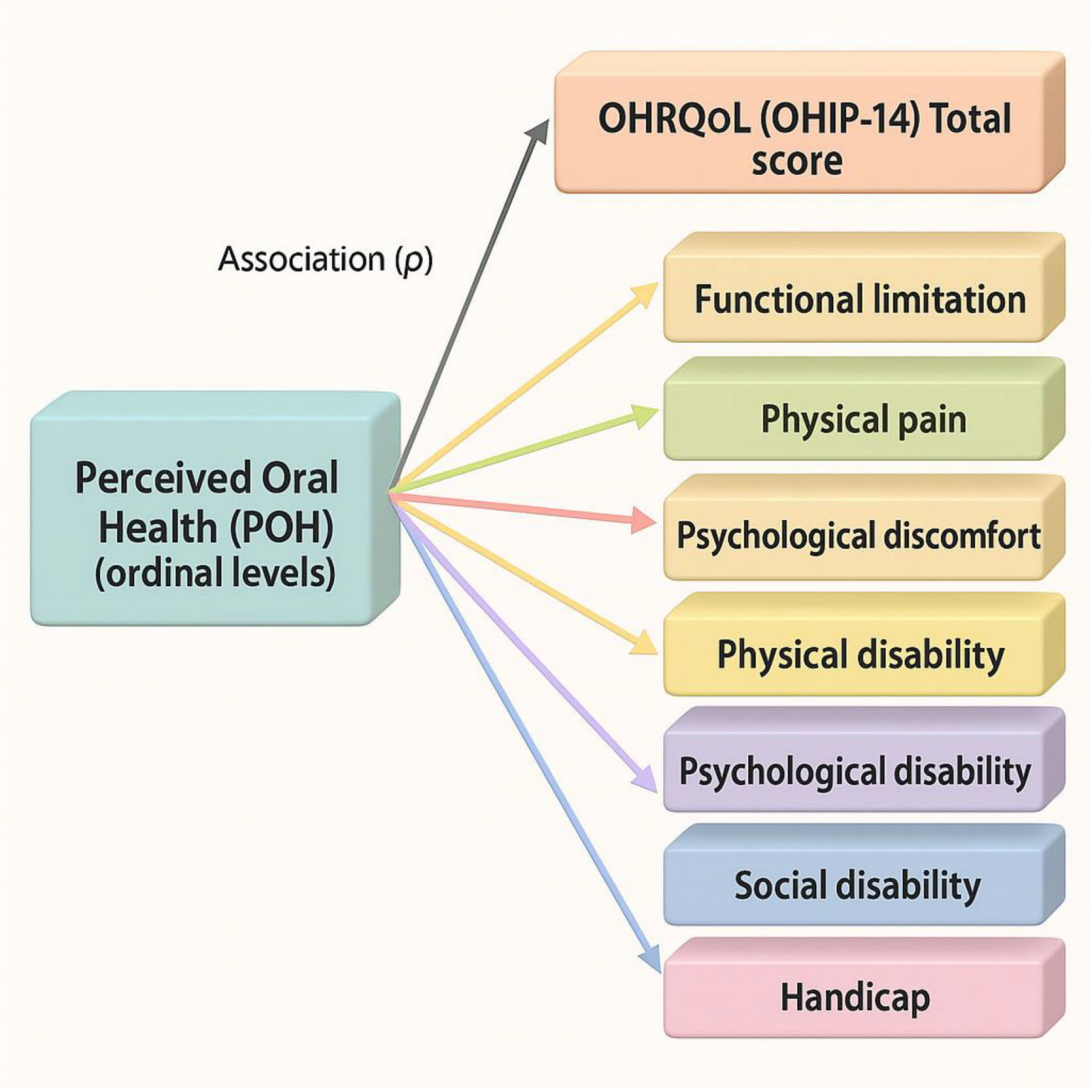
Conceptual association model.

On this basis, and given the persistent regional inequities, Peruvian and Latin American evidence highlights enduring gaps in dental access and occupational constraints among healthcare workers, which in turn makes hospital-based monitoring of OHRQoL and POH particularly relevant. To ground this need empirically, the present study examines how perceived oral health relates to oral health related quality of life, among staff in a level II-1 hospital in northern Peru, a workforce that remains underrepresented in the regional literature. Figure 1 summarizes the conceptual associations tested. Accordingly, the aim of this study was to examine the association between OHRQoL and perceived oral health among staff at a level II-1 hospital in northern Peru, considering domain-level dimensions and their associations, in order to inform strategies that promote staff oral well-being and support comprehensive, patient-centered care consistent with quality criteria. ^14, 15^

**Conceptual diagram showing associations from perceived oral health (ordinal levels) to OHIP-14 total score and its seven domains.**

Diagram showing the hypothesized associations between perceived oral health (POH) and oral-health–related quality of life (OHRQoL, OHIP-14) domains. Associations were assessed using Spearman’s ρ (bivariate) and proportional-odds ordinal logistic regression for domain-level analyses. Higher POH levels are expected to align with better OHRQoL (lower OHIP-14 burden).

## Methods

**Figure 2.**
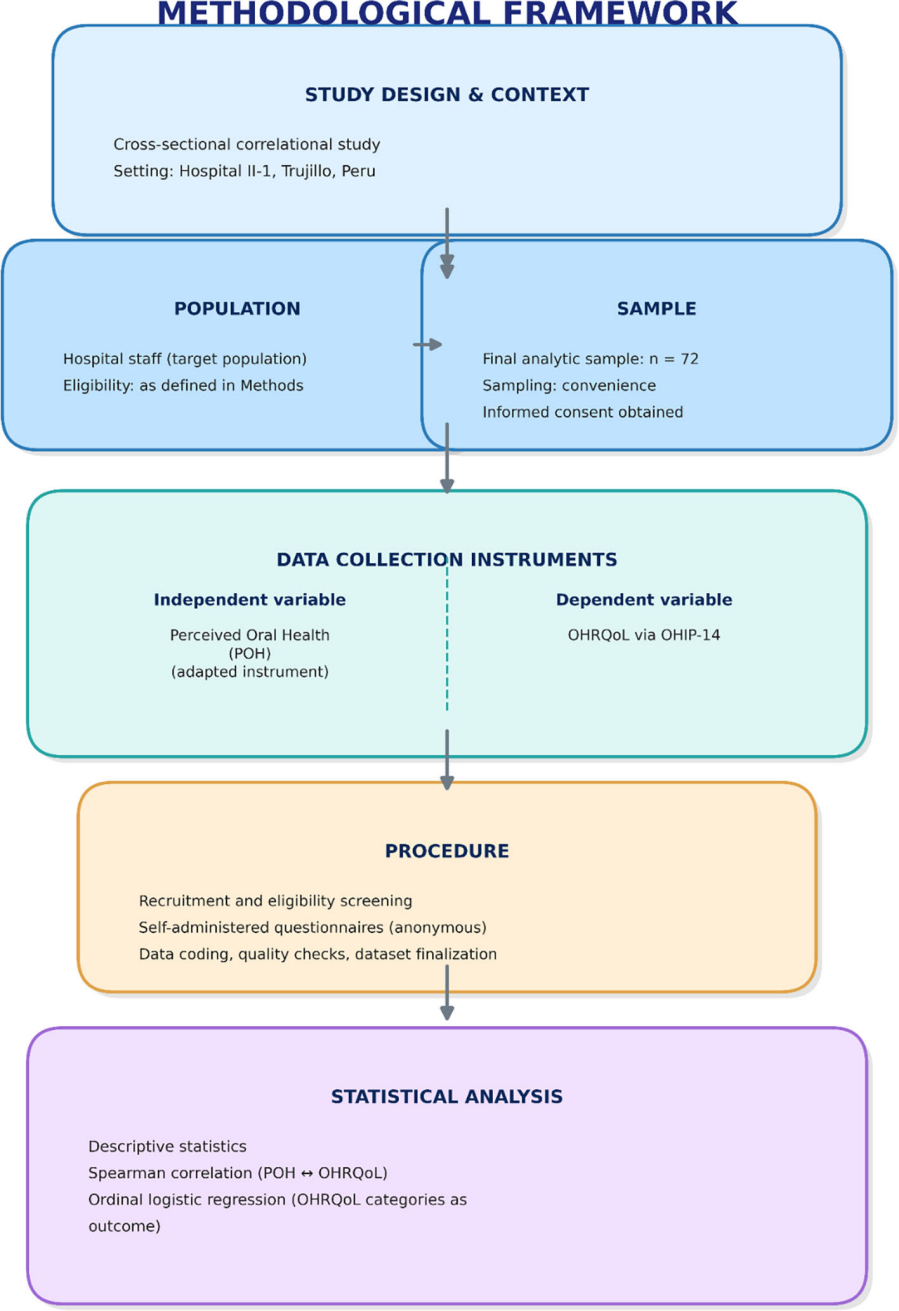
Research methodology flowchart (methodological framework). Summary of the study workflow from design and setting to sampling, instruments, procedures, and statistical analyses (image format).

To facilitate replication, the methodological workflow of the study is summarized in Figure 2 (research flowchart, image format). The figure outlines the study design and setting, population and sampling, instruments, procedures, and statistical analyses.

Further methodological details are described in the subsections below.

### Research type and design

This applied study addressed a concrete problem in a hospital setting and adopted a correlational scope to estimate the strength and direction of the association between variables. A non-experimental, cross-sectional design was used; data were collected at a single time point without manipulating variables, preserving the natural context of observation. ^16, 17^

### Population and sample

The sampling frame comprised 80 professional and technical staff at a Level II-1 hospital in northern Peru (21 physicians, 10 obstetricians, 1 dentist, 1 pharmaceutical chemist, 17 nurses, 2 psychologists, 3 biologists, 5 microbiologists, 2 medical technologists, 13 nursing technicians, 4 pharmacy technicians, and 1 laboratory technician). Eligibility criteria included being designated or contracted under the Administrative Services Contract, having at least six months of tenure, agreeing to complete both questionnaires, and signing informed consent; conversely, workers with less than six months of tenure, those on vacation or medical leave, those in outsourced modalities, those currently in SERUMS, or those who declined participation were excluded. After applying these criteria and accounting for unavailability or non-consent during data collection, 72 individuals completed both instruments and were included in the analysis, which corresponds to a participation rate of 90%.

**Power and sample size.** An a priori power analysis (G*Power 3.1) for a two-tailed correlation with an expected ρ = 0.30, α = 0.05, and 1−β = 0.80 indicated a minimum of 67 participants; therefore, the achieved sample (n = 72) was adequate for the planned correlational analyses. ^18– 21^

### Variables and instruments

Two primary variables were analyzed: oral health–related quality of life (OHRQoL) and perceived oral health (POH). OHRQoL is conceptualized as the subjective appraisal of the impact that oral conditions exert on the physical, emotional, and social aspects of everyday life. For its measurement, two instruments were considered: the OHIP-14 and the GOHAI. The OHIP-14 was selected because it is a validated short form that covers seven functional and psychosocial domains and has demonstrated broad applicability in Spanish-speaking contexts. Although the GOHAI is a useful tool, its predominant emphasis on functional limitation makes it more suitable for older adult populations. Given the heterogeneous composition of hospital personnel, the OHIP-14 provided broader construct coverage and greater comparability for interpreting results. ^22– 24^

**Perceived oral health (POH).** POH denotes the subjective evaluation of one’s oral status and its links with daily functioning. For this construct, the modified Hiroshima University–Dental Behavioral Inventory (HU-DBI) was applied, originally developed by Kawamura and subsequently used and adapted in Peru. The instrument comprises 20 dichotomous items (Yes = 1; No = 0) organized across knowledge, behavior, and attitude, and it serves as an indicator of awareness, disposition, and practice regarding one’s oral health. ^25, 26^

### Data collection technique

A structured survey was used to collect standardized data from professional and technical staff via self-administered questionnaires, without altering the work setting or the object of study. ^20^

### Operational details and scoring

For OHRQoL, the OHIP-14—original by Slade and Spencer (1994), adapted by Espinoza (2017) and validated/published by Espinoza et al. (2022) ^22– 24^—used a 5-point Likert scale (0 = never to 4 = very frequently). For interpretability, total scores followed Espinoza’s criteria: Excellent (0–2), Fair (3–9), and Poor (≥10). For domain-level descriptions (two items per domain; range 0–8), thresholds proportional to the total-scale cutoffs were applied: Excellent (0–1), Fair (2–3), Poor (≥4).

For POH, the modified HU-DBI comprises 20 dichotomous items and was summarized as: Poor (0–9), Fair (10), Excellent (11–20). ^25, 26^

### Validity and reliability

Content validity for both instruments was established by a five-member expert panel who evaluated internal consistency, conceptual clarity, thematic relevance, and informational sufficiency; the aggregated Aiken’s V was 1.00, as documented in the methodological appendix. ^27^ Pilot testing showed acceptable internal consistency: Cronbach’s α = 0.847 for the OHIP-14 and α = 0.804 for the modified HU-DBI, with full scoring keys and reliability outputs available in the same appendix. ^27^ Instrument provenance and structure follow the original OHIP-14 development and the HU-DBI framework. ^22, 25^

### Transparency and materials

For methodological transparency and replication, all materials (OHIP-14, modified HU-DBI, expert-review matrix, and pilot reliability outputs) are openly available on Zenodo. ^27, 28^

### Procedure

The study commenced with a formal cover letter from the Graduate School, Universidad César Vallejo, to the administrative department of the La Libertad Level II-1 Hospital requesting authorization to administer the instruments. After approval, on-site sessions were scheduled. Investigators explained objectives, procedures, confidentiality safeguards, and anonymity, and all participants provided written informed consent. Questionnaires were self-administered in discrete areas within workspaces and required approximately eight minutes per participant, minimizing distractions and contextual bias.

### Data analysis

Analyses drew on the prior application of the HU-DBI in Peruvian settings ^26^ and used the anonymized dataset and methodological appendix hosted on Zenodo. ^27, 28^ Computations were performed in IBM SPSS Statistics v25 and R. ^29, 30^ Descriptive statistics summarized continuous variables as mean and standard deviation or median and interquartile range, as appropriate. Distributional assumptions were examined with the Kolmogorov–Smirnov test; because several variables deviated from normality (p < 0.05) and POH is ordinal, nonparametric methods were applied. Bivariate associations between OHRQoL and POH were quantified with Spearman’s ρ and 95% confidence intervals. In addition, proportional odds ordinal logistic regression models were fitted to examine the relationship between OHIP-14 domains and POH levels. Results are reported as odds ratios with 95% confidence intervals and Nagelkerke’s pseudo-R ^2^. The significance level was set at α = 0.05. These analytical choices are consistent with current recommendations for ordinal, non-normally distributed data. ^31– 33^ The conceptual associations tested in this study are depicted in Figure 1.

### Ethics

The study protocol, entitled “Quality of life and perception of oral health of the staff of a Level II-1 hospital in La Libertad, 2024,” received a favorable opinion from the Research Ethics Committee of the Master’s Program in Health Services Management, Universidad César Vallejo (Report No. 00298-2024/CEI-PMGSS; 30 January 2025). The committee authorized implementation in accordance with institutional regulations and international ethical standards, including CIOMS (2016), ^34^ the Belmont Report (1979), ^35^ and the Declaration of Helsinki (WMA, 2013). ^36^ All participants provided written informed consent before data collection; confidentiality was preserved through coded identifiers and de-identification of the dataset, participation was voluntary, and individuals could withdraw at any time without penalty. The study also complied with the university’s Research Ethics Code, ^37^ ensuring originality, transparency, and methodological rigor throughout all phases.

## Results

Seventy-two hospital staff completed the survey. OHRQoL (OHIP-14) was distributed as 38.9% Excellent, 26.4% Fair, and 34.7% Poor ( Table 2). Perceived oral health (POH) concentrated at the Low level (52.8%), followed by Excellent (29.2%) and Fair (18.1%) ( Table 3).

**Bivariate association.** Perceived oral health correlated positively with OHRQoL (Spearman’s ρ = 0.391, 95% CI 0.18–0.57, p = 0.001). Given the ordinal scales and non-normality, an ordinal logistic model was additionally fitted, yielding Nagelkerke’s pseudo-R ^2^ = 0.198, indicating modest model fit consistent with a monotonic relationship between POH and OHRQoL (p = 0.001). Full cross-tabulation and coefficients appear in Table 1.

**Table 1.** Relationship between oral health–related quality of life (OHRQoL) and perceived oral health (POH) among hospital staff at a level II-1 Hospital in Northern Peru, 2024.

Oral Health–related Quality of Life (OHRQoL)	Oral Health Perception (POH)						Total	
	Low		Fair		Excellent			
	n	%	n	%	n	%	n	%
**Excellent**	21	29.20	6	8.30	1	1.40	28	38.90
**Fair**	8	11.10	1	1.40	10	13.90	19	26.40
**Poor**	9	12.50	6	8.30	10	13.90	25	34.70
**Total**	38	52.80	13	18.10	21	29.20	72	100.00

Spearman’s ρ	p	Nagelkerke pseudo-R ^2^	p
0.391	0.001	0.198	0.001

Note: Percentages are computed over the total sample (n = 72). The bottom panel summarizes the bivariate association (Spearman’s ρ, p) and the ordinal model (Nagelkerke’s pseudo-R ^2^, p).

**Domain-level analyses.** Associations between POH and OHIP-14 domains were examined with proportional-odds ordinal logistic regression and Spearman’s ρ ( Table 4). The strongest links were observed for psychological discomfort (ρ = 0.421; p < 0.001; pseudo-R ^2^ = 0.111; p = 0.027) and physical disability (ρ = 0.319; p = 0.006; pseudo-R ^2^ = 0.167; p = 0.004). Social disability (ρ = 0.242; p = 0.040; pseudo-R ^2^ = 0.124; p = 0.017) and handicap (ρ = 0.298; p = 0.011; pseudo-R ^2^ = 0.131; p = 0.013) were also significant. Functional limitation was weak and non-significant (ρ = 0.096; p = 0.424; pseudo-R ^2^ = 0.014; p = 0.649).

Synthesis. Overall, better POH aligned with better OHRQoL (lower OHIP-14 burden), with the largest domain-level contributions in psychosocial (psychological discomfort) and functional (physical disability) impacts. See Figure 1 and Tables 1– 4 for numeric details.

Table 2 summarizes OHRQoL levels and domain profiles. Overall, OHRQoL (OHIP-14) was distributed as 38.90% Excellent, 26.40% Fair, and 34.70% Poor. By domain, the highest proportions in the Excellent level were observed for social disability (76.40%) and handicap (83.30%), whereas functional limitation concentrated 34.70% in the Poor level. This pattern indicates heterogeneous domain impacts: social participation and global functioning appear comparatively preserved, while functional limitation is the most affected dimension, consistent with the OHIP-14 framework and its cross-cultural validations. ^22– 24^

**Table 2.** Levels of oral health–related quality of life (OHRQoL) and OHIP-14 domain classifications among hospital staff at a level II-1 Hospital in Northern Peru, 2024.

OHRQoL level	Dimensions of Oral Health–Related Quality of Life (OHRQoL)													
	Functional limitation		Physical pain		Psychological discomfort		Psychological disability		Physical disability		Social disability		Handicap	
Levels	n	%	n	%	n	%	n	%	n	%	n	%	n	%
**Excellent**	28	38.90	32	44.40	38	52.80	39	54.20	50	69.40	55	76.40	60	83.30
**Fair**	19	26.40	28	38.90	23	31.90	22	30.60	18	25.00	14	19.40	10	13.90
**Poor**	25	34.70	12	16.70	11	15.30	11	15.30	4	5.60	3	4.20	2	2.80
**Total**	72	100.00	72	100.00	72	100.00	72	100.00	72	100.00	72	100.00	72	100.00

Note: Percentages are computed column-wise within each OHIP-14 domain. Overall OHRQoL (OHIP-14): Excellent (0–2), Fair (3–9), Poor (≥10). ^22– 24^

Table 3 summarizes the distribution of perceived oral health (POH) and its dimensions. Overall POH concentrated in the Low level at 52.80%, followed by Excellent at 29.20% and Fair at 18.00%. At the dimensional level, Knowledge Perception was entirely classified as Low at 100.00%, which reveals a critical knowledge gap among staff. Attitude Perception split between Low at 44.40% and Fair at 43.10%, indicating polarization between incipient and moderate attitudes. Behavior Perception peaked at the Fair level at 62.50%, suggesting acceptable but suboptimal habitual practices. Taken together, this configuration of low knowledge, intermediate attitudes, and mostly fair behaviors points to a knowledge–attitude–behavior gap that likely reflects unstructured learning processes and operational barriers. A stepwise intervention is therefore indicated: first strengthen knowledge through targeted training, then reframe attitudes through motivational messaging and clear service standards, and finally reinforce behaviors through reminders, access facilitation, and follow-up. ^25, 26^

**Table 3.** Levels of perceived oral health (POH) and POH dimensions among hospital staff at a Level II-1 Hospital in northern Peru, 2024.

Oral Health Perception (POH)			Dimensions (POH)					
			Knowledge Perception		Attitude Perception		Behavior Perception	
Levels	n	%	n	%	n	%	n	%
**Low**	38	52.80	72	100.00	32	44.40	15	20.80
**Fair**	13	18.00	0	0.00	31	43.10	45	62.50
**Excellent**	21	29.20	0	0.00	9	12.50	12	16.70
**Total**	72	100.00	72	100.00	72	100.00	72	100.00

Note: Percentages are computed column-wise within each construct (global POH and each POH dimension). POH categories for the modified HU-DBI are: Low (0–9), Fair (10), Excellent (11–20). ^25, 26^

Abbreviations: POH, perceived oral health; HU-DBI, Hiroshima University–Dental Behavioral Inventory.

This pattern of markedly low knowledge, intermediate attitudes, and mostly regular behaviors indicates a knowledge–attitude–behavior gap associated with unstructured learning processes and operational barriers (e.g., service access times, organizational culture). Overall, the findings highlight the need for a stepwise intervention approach: strengthening knowledge (targeted training), reframing attitudes (motivational messaging and service standards), and reinforcing behaviors (reminders, access facilities, follow-up).

Table 4 Domain-level analyses. Table 4 summarizes the associations between OHIP-14 domains and perceived oral health (POH) using Spearman’s ρ and ordinal logistic regression. Psychological discomfort showed the strongest link with POH (ρ = 0.421; p < 0.001) and contributed significantly to the model (Nagelkerke’s pseudo-R ^2^ = 0.111; p = 0.027). Physical disability was also significant (ρ = 0.319; p = 0.006; pseudo-R ^2^ = 0.167; p = 0.004), as was physical pain (ρ = 0.266; p = 0.024; pseudo-R ^2^ = 0.093; p = 0.049). Social disability (ρ = 0.242; p = 0.040; pseudo-R ^2^ = 0.124; p = 0.017) and handicap (ρ = 0.298; p = 0.011; pseudo-R ^2^ = 0.131; p = 0.013) displayed smaller but significant associations. By contrast, functional limitation was weak and non-significant (ρ = 0.096; p = 0.424; pseudo-R ^2^ = 0.014; p = 0.649), indicating minimal association and poor model fit for that domain. Overall, OHRQoL and POH were positively related (ρ = 0.391; 95% CI 0.18–0.57; p = 0.001); the ordinal model yielded Nagelkerke’s pseudo-R ^2^ = 0.198, indicating modest model fit consistent with a monotonic POH–OHRQoL relationship.

**Table 4.** Associations between OHIP-14 domains and perceived oral health (POH) among hospital staff at a Level II-1 Hospital in Northern Peru, 2024.

Functional limitation	Oral Health perception (POH)								Inferential analysis			
	Low		Fair		Excellent		Total		Spearman’s ρ	p-value	Nagelkerke’s pseudo R ^2^	*p*
	n	%	n	%	n	%	n	%				
Excellent	18	25.00	7	9.70	7	9.70	32	44.40	0.096	0.424	0.014	0.649
Fair	15	20.80	3	4.20	10	13.90	28	38.90				
Poor	5	6.90	3	4.20	4	5.60	12	16.70				
**Total**	38	52.80	13	18.10	21	29.20	72	100.00				

Physical Pain	Oral Health perception (POH)											
	Low		Fair		Excellent		Total					
	n	%	n	%	n	%	n	%				
Excellent	24	33.30	7	9.70	7	9.70	38	52.80	0.266	0.024	0.093	0.049
Fair	12	16.70	2	2.80	9	12.50	23	31.90				
Poor	2	2.80	4	5.60	5	6.90	11	15.30				
**Total**	38	52.80	13	18.10	21	29.20	72	100.00				

Psychological discomfort	Oral Health perception (POH)											
	Low		Fair		Excellent		Total					
	n	%	n	%	n	%	n	%				
Excellent	26	36.10	6	8.30	7	9.70	39	54.20	0.421	< 0.001	0.111	0.027
Fair	8	11.10	5	6.90	9	12.50	22	30.60				
Poor	4	5.60	2	2.80	5	6.90	11	15.30				
**Total**	38	52.80	13	18.10	21	29.20	72	100.00				

Physical Disability	Oral Health perception (POH)											
	Low		Fair		Excellent		Total					
	n	%	n	%	n	%	n	%				
Excellent	30	41.70	8	11.10	12	16.70	50	69.40	0.319	0.006	0.167	0.004
Fair	8	11.10	5	6.90	5	6.90	18	25.00				
Poor	0	0.00	0	0.00	4	5.60	4	5.60				
**Total**	38	52.80	13	18.10	21	29.20	72	100.00				

Psychological disability	Oral Health perception (POH)											
	Low		Fair		Excellent		Total					
	n	%	n	%	n	%	n	%				
Excellent	30	41.70	8	11.10	12	16.70	50	69.40	0.232	0.050	0.167	0.004
Fair	8	11.10	5	6.90	5	6.90	18	25.00				
Poor	0	0.00	0	0.00	4	5.60	4	5.60				
**Total**	38	52.80	13	18.10	21	29.20	72	100.00				

Social disability	Oral Health perception (POH)											
	Low		Fair		Excellent		Total					
	n	%	n	%	n	%	n	%				
Excellent	31	43.10	11	15.30	13	18.10	55	76.40	0.242	0.040	0.124	0.017
Fair	7	9.70	2	2.80	5	6.90	14	19.40				
Poor	0	0.00	0	0.00	3	4.20	3	4.20				
**Total**	38	52.80	13	18.10	21	29.20	72	100.00				

General disability (handicap)	Oral Health perception (POH)											
	Low		Fair		Excellent		Total					
	n	%	n	%	n	%	n	%				
Excellent	36	50.00	9	12.50	15	20.80	60	83.30	0.298	0.011	0.131	0.013
Fair	2	2.80	4	5.60	4	5.60	10	13.90				
Poor	0	0.00	0	0.00	2	2.80	2	2.80				
**Total**	38	52.80	13	18.10	21	29.20	72	100.00				

Note: For each OHIP-14 domain, Spearman’s ρ quantifies monotonic ordinal associations; an ordinal (proportional odds) logistic regression was also fitted. Values shown are Nagelkerke’s pseudo-R ^2^ and two-sided p-values. These analytical choices follow current recommendations for ordinal, non-normally distributed data. ^31– 33^

## Discussion

Oral health–related quality of life (OHRQoL) and perceived oral health (POH) are essential determinants of the overall well-being of healthcare workers. In this regard, the study analyzed their relationship to identify priority areas for oral-health interventions. Furthermore, the discussion interprets the main findings in light of the general objective, which was to examine the association between POH and OHRQoL, and situates them within current epidemiological and health-policy frameworks, while acknowledging the inherent limitations of a cross-sectional design.

In Table 1, the distribution of categories showed heterogeneity, as OHRQoL clustered at Excellent (38.9%) and Poor (34.7%), whereas POH predominantly fell in the Low category (52.8%); moreover, the bivariate test indicated a positive, monotonic association between POH and OHRQoL (Spearman’s ρ = 0.391; 95% CI 0.18–0.57; p = 0.001). In addition, the ordinal model supported this pattern (Nagelkerke’s pseudo-R ^2^ = 0.198), which reflects convergence between subjective appraisal and quality-of-life impact without establishing causation. ^31– 33^ These results align with Díaz-Reissner et al., ^11^ who reported that sociodemographic and clinical determinants shape OHRQoL and, reciprocally, self-perceived oral health. By contrast, Espinoza et al. ^24^ documented a higher prevalence of excellent OHRQoL (66.8%), a difference plausibly explained by contextual factors such as infrastructure, access to dental services, and occupational-health policies. Consistent with the World Health Organization’s perspective on the interdependence of oral health, quality of life, and subjective health perceptions, the present findings underscore the value of monitoring both constructs in workforce settings to inform targeted, feasible interventions. ^6^

Regarding OHRQoL profiles ( Table 2), the distribution across Excellent (38.9%), Fair (26.4%), and Poor (34.7%) coexisted with heterogeneous domain patterns: social disability (76.4%) and handicap (83.3%) concentrated in the Excellent level, whereas functional limitation clustered in the Poor level (34.7%). This divergence between global status and domain burdens accords with the OHIP-14 framework, which anticipates that specific functional complaints (for example, chewing and speech) can persist even when social participation and overall appraisal remain favorable. ^22– 24^ In working populations, social roles may be buffered by adaptive behaviors and institutional support, while basic functions remain more sensitive to subclinical oral problems; this interpretation aligns with broader evidence that oral diseases influence multiple life domains with uneven intensity. ^4, 7^ The pattern therefore justifies domain-targeted measures such as functional rehabilitation and pain management, implemented alongside general health-promotion strategies.

Turning to POH ( Table 3), Low POH predominated (52.8%), followed by Excellent (29.2%) and Fair (18.0%), together with a pronounced knowledge, attitude, and behavior gap (knowledge 100% Low; attitude split between Low and Fair; behavior mainly Fair). In hospital environments, the absence of protected training time and shift misalignments can hinder routine dental care and access to reliable information; under such conditions, knowledge acquisition tends to improve more slowly, whereas behaviors may show intermediate gains when logistical barriers are partially alleviated. Evidence from HU-DBI applications supports that structured education preferentially raises knowledge and attitudes, with behaviors consolidating when organizational access improves. ^25, 26^ Consistent policy guidance emphasizes institutionalizing continuous education and integrating oral health into broader well-being and universal health-coverage agendas to correct these gaps. ^2, 4, 6, 9, 27^

In addition, Table 3 highlights that POH concentrated in Low (52.8%), followed by Excellent (29.2%) and Fair (18.0%); the fact that knowledge was entirely Low signals a critical deficit that is plausible in settings without permanent professional development in oral health. Organizational constraints and limited access to information can depress knowledge and delay attitudinal change, whereas behaviors may improve modestly when access barriers are addressed. ^2, 4, 6, 9, 27^ Notably, López García reported high POH knowledge (81.5%) in another Peruvian hospital during the COVID-19 period, which suggests setting-specific differences that merit local needs assessments. ^38^

Addressing the dimension-level analyses ( Table 4), the strongest associations with perceived oral health (POH) were observed for psychological discomfort (ρ = 0.421; p < 0.001; Nagelkerke pseudo-R ^2^ = 0.111; p = 0.027) and physical disability (ρ = 0.319; p = 0.006; pseudo-R ^2^ = 0.167; p = 0.004). Social disability (ρ = 0.242; p = 0.040; pseudo-R ^2^ = 0.124; p = 0.017) and handicap (ρ = 0.298; p = 0.011; pseudo-R ^2^ = 0.131; p = 0.013) were also significant, whereas functional limitation showed a weak, non-significant relationship (ρ = 0.096; p = 0.424; pseudo-R ^2^ = 0.014; p = 0.649). Taken together with the global models, these gradients indicate that psychosocial strain and activity restrictions map more closely onto self-ratings of oral health than isolated functional complaints in working adults. ^31– 33^

For functional limitation, the association with POH was weak and non-significant (ρ = 0.096; p = 0.424; pseudo-R ^2^ = 0.014; p = 0.649). This pattern is consistent with the OHIP-14 construct, in which difficulties such as chewing or pronunciation can be offset in working adults by coping strategies and role adaptation, which reduces their weight in global self-ratings of oral health. ^22– 24^ Stronger links between functional symptoms and self-perception are typically observed in populations with higher vulnerability, including older adults and those with substantial tooth loss, which helps explain discrepancies with some community-based reports. ^10, 24^

For physical pain, the correlation with POH was positive, small, and statistically significant (ρ = 0.266; p < 0.05). The absence of pain plausibly reduces the salience of care needs, whereas its presence degrades self-assessment, which accords with clinical and population evidence showing that pain and periodontitis severity produce measurable decrements in oral health–related quality of life and shape subjective health appraisals. ^4, 7, 10^

For psychological discomfort, the strongest association was observed (ρ = 0.421; p < 0.001; pseudo-R ^2^ = 0.111; p = 0.027). This dimension aggregates worry, embarrassment, and emotional strain, which individuals tend to weight heavily when forming global judgments of oral health; the gradient is theoretically coherent with the OHIP-14 emphasis on psychosocial consequences and with population analyses that attribute a substantial share of perceived burden to psychological components rather than to isolated symptoms. ^4, 7, 22– 24^

For physical disability, the correlation was moderate and significant (ρ = 0.319; p = 0.006; pseudo-R ^2^ = 0.167; p = 0.004). Restrictions in daily activity, such as eating or extended speaking, map closely onto self-ratings of health, which explains the observed contribution; clinical studies that quantify periodontitis and tooth-loss effects report declines in functioning and quality of life with clear translation to perceived status, and the pattern here mirrors that direction with moderate magnitude in an active workforce. ^10, 22– 24^

For psychological disability, a small but significant association emerged (ρ = 0.232; p < 0.05). When psychological capacity is unimpaired, vigilance about oral self-care may be lower; conversely, perceived psychological constraints can heighten awareness of need. Prior OHIP-14 work describes this dimension as a mediator that links symptoms to global valuation, and contemporary reviews identify the mental-health sphere as a key modulator of perceived health. ^7, 22– 24^

For social disability, the association was weak yet significant (ρ = 0.242; p = 0.040; pseudo-R ^2^ = 0.124; p = 0.017). Among employed adults, social participation is often preserved despite symptoms, which tempers its influence on global perception; however, when social restrictions accumulate, self-ratings deteriorate appreciably, a behavior reported across community and clinical series and compatible with the present estimates. ^4, 7, 10^

For handicap (general disability), a small but significant association was found (ρ = 0.298; p = 0.011; pseudo-R ^2^ = 0.131; p = 0.013). Because this OHIP-14 dimension summarizes global consequences, it tracks closely with how respondents synthesize their oral condition into a single judgment, which supports its usefulness as a prioritization signal in occupational settings. ^22– 24^

Taken together, psychosocial and disability components explain POH more convincingly than isolated functional symptoms, which is consistent with international evidence on daily-life impact and with the construct validity of the OHIP-14. ^4, 7, 10, 22– 24^ Methodologically, Spearman’s correlation and proportional-odds models are appropriate for ordered, non-normal indicators and support the robustness of the reported effects. ^31– 33^ Practically, these gradients argue for a dual strategy in hospital workplaces: continuous education to raise knowledge and reshape attitudes, and service-access adaptations such as shift-aligned screening, expedited referrals, pain management, and functional rehabilitation to consolidate behaviors and relieve functional complaints. Routine monitoring with validated tools, namely the OHIP-14 and the HU-DBI, can underpin iterative improvement and evaluation of policy uptake in this workforce. ^22– 26^

## Study limitations

This single-center analysis in a Level II-1 hospital constrains external validity, therefore estimates may not generalize to institutions or regions with different organizational and population profiles. The modest sample size (n = 72) and the use of non-probabilistic convenience sampling introduce risks of selection bias and imprecision. ^18^ Although instruments validated for healthcare settings were employed (OHIP-14 and a modified HU-DBI), both are self-report measures and are vulnerable to information and social-desirability biases; moreover, objective clinical indicators that would allow triangulation were not collected. ^20^ The cross-sectional design prevents causal inference and precludes assessment of within-person change over time. ^16, 17^ Future research should adopt multicenter, probabilistic sampling frames and longitudinal or repeated-measures designs, incorporating clinical examinations and administrative data to improve generalizability and enable triangulation. ^17^

## Study implications

The significant association between perceived oral health and oral health–related quality of life, together with the contributions of psychological discomfort, physical disability, and social disability, supports integrated workplace strategies that address clinical and psychosocial determinants in tandem. ^9^ In practice, context-sensitive programs should combine continuous education in oral self-care, periodic screening, and streamlined access to dental services aligned with shift schedules, complemented by psychosocial support. ^6, 9^ Routine monitoring with validated tools can guide iterative improvement and policy uptake; specifically, the OHIP-14 for oral health–related quality of life and the HU-DBI for knowledge, attitudes, and behaviors. ^22– 26^ Although these findings can inform similar settings, implementation should be tailored to local institutional conditions and paired with explicit evaluation frameworks to track impact and adjust strategies over time. ^9^

## Conclusion

This study demonstrates a statistically significant association between oral health–related quality of life (OHRQoL) and perceived oral health (POH) among healthcare personnel at a Level II-1 hospital in northern Peru. The correlation was modest but consistent (Spearman’s ρ = 0.391; p = 0.001), and the ordinal logistic model indicated explanatory contribution at the global level (Nagelkerke’s pseudo-R ^2^ = 0.198), supporting a monotonic relation between the constructs without implying causation. At the domain level, significant associations with POH were observed for psychological discomfort (ρ = 0.421; p < 0.001; pseudo-R ^2^ = 0.111), physical disability (ρ = 0.319; p = 0.006; pseudo-R ^2^ = 0.167), social disability (ρ = 0.242; p = 0.040; pseudo-R ^2^ = 0.124), and handicap (ρ = 0.298; p = 0.011; pseudo-R ^2^ = 0.131). Psychological disability showed a borderline bivariate correlation (ρ = 0.232; p = 0.050) but contributed in the ordinal model (pseudo-R ^2^ = 0.167; p = 0.004). Physical pain also displayed a smaller, yet significant, bivariate association (ρ = 0.266; p = 0.024; pseudo-R ^2^ = 0.093; p = 0.049). Taken together, these patterns suggest that psychosocial and disability-related burdens are more tightly aligned with global self-ratings of oral health than isolated functional complaints in this workforce.

## Recommendations

Develop continuing education programs: Design and implement educational strategies focused on oral health for hospital staff. These programs should emphasize the importance of oral hygiene, the prevention of oral diseases, and their relationship with oral health–related quality of life (OHRQoL) and professional performance. Integration into existing occupational wellness programs is recommended to ensure sustainability and institutional support.

Improve access to dental services: Establish hospital-based dental services with flexible schedules adapted to the work shifts of healthcare personnel. Facilitating timely preventive care and treatment may contribute to improving oral health perception and outcomes.

Monitor oral health and quality of life in the workplace: Incorporate validated instruments such as the OHIP-14 and HU-DBI into the periodic evaluations of hospital staff. This approach would enable the systematic assessment of the impact of oral health interventions while supporting a comprehensive strategy to promote occupational well-being.

## Data Availability

All underlying data are available on Zenodo under a Creative Commons Attribution 4.0 International (CC BY 4.0) licence. The main database is deposited at https://doi.org/10.5281/zenodo.14847738. ^28^ The file contains anonymized item-level responses to the Oral Health Impact Profile (OHIP-14) and the Perceived Oral Health (modified HU-DBI) questionnaires administered to hospital staff (n = 72), as well as a brief data dictionary. **Extended/methodological materials**. The complementary methodological appendix—including the full instruments in Spanish (OHIP-14 and HU-DBI), the expert-judgment validation matrix, and pilot reliability outputs—is available at https://doi.org/10.5281/zenodo.15236712. ^27^ **Notes on reuse and ethics.** Data were fully anonymized; direct identifiers were removed and indirect identifiers were minimized according to the approved protocol (see Ethics). There are no restrictions on reuse; please cite the DOIs above and this article when using these materials.
