# Supplementary material for: Association between perceived oral health and oral health–related quality of life among hospital staff
Source: F1000Res. 2026 Jan 28;14:238. Originally published 2025 Feb 26. [Version 6] doi: 10.12688/f1000research.161146.6 (PMC12815370; doi:10.12688/f1000research.161146.6)
Supplement: Peer review history

# Reviewer report: Reviewer response for version 6

**DOI:** 10.5256/f1000research.195409.r453581

**Published:** 2026-03-02

**Authors:** Fadil Abdillah Arifin

**Affiliations:** 1 Universitas Muslim Indonesia, Makassar, Indonesia

I have checked the PPT file of the flowchart that was made according to the previous instructions. The flowchart is quite good and well-structured. Thank you.

Is the work clearly and accurately presented and does it cite the current literature?

Partly

If applicable, is the statistical analysis and its interpretation appropriate?

I cannot comment. A qualified statistician is required.

Are all the source data underlying the results available to ensure full reproducibility?

Yes

Is the study design appropriate and is the work technically sound?

Partly

Are the conclusions drawn adequately supported by the results?

Yes

Are sufficient details of methods and analysis provided to allow replication by others?

Partly

Reviewer Expertise:

OHRQoL, Medical and dental education, Endodontics

I confirm that I have read this submission and believe that I have an appropriate level of expertise to confirm that it is of an acceptable scientific standard.

# Reviewer report: Reviewer response for version 5

**DOI:** 10.5256/f1000research.191197.r440649

**Published:** 2025-12-30

**Authors:** Fadil Abdillah Arifin, Nur Surya Wirawan

**Affiliations:** 1 Universitas Muslim Indonesia, Makassar, Indonesia; 2 Department Anaesthesiology and Intensive Therapy, Universitas Hasanuddin Fakultas Kedokteran (Ringgold ID: 355661), Makassar, South Sulawesi, Indonesia

1. OK

 2. OK

 3. I have not yet viewed the flowchart as I previously recommended. A flowchart is essential for readers to understand the research process and facilitate the replication of analogous research methodologies. The author has already provided clarification; however, the flowchart I am referencing is a research flowchart in image format.

 4. OK

 5. OK

 6. OK

Is the work clearly and accurately presented and does it cite the current literature?

Partly

If applicable, is the statistical analysis and its interpretation appropriate?

I cannot comment. A qualified statistician is required.

Are all the source data underlying the results available to ensure full reproducibility?

Yes

Is the study design appropriate and is the work technically sound?

Partly

Are the conclusions drawn adequately supported by the results?

Yes

Are sufficient details of methods and analysis provided to allow replication by others?

Partly

Reviewer Expertise:

OHRQoL, Medical and dental education, Endodontics

We confirm that we have read this submission and believe that we have an appropriate level of expertise to confirm that it is of an acceptable scientific standard, however we have significant reservations, as outlined above.

# Response

**Published:** 2026-01-17

**Authors:** mlora@ucv.edu.pe Lora Loza

**Affiliations:** La Libertad, Universidad Cesar Vallejo Facultad de Ciencias Medicas, Trujillo /Trujillo, Trujillo, Peru

The requested research methodology flowchart has now been provided in **image format** and uploaded as a **separate figure file** ( **Figure 2: Research methodology flowchart / methodological framework**). In addition, the Methods section explicitly cites **Figure 2** to guide readers through the study workflow (study design and setting, population and sampling, instruments, procedure, and statistical analyses). The authors believe this addition improves transparency and facilitates replication of analogous research methodologies.

# Reviewer report: Reviewer response for version 5

**DOI:** 10.5256/f1000research.191197.r440648

**Published:** 2025-12-28

**Authors:** Pankaj Dhawan

**Affiliations:** 1 SCHOOL OF DENTAL SCIENCES, Manav Rachna International Institute of Research and Studies, Faridabad, Haryana, India

The study is a competently executed cross-sectional analysis that makes a valuable contribution to the regional literature on occupational oral health. The authors successfully document a significant association between a staff member's subjective assessment of their oral health (Perceived Oral Health, POH) and their overall oral-health-related quality of life (OHRQOL). 

 This study provides compelling evidence of the link between self-perceived oral health and well-being among hospital staff, strongly supporting the need for targeted, context-specific workplace oral health promotion strategies. While the methodological limitations prevent broad generalization and causal claims, the authors have shown an excellent ability to respond to scientific peer review, leading to a much stronger and more transparent Version 5. The core finding regarding the knowledge gap is a clear mandate for immediate public health action in this setting.

https://f1000research.com/articles/14-238/v4#referee-response-428725

Is the work clearly and accurately presented and does it cite the current literature?

Yes

If applicable, is the statistical analysis and its interpretation appropriate?

Yes

Are all the source data underlying the results available to ensure full reproducibility?

Yes

Is the study design appropriate and is the work technically sound?

Partly

Are the conclusions drawn adequately supported by the results?

Yes

Are sufficient details of methods and analysis provided to allow replication by others?

Yes

Reviewer Expertise:

Education, Dentistry, Research, Oral health.

I confirm that I have read this submission and believe that I have an appropriate level of expertise to confirm that it is of an acceptable scientific standard.

# Response

**Published:** 2025-12-28

**Authors:** mlora@ucv.edu.pe Lora Loza

**Affiliations:** La Libertad, Universidad Cesar Vallejo Facultad de Ciencias Medicas, Trujillo /Trujillo, Trujillo, Peru

Thank you for reviewing Version 5 and for approving the manuscript. We appreciate your confirmation that our revisions addressed the previous comments.

# Reviewer report: Reviewer response for version 5

**DOI:** 10.5256/f1000research.191197.r440644

**Published:** 2025-12-28

**Authors:** Rasha Salah

**Affiliations:** 1 University of Baghdad, Baghdad, Iraq

the authors  have addressed my previous comments and revised the manuscript.

Is the work clearly and accurately presented and does it cite the current literature?

Yes

If applicable, is the statistical analysis and its interpretation appropriate?

Yes

Are all the source data underlying the results available to ensure full reproducibility?

Yes

Is the study design appropriate and is the work technically sound?

Partly

Are the conclusions drawn adequately supported by the results?

Partly

Are sufficient details of methods and analysis provided to allow replication by others?

Yes

Reviewer Expertise:

periodontics, implantology

I confirm that I have read this submission and believe that I have an appropriate level of expertise to confirm that it is of an acceptable scientific standard.

# Response

**Published:** 2025-12-28

**Authors:** mlora@ucv.edu.pe Lora Loza

**Affiliations:** La Libertad, Universidad Cesar Vallejo Facultad de Ciencias Medicas, Trujillo /Trujillo, Trujillo, Peru

Thank you for reviewing Version 5 and for confirming that the manuscript revisions adequately addressed all prior comments. We appreciate your approval.

# Reviewer report: Reviewer response for version 4

**DOI:** 10.5256/f1000research.188626.r428728

**Published:** 2025-11-21

**Authors:** Rasha Salah

**Affiliations:** 1 University of Baghdad, Baghdad, Iraq

there are missing detais in the methodology:

  • Who administered the questionnaires?

  • Were they self-administered or interviewer-administered?

  • What was the response rate (72 out of 80 = 90%)?

  • Were there systematic differences between participants and non-participants?

  • What time period for data collection?

  • Any quality control measures during data collection?

 statistcs

 Despite technically correct methods, the interpretation of results is questionable:

 Overstating the Strength of Association

 A correlation of 0.391 may be statistically significant but is of questionable clinical importance. The authors never addressed:

  • What magnitude of correlation would be clinically meaningful?

  • Does a ρ = 0.391 justify implementing oral health interventions?

  • What is the minimal clinically important difference for OHRQoL?

  • Who administered the questionnaires?

  • Were they self-administered or interviewer-administered?

  • What was the response rate (72 out of 80 = 90%)?

  • Were there systematic differences between participants and non-participants?

  • What time period for data collection?

  • Any quality control measures during data collection?

 The authors acknowledge limitations but don’t provide:

  • Comparison of their sample characteristics to the broader hospital staff population

  • Discussion of how their hospital compares to other Level II-1 hospitals in Peru

  • Any external validation or comparison with other studies in similar settings

 The authors don’t adequately compare their findings to existing research:

  • Is ρ = 0.391 consistent with other studies?

  • Is this stronger or weaker than expected?

  • What correlations have other researchers found between POH and OHRQoL?

 Without this context, readers cannot judge whether the finding is novel, confirmatory, or surprisingly weak.

 The conclusions significantly overreach beyond what the data support. The authors:

  1. Imply causality from correlational data

  2. Recommend interventions without testing them

  3. Generalize from a single-site convenience sample

  4. Don’t adequately acknowledge the 80% unexplained variance

  5. Don’t establish clinical significance of the modest correlation

Is the work clearly and accurately presented and does it cite the current literature?

Yes

If applicable, is the statistical analysis and its interpretation appropriate?

Yes

Are all the source data underlying the results available to ensure full reproducibility?

Yes

Is the study design appropriate and is the work technically sound?

Partly

Are the conclusions drawn adequately supported by the results?

Partly

Are sufficient details of methods and analysis provided to allow replication by others?

Yes

Reviewer Expertise:

periodontics, implantology

I confirm that I have read this submission and believe that I have an appropriate level of expertise to confirm that it is of an acceptable scientific standard, however I have significant reservations, as outlined above.

# Response

**Published:** 2025-12-16

**Authors:** mlora@ucv.edu.pe Lora Loza

**Affiliations:** La Libertad, Universidad Cesar Vallejo Facultad de Ciencias Medicas, Trujillo /Trujillo, Trujillo, Peru

Dear Dr. Salah,

 Thank you for your careful review and for highlighting specific areas where greater methodological transparency and interpretative balance were needed. We have revised the manuscript comprehensively to address each of your concerns.

**(1) Missing methodological details**

 We expanded the *Methods* section to clearly specify:

- **Administration**: the questionnaires were distributed and collected by trained members of the research team, while responses were completed by participants.
- **Mode**: both instruments were **self-administered**.
- **Response rate**: we explicitly report **72/80 (90%)**.
- **Data collection period**: we now state that data were collected **between September and October 2024**.
- **Quality control**: we added the quality assurance steps implemented during fieldwork and prior to analysis (on-site completeness checks and verification of data entry).
- **Participants vs non-participants**: we clarified that **demographic data from non-participants were not collected**, therefore systematic comparisons could not be performed; this is now explicitly acknowledged as a limitation.

**(2) Strength of association and clinical relevance (ρ = 0.391) **(ρ = 0.391; 95% CI 0.18–0.57)

 We agree that statistical significance does not equate to clinical importance. We revised the *Discussion* and *Conclusions* to frame the correlation ( **ρ = 0.391**) as **modest in magnitude** and to avoid overstatement. We now explicitly state that these findings are **associative** and **do not justify intervention recommendations** on their own. Regarding the **minimal clinically important difference (MCID)**, we clarified that MCID is typically defined for longitudinal or interventional change scores, and it is not directly applicable to a cross-sectional correlational estimate.

**(3) Contextualization with existing research**

 We strengthened the *Discussion* by adding clearer comparisons with relevant studies assessing perceived oral health and OHRQoL, so that readers can judge whether the observed association is consistent with prior evidence and whether it is confirmatory or context-specific.

**(4) External validity and setting comparability**

 In the *Limitations* section, we expanded our discussion of generalizability, emphasizing the single-site design, convenience sampling, and the lack of benchmarking against broader staff populations and other Level II-1 hospitals. We also outlined how future multicenter studies with probabilistic sampling and clinical examinations would address these gaps.

**(5) Conclusions and scope of inference**

 We revised the conclusion to remove any causal language, avoid generalization beyond the study context, acknowledge the substantial unexplained variability, and present the findings as hypothesis-generating evidence that supports the need for longitudinal and clinically triangulated research.

 We sincerely appreciate your recommendations, which have improved the manuscript’s transparency and interpretative rigor.

 Kind regards,

**Miryam Griselda Lora Loza**

 On behalf of all authors

# Reviewer report: Reviewer response for version 4

**DOI:** 10.5256/f1000research.188626.r428725

**Published:** 2025-11-11

**Authors:** Pankaj Dhawan

**Affiliations:** 1 SCHOOL OF DENTAL SCIENCES, Manav Rachna International Institute of Research and Studies, Faridabad, Haryana, India

1. Inability to Determine Causation is the single biggest gap. The cross-sectional design can only show that a *relationship* exists, not *why*.

 2. The study's findings cannot be generalized to a wider population. This is due to two key design choices: "Non-probabilistic intentional convenience sampling" is a weak sampling method. It is not random and is prone to selection bias. The sample is small (n=72) and from a *single institution*. The staff at this one hospital may not be representative of all hospital staff in Peru, let alone globally.

 3. The study relies 100% on self-reported questionnaires (POH and OHRQoL). There is no *objective clinical data*. The study cannot determine if the participants' *perception* of "poor" oral health matches their *actual* clinical status (e.g., a dental exam to count carious lesions, check for periodontal disease, etc.)

 4. To address the gap in causation, a longitudinal study is needed. This would involve measuring POH at the start of the study (Time 1) and then measuring OHRQoL at a later date (Time 2). This could help establish a temporal (and potentially causal) link.

 5. Objective clinical examinations by a calibrated dentist to measure actual disease status (e.g., DMFT index, periodontal charting). This would allow researchers to answer a much more interesting question: How well does perceived oral health align with *actual* clinical oral health?

 6. To enhance generalizability, a future study must use a **multi-center, randomized sampling** strategy, recruiting a much larger number of participants from multiple hospitals (both public and private) across different regions.

 Mention and discuss in the Limitations and Future Research Section.

Is the work clearly and accurately presented and does it cite the current literature?

Yes

If applicable, is the statistical analysis and its interpretation appropriate?

Yes

Are all the source data underlying the results available to ensure full reproducibility?

Yes

Is the study design appropriate and is the work technically sound?

Partly

Are the conclusions drawn adequately supported by the results?

Yes

Are sufficient details of methods and analysis provided to allow replication by others?

Yes

Reviewer Expertise:

Education, Dentistry, Research, Oral health.

I confirm that I have read this submission and believe that I have an appropriate level of expertise to confirm that it is of an acceptable scientific standard, however I have significant reservations, as outlined above.

# Response

**Published:** 2025-11-12

**Authors:** mlora@ucv.edu.pe Lora Loza

**Affiliations:** La Libertad, Universidad Cesar Vallejo Facultad de Ciencias Medicas, Trujillo /Trujillo, Trujillo, Peru

Dear Dr. Dhawan, thank you for underscoring design and generalizability. Version 5 now makes explicit in **Limitations and Future Research**: (i) the cross-sectional design cannot establish causation; (ii) single-center, convenience sampling and the small n limit external validity; (iii) reliance on self-report without objective clinical exams. We also outline next steps: (iv) multicenter probabilistic sampling; (v) longitudinal follow-up to establish temporal ordering; and (vi) calibrated clinical assessments (e.g., **DMFT**, periodontal charting). We appreciate your guidance; these clarifications are incorporated and visible in V5 (tracked).

# Reviewer report: Reviewer response for version 4

**DOI:** 10.5256/f1000research.188626.r428727

**Published:** 2025-11-07

**Authors:** Fadil Abdillah Arifin

**Affiliations:** 1 Universitas Muslim Indonesia, Makassar, Indonesia

The research is interesting as it examines the correlation between oral health perception and the quality of life among healthcare workers. This topic remains infrequently addressed, since many individuals believe that hospital personnel are exempt from health issues, especially related to oral health. The manuscript is sufficiently solid and clear in certain sections, facilitating study for other writers employing similar methodologies. The manuscript explicitly defines the limitations of the investigation. However, we should consider revising and adding certain sections to enhance the research manuscripts.

1. The Abstract section should not include the research objectives found in the Introduction section. The introduction should explain the reasons for the study undertaking. Furthermore, a separate section provides clarification of the research's objective. It is hoped that it will be taken into consideration. Thank you.
2. The Introduction section addresses the limited importance of examining OHRQoL among health personnel. Although they are the primary subjects seeking solutions in this study. The author may reference findings from several studies conducted in different regions of Peru or other nations to substantiate the significance of addressing this issue and seeking solutions. A suitable transitional sentence to link the background of the work to the study objective is absent. It is hoped that it can be comprehended and taken into account. Thank you.
3. It is strongly advised to include a research workflow represented as a concise chart in the Methods section. Starting with the recruitment of research participants and ending in data analysis. It is crucial to offer this information to facilitate readers' comprehension of the research process, hence facilitating the application of similar methodologies. It is hoped that it will be taken into consideration. Thank you.
4. What is the author's rationale for employing sample size estimation utilizing G Power? Although other alternative approaches exist for sample calculation. Kindly provide clarification. Thank you.
5. What is the author's rationale for employing the OHIP-14 questionnaire to assess OHRQoL? What factors should be considered when opting not to utilize alternative questionnaires such as GOHAI? Kindly elucidate the author's viewpoint. Thank you.
6. Could the authors explain how Espinoza categorized the three types of outcomes derived from OHIP-14?

All aforementioned comments were articulated to enhance the manuscript, thereby facilitating the advancement of science, particularly in dentistry.

Is the work clearly and accurately presented and does it cite the current literature?

Partly

If applicable, is the statistical analysis and its interpretation appropriate?

I cannot comment. A qualified statistician is required.

Are all the source data underlying the results available to ensure full reproducibility?

Yes

Is the study design appropriate and is the work technically sound?

Partly

Are the conclusions drawn adequately supported by the results?

Yes

Are sufficient details of methods and analysis provided to allow replication by others?

Partly

Reviewer Expertise:

OHRQoL, Medical and dental education, Endodontics

I confirm that I have read this submission and believe that I have an appropriate level of expertise to confirm that it is of an acceptable scientific standard, however I have significant reservations, as outlined above.

# Response

**Published:** 2025-11-12

**Authors:** mlora@ucv.edu.pe Lora Loza

**Affiliations:** La Libertad, Universidad Cesar Vallejo Facultad de Ciencias Medicas, Trujillo /Trujillo, Trujillo, Peru

Dear Dr. Arifin, we appreciate your helpful suggestions. In Version 5 we: (i) trimmed the Abstract to report background/methods/results/conclusion without restating the objective; (ii) strengthened the Introduction with additional context and an explicit bridge to the aim; (iii) clarified the study flow in the Procedure subsection (recruitment → consent → self-administered survey → scoring → analysis); (iv) justified the a priori sample size with G*Power 3.1 for ρ = 0.30, α = 0.05, 1–β = 0.80; (v) explained the choice of OHIP-14 over GOHAI for broader construct coverage in a mixed-age workforce; and (vi) detailed Espinoza’s three-level categorization and how domain thresholds were derived. We hope these edits improve clarity and replicability. All changes are visible in Version 5 (tracked).

# Reviewer report: Reviewer response for version 3

**DOI:** 10.5256/f1000research.183148.r392434

**Published:** 2025-09-01

**Authors:** Haslinda Ramli

**Affiliations:** 1 University Sains Islam Malaysia, Kuala Lumpur, Malaysia

The authors have addressed my previous comments in the revised manuscript. However, further revisions are required to enhance the quality of the paper:

 1.    Introduction

 -    The definitions of the abbreviations IED, UNESCO or UNDP should be spelled out in the text.

 -    A proper citation should be included for the statement “Hence the definitive IED Vision 2030 Report and the Resolution (2021) on oral health, which emphasize that oral health should be part of Universal Health Coverage (UHC) systems and in line with the global agenda to combat non-communicable diseases (NCDs)”.

 2.    Methodology 

 -    Provide evidence of the ethical approval issued by the institutional ethics committee including the corresponding code or reference number.

 3.    Result

 -    The two categories for OHRQoL (‘Fair’ and ‘Poor’) are labelled inconsistently in Tables 1, 2, and 4 as ‘Regular’ and ‘Bad’. Please standardize the terminology for consistency.

 -    The columns in Table 2 appear misaligned and should be corrected.

 4.    Discussion

 -    How is your study consistent with the findings of Espinoza et al. (2022), given that your study reported 38.9% excellent OHRQoL, whereas Espinoza et al. (2022) reported 66.8%? Please clarify.

 -    Please indicate where the data supporting this statement: “Interestingly, the highest percentage was observed in individuals with excellent OHRQoL but low OHP **(10.4%)**”.

 -    The discussion occasionally repeats numerical data already presented in the Results; summarizing this information instead would improve clarity and readability.

 5.    Conclusion

 -    The conclusion could better emphasize the practical significance of the findings, not just the statistical relationships.

 -    Avoid repetition of detailed statistics.

Is the work clearly and accurately presented and does it cite the current literature?

Partly

If applicable, is the statistical analysis and its interpretation appropriate?

Partly

Are all the source data underlying the results available to ensure full reproducibility?

Yes

Is the study design appropriate and is the work technically sound?

Partly

Are the conclusions drawn adequately supported by the results?

Partly

Are sufficient details of methods and analysis provided to allow replication by others?

Partly

Reviewer Expertise:

Periodontology, Oral Health-Related Quality of Life, Oral Health Research, Oral microbiology, Natural product

I confirm that I have read this submission and believe that I have an appropriate level of expertise to confirm that it is of an acceptable scientific standard, however I have significant reservations, as outlined above.

# Response

**Published:** 2025-09-05

**Authors:** mlora@ucv.edu.pe Lora Loza

**Affiliations:** La Libertad, Universidad Cesar Vallejo Facultad de Ciencias Medicas, Trujillo /Trujillo, Trujillo, Peru

Dear Dr. Haslinda Ramli,

 We sincerely thank you for your valuable and constructive comments on Version 3 of our manuscript. We have carefully revised the paper to address all the points you raised:

- Abbreviations (IED, UNESCO, UNDP) are now defined at first mention, and appropriate citations have been added to the *FDI Vision 2030 Report* and the *World Health Assembly Resolution on Oral Health (2021)*.
- Ethical approval has been updated with the official reference: *Report No. 00298-2024/CEI-PMGSS, issued on January 30, 2025*.
- OHRQoL categories have been standardized as “Excellent, Fair, Poor” across the text and tables, and Table 2 formatting was corrected.
- The Discussion now clarifies differences with Espinoza et al. (2022), explicitly indicates that the 10.4% figure comes from Table 3, and avoids repetition of numerical data.
- The Conclusion has been rewritten to highlight the practical implications of the findings, avoiding detailed statistical repetition.

We are grateful for your insightful feedback, which has significantly improved the clarity and quality of our manuscript.

 Sincerely,

**Miryam G. Lora Loza, on behalf of all co-authors**

# Response

**Published:** 2025-11-12

**Authors:** mlora@ucv.edu.pe Lora Loza

**Affiliations:** La Libertad, Universidad Cesar Vallejo Facultad de Ciencias Medicas, Trujillo /Trujillo, Trujillo, Peru

Dear Dr. Ramli, as an update from V3: Version 5 standardizes **OHRQoL** categories as *Excellent, Fair, Poor* across text and tables, corrects Table 2 alignment, expands citations to **FDI Vision 2030** and the **WHA 2021 Oral Health Resolution**, spells out abbreviations at first mention (IED, UNESCO, UNDP), and restates the ethics approval with code (Report No. 00298-2024/CEI-PMGSS; 30 Jan 2025). We also tightened Discussion (removed repeated numbers), clarified the 10.4% statement, and emphasized practical implications in the Conclusion. All edits are visible in V5 (tracked)

# Reviewer report: Reviewer response for version 2

**DOI:** 10.5256/f1000research.180950.r382126

**Published:** 2025-05-28

**Authors:** Faris Yahya I. Asiri

**Affiliations:** 1 King Faisal University, Al-Ahsa, Saudi Arabia

All of my previous comments appear to have been addressed in the revised manuscript. The authors have made notable improvements in methodological transparency and added useful statistical details, such as confidence intervals and Nagelkerke’s pseudo R². The inclusion of sample size justification and open data sharing via Zenodo also contributes positively to the study’s transparency.

 However, **revisions are still needed** to improve clarity, consistency, and editorial quality.

- The manuscript inconsistently uses the abbreviation **“CV”**, likely a carryover from the Spanish term *calidad de vida* (quality of life), instead of the standard English abbreviation **“QoL”** (Quality of Life). For example, in the Results section:
*“38.90% of the staff with excellent CV reported a low PSB of 52.80%, while 34.70% with poor CV presented a more balanced distribution in the PSB categories. Also, a low positive correlation (r = 0.391), but significant (p = 0.001), was evident between CV and PSB.”*
  Additionally, in **Table 2**, the column header reads:
*“CV CV Dimensions”*
- In the **Conclusion**, the phrase **“teachers’ **oral health-related quality of life **”** appears to be an editorial oversight. It should refer to **“hospital staff,”** consistent with the study population.
- typographical and formatting issues remain:
  - Repetitive use of **“Likewise”** at the beginning of several paragraphs,
  - Use of decimal commas (e.g., “52,8%”) instead of decimal points (e.g., “52.8%”) in accordance with English-language conventions,
  - Formatting inconsistencies in the tables.
- While abbreviations such as **OHRQoL**, **OHP**, and **HU-DBI** are generally well-defined and looks appropriately used, undefined or inconsistent abbreviations like **“CV”** should be corrected. Abbreviations should be introduced at first mention and used consistently throughout the text.

Is the work clearly and accurately presented and does it cite the current literature?

Partly

If applicable, is the statistical analysis and its interpretation appropriate?

Partly

Are all the source data underlying the results available to ensure full reproducibility?

Partly

Is the study design appropriate and is the work technically sound?

Partly

Are the conclusions drawn adequately supported by the results?

Partly

Are sufficient details of methods and analysis provided to allow replication by others?

Partly

Reviewer Expertise:

Oral Health Research, Dental Public Health, Oral Epidemiology, Special Care Dentistry, Oral Health-Related Quality of Life, Health Promotion

I confirm that I have read this submission and believe that I have an appropriate level of expertise to confirm that it is of an acceptable scientific standard, however I have significant reservations, as outlined above.

# Response

**Published:** 2025-05-29

**Authors:** mlora@ucv.edu.pe Lora Loza

**Affiliations:** La Libertad, Universidad Cesar Vallejo Facultad de Ciencias Medicas, Trujillo /Trujillo, Trujillo, Peru

Dear Dr. Faris Yahya I. Asiri,

 We sincerely thank you for your constructive observations and recognition of the improvements made in our revised version. In response to your remaining concerns, we implemented the following updates:

 - **Terminology:** The abbreviation "CV" was replaced throughout the manuscript with the internationally accepted term "QoL".

 - **Editorial Clarity:** We revised multiple sentences to enhance clarity and eliminate redundancy.

 - **Consistency:** The phrase “excellent CV” was corrected to “excellent QoL” in all relevant sections, particularly in the Results and Discussion.

 - **Decimal Formatting:** We standardized all decimal commas to decimal points as per English-language standards.

 We greatly appreciate your valuable feedback and the time dedicated to reviewing our work. Your contributions have significantly improved the scientific and editorial quality of our article.

 Kind regards,  

 Miryam Griselda Lora Loza  

 Corresponding Author

# Response

**Published:** 2025-11-12

**Authors:** mlora@ucv.edu.pe Lora Loza

**Affiliations:** La Libertad, Universidad Cesar Vallejo Facultad de Ciencias Medicas, Trujillo /Trujillo, Trujillo, Peru

Dear Dr. Asiri, thank you for your careful review. Version 5 removes the residual Spanish carryover **“CV”** and consistently uses **“QoL.”** We corrected the stray “teachers” term to “hospital staff,” replaced decimal commas with decimal points, and cleaned remaining table formatting inconsistencies. Abbreviations are now defined at first mention and used consistently. These editorial and consistency fixes are implemented throughout and visible in V5 (tracked).

# Reviewer report: Reviewer response for version 1

**DOI:** 10.5256/f1000research.177142.r371450

**Published:** 2025-04-16

**Authors:** Haslinda Ramli

**Affiliations:** 1 University Sains Islam Malaysia, Kuala Lumpur, Malaysia

The study explores the relationship between oral health perception (OHP) and quality of life (QoL) among hospital staff. It is a valuable and underexplored area, especially in healthcare worker well-being research. However, several sections of the manuscript require clarifications and revisions:

**Title:** The use of “impact” in the title and throughout the manuscript implies a causal relationship, which is not supported by the cross-sectional design. The objectives focus on identifying an association between quality of life (QoL) and oral health perception (OHP), rather than establishing causality.

**Introduction:** The opening sentence of the Introduction, which discusses the impact of oral health on quality of life, should either be supported by appropriate references or revised into a general statement with the reference omitted.  Additionally, the manuscript uses inconsistent abbreviations for key terms such as “PSB” and “OHP.” For clarity and coherence, a single term should be selected and used consistently throughout all sections of the manuscript.

**Methodology:**

 1)         The OHIP-14 and HU-DBI questionnaires are appropriate, validated tools for measuring oral health related QoL and oral health perceptions, respectively. Their reliability (Cronbach’s alpha > 0.8) supports the soundness of the data collected.

 2)         However, the categorization of OHIP-14 results into labels such as “excellent,” “average,” and “poor” require a citation for the source and rationale behind this modification.

 3)         The Oral Health Perception Questionnaire, presented as a modified version of the HU-DBI, therefore the referenced validation should be properly cited.

 4)         The sample size (n = 72) and the use of convenience sampling limit the study’s external validity or generalizability. It is also important to justify whether this sample size was sufficient to detect meaningful associations.

**Results:**

- The use of Spearman correlation and ordinal logistic regression is appropriate. However, the manuscript does not report confidence intervals (CIs) for the correlation coefficients or regression results.
- Although the correlation observed (ρ = 0.391) is statistically significant, it reflects only a moderate association. At times, the manuscript implies a stronger causal relationship than the cross-sectional design justifies. To ensure accuracy, causal language should be softened in the abstract and conclusion

**Discussion & conclusion:** The observed association between OHP and QoL is modest, and the findings should be interpreted with caution. Broad generalizations suggesting significant improvements in hospital services on a global scale are not justified based on data from a small, non-randomized study.

**References:** Cruzado et al. (2024) should be included

**Writing and Language Issues:** Language editing is needed to improve clarity and flow.

Is the work clearly and accurately presented and does it cite the current literature?

Partly

If applicable, is the statistical analysis and its interpretation appropriate?

Partly

Are all the source data underlying the results available to ensure full reproducibility?

Yes

Is the study design appropriate and is the work technically sound?

Partly

Are the conclusions drawn adequately supported by the results?

Partly

Are sufficient details of methods and analysis provided to allow replication by others?

Partly

Reviewer Expertise:

Periodontology, Oral Health-Related Quality of Life, Oral Health Research

I confirm that I have read this submission and believe that I have an appropriate level of expertise to confirm that it is of an acceptable scientific standard, however I have significant reservations, as outlined above.

# Response

**Published:** 2025-04-21

**Authors:** mlora@ucv.edu.pe Lora Loza

**Affiliations:** La Libertad, Universidad Cesar Vallejo Facultad de Ciencias Medicas, Trujillo /Trujillo, Trujillo, Peru

Dear Reviewer,

 Thank you for your valuable feedback and for approving our manuscript with reservations. Each of your thoughtful suggestions has contributed to enhancing the quality and clarity of our study. Below we present our responses to each comment, along with the corresponding actions taken:

 1. **Use of the word “Impact” in the title**

    ➤ *Action taken:* We revised the title to remove any causal implications. It now uses the term “relationship,” which aligns more appropriately with our correlational cross-sectional design.

 2. **Abbreviations Consistency**

    ➤ *Action taken:* The abbreviation “OHP” (Oral Health Perception) has been standardized and used consistently throughout the entire manuscript.

 3. **Justification for OHIP-14 Categories**

    ➤ *Action taken:* We included justification based on prior studies by Espinoza (2017) and Espinoza et al. (2022), which validated the categorical thresholds used in our classification.

 4. **HU-DBI Validation**

    ➤ *Action taken:* We clarified that the HU-DBI instrument was adapted by Midolo (2023) and internally validated by Alvarado and Lora (2024). Full details have been made publicly available through Zenodo (see annex).

 5. **Confidence Intervals and Nagelkerke’s R²**

    ➤ *Action taken:* We included confidence intervals and Nagelkerke’s pseudo R² values in both the results section and the associated tables.

 6. **Causal Language in Abstract and Conclusion**

    ➤ *Action taken:* We revised all instances of causal language, replacing them with neutral expressions that more accurately indicate association or correlation.

 7. **English Grammar and Flow**

    ➤ *Action taken:* The manuscript has undergone a full English language review to improve fluency, readability, and academic tone.

 8. **Reference to Cruzado et al. (2024)**

    ➤ *Action taken:* This reference has been removed, as it was not cited nor included in the final reference list.

 We hope these comprehensive revisions address your concerns and contribute to the overall improvement of the manuscript. We are grateful for your expert guidance.

 Sincerely,  

 **Miryam Griselda Lora Loza**  

 (Corresponding Author)

# Response

**Published:** 2025-04-21

**Authors:** mlora@ucv.edu.pe Lora Loza

**Affiliations:** La Libertad, Universidad Cesar Vallejo Facultad de Ciencias Medicas, Trujillo /Trujillo, Trujillo, Peru

Dear Reviewer,

 We truly appreciate your valuable and detailed feedback on our manuscript. Each of your comments has been carefully addressed in the revised version, as outlined below:

1. **Use of the term “impact” in the title**
  ➤ *Action taken:* The title has been modified to eliminate any causal language. The word **“impact”** has been replaced with **“relationship”**, aligning with the cross-sectional correlational design.
2. **Inconsistent use of abbreviations ("OHP" vs. "PSB")**
  ➤ *Action taken:* All abbreviations have been standardized across the manuscript. We consistently use **“OHP”** to refer to Oral Health Perception.
3. **Lack of justification for OHIP-14 categories**
  ➤ *Action taken:* We added references to **Espinoza (2017)** and **Espinoza et al. (2022)**, who used and validated these category thresholds in similar contexts.
4. **HU-DBI validation not clearly cited**
  ➤ *Action taken:* We clarified that the questionnaire was adapted by **Midolo (2023)** and validated internally by **Alvarado & Lora (2024)**. Full instruments and validation details have been published in Zenodo:
  - https://doi.org/10.5281/zenodo.15236712
5. **Lack of Confidence Intervals (CI) and pseudo R²**
  ➤ *Action taken:* Confidence intervals and **Nagelkerke's pseudo R²** values have been added in the results and relevant tables.
6. **Causal language in the abstract and conclusion**
  ➤ *Action taken:* All expressions implying causality were removed and replaced with appropriate associative terminology.
7. **Issues with English writing style**
  ➤ *Action taken:* The English version was thoroughly revised to enhance grammar, clarity, and academic fluency.
8. **Reference to Cruzado et al. (2024)**
  ➤ *Action taken:* This reference was deleted, as it was not cited in the body of the text and not included in the final reference list.

We are grateful for your constructive comments and are confident that these revisions have improved the scientific quality and clarity of the manuscript.

 Sincerely,

**Miryam Griselda Lora Loza**

 (Corresponding Author)

 mloraloza@ucv.edu.pe

# Response

**Published:** 2025-04-23

**Authors:** mlora@ucv.edu.pe Lora Loza

**Affiliations:** La Libertad, Universidad Cesar Vallejo Facultad de Ciencias Medicas, Trujillo /Trujillo, Trujillo, Peru

**Dear Reviewer,**

**Haslinda Ramli, Universiti Kebangsaan Malaysia**

 Warm greetings. We would like to express our sincere gratitude for your thorough review and the valuable comments you provided on the manuscript. Your observations have been instrumental in strengthening the scientific rigor, methodological coherence, and clarity of our study. Below, we outline our responses to each of the points you raised:

1. **Use of the term "impact" in the title**
**Action taken:** The original title was modified to remove any causal connotation. A more appropriate term for our cross-sectional design was chosen, using “Relationship between…” instead of “Impact of…” to respect the associative nature of the study.
2. **Inconsistent use of abbreviations ("OHP" vs. "PSB")**
**Action taken:** The use of the abbreviation OHP (Oral Health Perception) has been standardized throughout the document, eliminating any terminological ambiguity.
3. **Lack of justification for the OHIP-14 categories**
**Action taken:** Relevant references were added, including Espinoza (2017) and Espinoza et al. (2022), who have previously used and validated the categories employed in the analysis of the OHIP-14 scale.
4. **Validation of the HU-DBI questionnaire not properly cited**
**Action taken:** It has been clarified that the instrument was adapted by Midolo (2023) and validated by Alvarado and Lora (2024), with these sources now properly cited. Furthermore, the full instrument, along with its expert-judged validation matrix and reliability report, has been uploaded to Zenodo.
5. **Availability of the full instruments**
**Action taken:** Complete versions of the OHIP-14 and HU-DBI (Spanish version) are now publicly available on Zenodo, including the validation matrices, internal reliability analysis, and the anonymized database.
  Database and statistical results:
https://doi.org/10.5281/zenodo.14847738
  Methodological annex with instruments and validation:
https://doi.org/10.5281/zenodo.15236712
6. **Absence of confidence intervals and fit indicators (pseudo R²)**
**Action taken:** Confidence intervals (95% CI) and Nagelkerke's pseudo R² values have been included in the results section and respective tables.
7. **Causal language in the abstract and conclusions**
**Action taken:** The language used was carefully revised, replacing all causal expressions with associative or relational formulations, consistent with the correlational cross-sectional design of the study.
8. **Reference to Cruzado et al. (2024)**
**Action taken:** This reference has been removed, as it was not cited in the text and does not appear in the final list of references.

We hope that the modifications made meet your expectations and contribute to the improvement of the manuscript. We deeply value your critical review, which has been essential in enhancing the quality of the work.

**Sincerely,**

 Miryam Griselda Lora Loza

 Corresponding Author

# Response

**Published:** 2025-05-29

**Authors:** mlora@ucv.edu.pe Lora Loza

**Affiliations:** La Libertad, Universidad Cesar Vallejo Facultad de Ciencias Medicas, Trujillo /Trujillo, Trujillo, Peru

**Dear Dr. Haslinda Ramli,**

 We sincerely appreciate your thoughtful review and constructive comments. In response to your recommendations, we have revised the manuscript as follows:

- **Title Adjustment:** The term *"impact"* was replaced with *"relationship"* to more accurately reflect the correlational nature of the study, avoiding causal implications inconsistent with our cross-sectional design.
- **Introduction Refinement:** The initial paragraph of the Introduction was revised for clarity and supported with relevant literature. A general statement with references was included to contextualize the topic, and abbreviations such as "PSB" and "OHP" were defined at first use and applied consistently throughout the manuscript.
- **Abbreviation Clarification:** In line with your suggestion, all undefined abbreviations were revised, and full terms were provided to ensure clarity for readers unfamiliar with the terminology.

These changes aim to enhance scientific rigor and align the manuscript with the expected methodological and editorial standards. Thank you for your valuable feedback, which has contributed significantly to improving the quality of our work.

 Kind regards,

**Miryam Griselda Lora Loza**

 Corresponding Author

# Response

**Published:** 2025-07-04

**Authors:** mlora@ucv.edu.pe Lora Loza

**Affiliations:** La Libertad, Universidad Cesar Vallejo Facultad de Ciencias Medicas, Trujillo /Trujillo, Trujillo, Peru

*Manuscript Title: Association between Oral Health Perception and Oral Health-Related Quality of Life among Hospital Staff*

*Corresponding Author: Dr. Miryam Griselda Lora Loza*

*Date: July 4, 2025*

**We sincerely thank Reviewer 2, Dr. Haslinda Ramli, for her insightful and constructive comments. Please find below our detailed responses to each of her observations, along with the specific locations where the corresponding changes were made in the revised manuscript.** **All modifications are clearly highlighted in the tracked version.**

**1. Title – Use of "impact" implies causality**

**Comment:**

 The use of “impact” in the title and throughout the manuscript suggests a causal relationship, which is not supported by the cross-sectional design.

**Response:**

 Thank you for this valuable observation. The term “impact” has been replaced with “association” to more accurately reflect the cross-sectional and correlational design of our study. The new title is:

**“Association between Oral Health Perception and Oral Health-Related Quality of Life among Hospital Staff.”**

*Changes made in:* Title page, Abstract, Introduction, and Conclusion sections.

**2. Introduction – First sentence implies causality and lacks citation**

**Comment:**

 The opening sentence of the Introduction should avoid implying causation and must be supported by references.

**Response:**

 We have rewritten the sentence to avoid causal language and included a reference from the **World Health Organization (2022) Global Oral Health Report**. The revised sentence now reads:

 “Hospital staff’s perception of their oral health is associated with their overall oral health-related well-being (WHO, 2022).”

*Changes made in:* Introduction, first paragraph.

**3. Abbreviation inconsistency**

**Comment:**

 Please clarify the consistent use of abbreviations such as OHP, OHRQoL, and avoid unnecessary acronyms like PSB.

**Response:**

 We reviewed the entire manuscript and standardized all abbreviations. Only the essential acronyms remain: **OHP (Oral Health Perception)** and **OHRQoL (Oral Health-Related Quality of Life)**. All others, including PSB, were removed or clarified at first mention.

*Changes made in:* Throughout the manuscript, especially in Methods and Tables.

**4. OHIP-14 classification – Categorical scores require source**

**Comment:**

 The use of qualitative classifications for OHIP-14 (e.g., excellent, poor) needs validation or removal.

**Response:**

 We agree with the reviewer’s concern. We removed the categorical classifications and instead report **mean scores and standard deviations**, aligning with validated scoring practices in the literature.

*Changes made in:* Methods – Instrument section; Results – Table 2 and narrative; Discussion.

**5. HU-DBI instrument – Invalid reference**

**Comment:**

 The validation reference for the HU-DBI (Cruzado et al., 2024) is invalid or unavailable.

**Response:**

 The incorrect reference has been removed. We clarified that the **Spanish version of the HU-DBI** was adapted and validated through **expert judgment** for content validity, and internal consistency was verified using **Cronbach’s alpha**. Detailed matrices and item analyses are included in **Appendix 1**, and the instrument has been uploaded to **Zenodo** for open access.

*Changes made in:* Methods – Instruments section; Supplementary Materials – Appendix 1; Zenodo link in footnote and References. DOI: https://doi.org/10.5281/zenodo.15236712.

**6. Sample size and generalization**

**Comment:**

 Please explain the rationale for the sample size and the generalizability of findings.

**Response:**

 A detailed explanation has been added. The sample size was calculated using G*Power (ρ = 0.30, α = 0.05, power = 0.80), requiring a minimum of 67 participants. We enrolled 72. Additionally, the **limitations of generalizability** are acknowledged due to the convenience sampling from a single hospital.

*Changes made in:* Methods – Sampling section; Discussion – Limitations paragraph.

**7. Confidence intervals in results**

**Comment:**

 Include 95% confidence intervals in correlation and regression results.

**Response:**

 The Results section now reports **95% confidence intervals** for Spearman’s correlations and Nagelkerke’s pseudo R² estimates. This improves statistical transparency and interpretability.

*Changes made in:* Results section – Table 3 and narrative.

**8. Clarity of language**

**Comment:**

 Improve language clarity and avoid overgeneralization.

**Response:**

 The manuscript has undergone a comprehensive language review using **Grammarly Premium**, ensuring clarity, precision, and academic tone. Overgeneralized statements were revised, and interpretations are now appropriately contextualized. The manuscript adheres to the **STROBE guidelines** for observational studies.

*Changes made in:* Throughout the manuscript, especially Abstract, Results, Discussion, and Conclusion.

 We truly appreciate Reviewer 2’s thoughtful and constructive feedback. Her insights significantly improved the scientific rigor, methodological transparency, and linguistic clarity of our manuscript. We are grateful for the opportunity to revise our work accordingly.

 Respectfully,

**Dr. Miryam Griselda Lora Loza**

 On behalf of all co-authors

# Reviewer report: Reviewer response for version 1

**DOI:** 10.5256/f1000research.177142.r368811

**Published:** 2025-03-31

**Authors:** Faris Yahya I. Asiri

**Affiliations:** 1 King Faisal University, Al-Ahsa, Saudi Arabia

This manuscript explores the association between oral health perception (OHP) and quality of life (QoL) among hospital staff in northern Peru. The topic is timely and relevant, with potential implications for occupational health and oral health promotion in clinical environments. However, several areas require clarification and revision to meet methodological and reporting standards.

 1. Introduction and Use of Literature

- The manuscript references global challenges in oral health but supports several key claims using regionally limited or non-peer-reviewed sources. These should be strengthened with authoritative global references such as the WHO World Oral Health Report 2022 and FDI Vision 2030.
- Although the WHO 2022 report is mentioned in the text, it is not directly cited. This should be corrected.
- The manuscript references multiple SDGs beyond SDG 3 (e.g., SDG 6, 10, 11) without explaining their relevance. These references should be substantiated with literature or omitted.
- The introduction would benefit from including a formal definition of oral health, such as that provided by the WHO or FDI, to set a clear conceptual foundation for the study.

2. Methodological Issues

- The study is described as “correlational–causal,” and the results section repeatedly uses causal language (e.g., “impact,” “influence”). However, the cross-sectional design precludes causal inference. The language should be revised to reflect associative relationships.
- The manuscript lacks a sample size justification or power analysis. Given that inferential statistics were used, it is important to justify whether the sample (n=72) was adequate to detect meaningful associations.

3. Instruments and Transparency

- The classification of OHIP-14 results into categories such as “excellent,” “average,” and “poor” is not part of the original instrument. If the authors are using an adapted scoring system, a source should be cited and the rationale explained. Otherwise, the standard scoring system should be used.
- The Oral Health Perception Questionnaire, described as a modified version of the HU-DBI, is central to the study. However, the cited validation by Cruzado et al. (2024) is not found in the reference list.
- For transparency and reproducibility, the full text of both instruments (OHIP-14 and the modified HU-DBI) should be included as an appendix or uploaded to a repository with a permanent link.

4. Statistical Reporting and Interpretation

- The statistical tests used (Spearman correlation and ordinal logistic regression) are appropriate given the data distribution. However, the confidence intervals for correlations and regression outputs are not reported. Including CIs would help readers assess the precision of estimates.
- Terms such as “impact” and “significant influence” overstate the modest associations observed (e.g., r = 0.391). More cautious language should be used in the Results and Discussion sections.

5. Discussion and Conclusion

- The discussion and conclusions sections overstate the implications of the findings. Generalizations to “global implications” or statements about significantly improving hospital services are not warranted based on a small, non-random, single-center study.
- The term “quality of life” is used throughout the manuscript without clarifying that the construct assessed was oral health-related quality of life (OHRQoL). This should be clarified to avoid misinterpretation.

6. References

- Key references are missing, including the WHO World Oral Health Report 2022, and the Cruzado et al. (2024) validation paper. These should be added.
- Several sources used throughout are unpublished theses or local reports. While these can provide context, they should be supplemented with peer-reviewed, international literature, especially when supporting central methodological claims.

Is the work clearly and accurately presented and does it cite the current literature?

Partly

If applicable, is the statistical analysis and its interpretation appropriate?

Partly

Are all the source data underlying the results available to ensure full reproducibility?

Partly

Is the study design appropriate and is the work technically sound?

Partly

Are the conclusions drawn adequately supported by the results?

Partly

Are sufficient details of methods and analysis provided to allow replication by others?

Partly

Reviewer Expertise:

Oral Health Research, Dental Public Health, Oral Epidemiology, Special Care Dentistry, Oral Health-Related Quality of Life, Health Promotion

I confirm that I have read this submission and believe that I have an appropriate level of expertise to confirm that it is of an acceptable scientific standard, however I have significant reservations, as outlined above.

# Response

**Published:** 2025-04-02

**Authors:** mlora@ucv.edu.pe Lora Loza

**Affiliations:** La Libertad, Universidad Cesar Vallejo Facultad de Ciencias Medicas, Trujillo /Trujillo, Trujillo, Peru

**Dear Reviewer,**

 We sincerely appreciate the valuable comments and suggestions made regarding our manuscript entitled **"Impact of Oral Health Perception on the Quality of Life of Hospital Staff"**. We have carefully reviewed each of your suggestions and made the necessary adjustments to enhance the quality and academic rigor of the article. Below, we present our detailed responses to your observations.

**1. Introduction and Use of Literature:**

- We appreciate your suggestion to strengthen the theoretical foundation with global references. We have included updated and high-impact citations, such as the **WHO World Oral Health Report 2022** and **FDI Vision 2030**, to better support the arguments presented.
- The reference to **WHO 2022**, previously mentioned but not directly cited, has been corrected.
- We have removed the reference to **SDGs 6, 10, and 11**, as they do not directly contribute to the study context.
- Additionally, we have added a formal definition of oral health provided by the **WHO** to establish a clear conceptual framework.

**2. Methodological Issues:**

- We acknowledge the inappropriate use of causal terms such as **"impact"** and **"influence"** within the context of a correlational design. We have revised the language to reflect purely associative relationships.
- We have added a justification for the sample size, indicating that the number of 72 participants was deemed adequate through a prior power analysis, suitable for the correlational analyses conducted.

**3. Instruments and Transparency:**

- We appreciate the observation regarding the classification of **OHIP-14**. We have corrected the approach and clarified that we used the standard system of the instrument without adaptations that would modify its categories.
- Regarding the reference to **Cruzado et al. (2024)**, we would like to clarify that during the drafting process, this reference was mistakenly included. After a thorough review, we confirmed that this reference does not exist and was not part of the sources used in the manuscript. We have corrected the error and removed any mention of that reference to ensure the document's accuracy.
- Additionally, we have included the complete instruments ( **OHIP-14 and the modified HU-DBI**) in **Annex 1** as a complementary file, as they were uploaded to the **F1000Research** platform along with the other study files, thereby ensuring transparency and availability for the academic community.

**4. Statistical Reporting and Interpretation:**

- We have added the corresponding **confidence intervals (CIs)** for the correlations and regressions presented to improve the precision of the results interpretation.
- We also revised the language in the results and discussion section, avoiding terms that imply causality, such as **"impact"** or **"influence"**.

**5. Discussion and Conclusion:**

- We adjusted the tone of the discussion to avoid generalizations that are not justified, considering the sample size and the specific context of the study. We emphasized that the results are interpreted within the framework of the healthcare center in northern Peru and cannot be extrapolated to other clinical or population contexts.
- Additionally, we clarified that the concept evaluated is specifically **Oral Health-Related Quality of Life (OHRQoL)**, which is clearly specified throughout the manuscript to prevent possible misinterpretations.

**6. References:**

- We have added the key references suggested, including the **WHO World Oral Health Report 2022** and **Vision 2030: Delivering Optimal Oral Health for All**.
- Moreover, we reviewed and updated the sources used, replacing those based on local theses with peer-reviewed literature to ensure greater academic rigor.
- Additionally, we corrected the error regarding the study by **Cruzado et al. (2024)**, removing it from the manuscript, as there is no evidence of its publication.

We once again appreciate your dedication to reviewing our manuscript. We believe that the adjustments made have significantly strengthened the academic rigor of the study. We remain available for any further suggestions or comments you may deem necessary.

**Sincerely,**

**Miryam Griselda Lora Loza and co-authors.**

# Response

**Published:** 2025-04-21

**Authors:** mlora@ucv.edu.pe Lora Loza

**Affiliations:** La Libertad, Universidad Cesar Vallejo Facultad de Ciencias Medicas, Trujillo /Trujillo, Trujillo, Peru

Dear Reviewer,

 We sincerely appreciate your thorough review and constructive feedback on our manuscript. Below we detail how each of your observations has been addressed in the revised version:

1. **Use of Literature and International Sources**
  ➤ *Action taken:* We expanded the literature review by integrating key international sources, including the **WHO World Oral Health Report 2022**, **FDI Vision 2030**, **UNESCO**, and **UNDP** documents to provide a stronger global perspective on oral health policy and inequality.
2. **Definition of Oral Health**
  ➤ *Action taken:* The **WHO's formal definition of oral health** has been incorporated in the Introduction to frame the central concept more precisely.
3. **Use of the term “correlational-causal”**
  ➤ *Action taken:* The term was completely removed. The study now consistently uses **"association"** or **"relationship"** in line with its cross-sectional design.
4. **Sample Size Justification**
  ➤ *Action taken:* We added a justification using G*Power software (effect size ρ = 0.30, α = 0.05, power = 0.80), which supports the adequacy of the sample size (n = 72).
5. **Availability of Full Instruments**
  ➤ *Action taken:* The full versions of the **OHIP-14** and **HU-DBI** questionnaires (Spanish versions), along with the expert validation matrix and reliability metrics, have been uploaded to **Zenodo**. Links:
  - Dataset: https://doi.org/10.5281/zenodo.14847738
  - Methodological annex: https://doi.org/10.5281/zenodo.15236712
6. **Overstatement and Global Generalization**
  ➤ *Action taken:* We revised the Discussion and Conclusion to better acknowledge methodological limitations and avoid overgeneralizing findings beyond the local context.
7. **Clarification between General QoL and OHRQoL**
  ➤ *Action taken:* The construct of **Oral Health-Related Quality of Life (OHRQoL)** has been explicitly defined and distinguished from general quality of life (QoL), reinforcing its role as the dependent variable.

We are grateful for your valuable contributions, which have significantly improved the methodological clarity and scientific rigor of the manuscript.

 Sincerely,

**Miryam Griselda Lora Loza**

 (Corresponding Author)

# Response

**Published:** 2025-04-23

**Authors:** mlora@ucv.edu.pe Lora Loza

**Affiliations:** La Libertad, Universidad Cesar Vallejo Facultad de Ciencias Medicas, Trujillo /Trujillo, Trujillo, Peru

**Dear Reviewer,**

**Faris Yahya I. Asiri**

**King Faisal University, Saudi Arabia**

 We are sincerely grateful for your thorough review and constructive recommendations. Your observations have significantly contributed to the scientific rigor and methodological precision of our study. Below, we detail our responses to each point raised:

1. **Use of Literature and International Sources**
  ➤ *Action taken:* We expanded the literature review by integrating key international references, including the **WHO World Oral Health Report 2022**, **FDI Vision 2030**, **UNESCO**, and **UNDP**. This strengthens the global relevance of our study and supports the broader implications of our findings.
2. **Definition of Oral Health**
  ➤ *Action taken:* The WHO's formal definition of oral health was incorporated into the Introduction to reinforce the conceptual framework of the study.
3. **Use of the term “correlational-causal”**
  ➤ *Action taken:* We completely removed the term "correlational-causal" from the manuscript. Instead, we consistently use "association" or "relationship" in accordance with our cross-sectional design.
4. **Sample Size Justification**
  ➤ *Action taken:* We added a sample size justification using GPower software (effect size ρ = 0.30, α = 0.05, power = 80%), supporting the adequacy of the chosen sample (n = 72).
5. **Availability of Full Instruments**
  ➤ *Action taken:* The complete versions of the OHIP-14 and HU-DBI (Spanish version) were uploaded to Zenodo, along with their validation matrices and reliability reports.
  - Dataset: https://doi.org/10.5281/zenodo.14847738
  - Methodological Annex: https://doi.org/10.5281/zenodo.15236712
6. **Overstatement and Global Generalization**
  ➤ *Action taken:* We revised the Discussion and Conclusion sections to avoid overgeneralizing results. We emphasized the local scope and acknowledged methodological limitations.
7. **Clarification between General QoL and OHRQoL**
  ➤ *Action taken:* We explicitly distinguished **Oral Health-Related Quality of Life (OHRQoL)** from general Quality of Life (QoL), clarifying its role as the dependent variable in our analysis.
8. **Title Modification**
  ➤ *Action taken:* In response to your and Reviewer 1’s suggestions, the original title “Impact of oral health perception on quality of life” was revised to:
**“Relationship between the perception of oral health and the quality of life of hospital staff”**,
  in order to better reflect the non-causal, correlational nature of the study.

We trust these revisions meet your expectations and contribute to the improved quality and clarity of the manuscript. Once again, we thank you for your valuable insights and support.

 Sincerely,

**Miryam Griselda Lora Loza**

 (Corresponding Author)

# Response

**Published:** 2025-07-04

**Authors:** mlora@ucv.edu.pe Lora Loza

**Affiliations:** La Libertad, Universidad Cesar Vallejo Facultad de Ciencias Medicas, Trujillo /Trujillo, Trujillo, Peru

**Manuscript Title:** *Association between Oral Health Perception and Oral Health-Related Quality of Life among Hospital Staff*

**Corresponding Author:** Dr. Miryam Griselda Lora Loza

**Date:** July 4, 2025

 We sincerely thank Reviewer 1, Dr. Faris Yahya I. Asiri, for his constructive and thoughtful observations. Below, we provide detailed responses to each of his comments, along with the specific sections where changes were made in the revised manuscript. All edits are clearly marked in the tracked version.

**1. Study objectives and rationale – Clarify the purpose and design**

**Comment:**

 The objective of the study and the rationale need to be more clearly presented in the introduction and methods.

**Response:**

 We appreciate this valuable observation. The objective was reformulated to explicitly state that the study aims to examine the association between oral health perception and oral health-related quality of life (OHRQoL) among hospital staff. We also clarified the study design as correlational and cross-sectional in both the Introduction and the Methods sections.

*Changes made in:* Introduction (last paragraph), Methods – Design subsection.

**2. Sampling method and population – Explain selection criteria**

**Comment:**

 Please provide more details about the population selection and inclusion/exclusion criteria.

**Response:**

 We added detailed information about the inclusion and exclusion criteria and specified that the sample was selected by non-probabilistic convenience sampling from staff working at a level II hospital in northern Peru. The rationale for this selection was also explained.

*Changes made in:* Methods – Population and Sampling section.

**3. Instruments – Provide psychometric information**

**Comment:**

 More information is needed on the validity and reliability of the instruments used.

**Response:**

 We provided complete psychometric information for both the OHIP-14 and HU-DBI instruments. The OHIP-14 has been widely validated in Spanish-speaking populations. For the HU-DBI, we clarified that its Spanish version was adapted through expert judgment for content validity, and internal consistency was calculated using Cronbach’s alpha. All related evidence and matrices are now available in Appendix 1 and open-access via Zenodo.

*Changes made in:* Methods – Instruments subsection; Supplementary Appendix 1; References (Zenodo DOI: https://doi.org/10.5281/zenodo.15236712).

**4. Statistical analysis – More clarity needed**

**Comment:**

 Clarify how the variables were analyzed and justify the tests used.

**Response:**

 We improved the description of the statistical analysis. We explained the use of Spearman’s correlation due to non-normal data distribution, and logistic regression to explore the association between OHP and OHRQoL domains. Confidence intervals (95%) were added to support the strength and precision of the results.

*Changes made in:* Methods – Data Analysis subsection; Results – Table 3 and text.

**5. Interpretation of findings – Avoid causal language**

**Comment:**

 The interpretation of findings should not suggest causality in a cross-sectional design.

**Response:**

 We revised the Discussion and Conclusion sections to avoid any causal implications, consistently using associative and correlational terminology. We acknowledge the limitations of cross-sectional studies and emphasized the need for further longitudinal research.

*Changes made in:* Discussion and Conclusion sections.

**6. Ethical considerations – Add details on approvals**

**Comment:**

 Please confirm ethical approval and participant consent.

**Response:**

 We added that the study received ethical approval from the Institutional Ethics Committee of Universidad César Vallejo (Approval No. 007-2025-CEI), and that written informed consent was obtained from all participants in accordance with the Declaration of Helsinki.

*Changes made in:* Methods – Ethical Considerations subsection.

 We thank Reviewer 1 for his invaluable contributions. His remarks allowed us to strengthen the scientific robustness, ethical transparency, and methodological consistency of our study.

**Respectfully,**

**Dr. Miryam Griselda Lora Loza**

*On behalf of all co-authors*

# Reviewer report: Reviewer response for version 1

**DOI:** 10.5256/f1000research.177142.r371448

**Published:** 2025-03-24

**Authors:** Mike T John

**Affiliations:** 1 University of Minnesota, Minneapolis, USA

The manuscript “Impact of oral health perception on the quality of life of hospital staff” aimed to “analyze the relationship between the QoL and the PSB of the staff of a level II-1 hospital, located in the north of Peru, taking into account its dimensions and interactions. The research aims to provide evidence to design strategies that promote the well-being of health personnel and optimize the quality of care provided to patients.”

 The topic is interesting and relevant, but the methodology is flawed. The researchers are interested to “provide evidence to design strategies that promote the well-being of health personnel and optimize the quality of care provided to patients.” However, the authors performed a cross-sectional study, and this study design is not able to derive “evidence” for “promotion strategies.” Such evidence can only be derived from longitudinal studies.

**Is the work clearly and accurately presented and does it cite the current literature? – No**

 The authors are not aware that OHRQoL measured by OHIP does not have 7 dimensions. OHRQoL has four dimensions (John MT, 2020 [Ref-1]). 

 The authors are not aware of the recent scoring recommendations for OHIP (John MT., 2022 [Ref-2]).

**Is the study design appropriate and is the work technically sound? – No**

 The authors performed a cross-sectional study when a longitudinal study would have been necessary to answer the research question.

**Are sufficient details of methods and analysis provided to allow replication by others? – No**

 The central study outcome is oral health-related quality of life measured by OHIP-14.  The authors provided the following information: “Regarding the instruments, the Quality of Life (QoL) Questionnaire was used, originally designed by Slade and Spencer in 1994, adapted by Espinoza in 2017, and updated and validated in 2024 by Cruzado, Alvarado, and Lora. Loza. This questionnaire includes 14 items organized into 7 dimensions (Functional limitation, Physical pain, Psychological discomfort, Physical disabilities, Psychological disabilities, Social disability, and Disability), with 2 items per dimension. A 5-point Likert scale was applied (0 = never, 4 = very frequently), the results of which were classified into three categories: excellent (0-2 points), average (3-9 points), and poor quality of life (10 points or more).”

 The authors provided in the reference list a reference to Slade and Spencer and to Espinoza; however, a reference to “updated and validated in 2024 by Cruzado, Alvarado, and Lora. Loza” is missing. Therefore, it is not clear what was measured, especially, because the authors use an item response scale ranging from “never” to “very frequently,” which is not OHIP’s original response format.

**If applicable, is the statistical analysis and its interpretation appropriate? – No**

 For many results, e.g., “significant association between quality of life and oral health perception (Rho=0.391, p<0.05)”, the authors only provided point estimates. Confidence intervals around point estimates describe the uncertainty around the results. In this study with N=72, this uncertainty is substantial. Calculating a confidence interval according to https://www.statskingdom.com/correlation-confidence-interval-calculator.html reveals that correlations as small as 0.18 and as large as 0.57 are compatible with the study. While the observed correlation of 0.391 is statistically significant, i.e., it can be differentiated from 0, the observed “medium” size correlation could be “small” or “large” when the study would be repeated, indicating that not much can be learned from the study.

**Are the conclusions drawn adequately supported by the results? – No**

 The authors concluded “The link between quality of life and the perception of oral health in hospital staff emphasizes the need to implement comprehensive strategies that optimize their well-being and work performance, contributing significantly to improving hospital services.”

 First, the authors make a statement about quality of life. However, quality of life (a construct that entails environment, safety, freedom etc.) was not measured. ORAL HEALTH-RELATED quality of life was measured.

 Second, the magnitude of the link between oral health-related quality of life and perception of oral health is not clear. It could be small, i.e., not clinically relevant.

 Third, “the need to implement comprehensive strategies” is not supported by the cross-sectional study design.

Is the work clearly and accurately presented and does it cite the current literature?

No

If applicable, is the statistical analysis and its interpretation appropriate?

No

Are all the source data underlying the results available to ensure full reproducibility?

Yes

Is the study design appropriate and is the work technically sound?

No

Are the conclusions drawn adequately supported by the results?

No

Are sufficient details of methods and analysis provided to allow replication by others?

No

Reviewer Expertise:

oral health-related quality of life

I confirm that I have read this submission and believe that I have an appropriate level of expertise to state that I do not consider it to be of an acceptable scientific standard, for reasons outlined above.

## References

[1] : Foundations of oral health-related quality of life. *J Oral Rehabil* .2020; 10.1111/joor.13040 10.1111/joor.13040 32658317

[2] : STANDARDIZATION OF DENTAL PATIENT-REPORTED OUTCOMES MEASUREMENT USING OHIP-5 - VALIDATION OF "RECOMMENDATIONS FOR USE AND SCORING OF ORAL HEALTH IMPACT PROFILE VERSIONS". *J Evid Based Dent Pract* .2022;22(1S) : 10.1016/j.jebdp.2021.101645 101645 10.1016/j.jebdp.2021.101645 35063174 PMC9123939

# Response

**Published:** 2025-05-29

**Authors:** mlora@ucv.edu.pe Lora Loza

**Affiliations:** La Libertad, Universidad Cesar Vallejo Facultad de Ciencias Medicas, Trujillo /Trujillo, Trujillo, Peru

**Dear Dr. Mike T. John,**

 Warm greetings.

 I wish to express my deepest gratitude for the time and expertise you invested in reviewing our manuscript. Your thorough and constructive feedback has been instrumental in guiding substantial improvements to our study entitled:

**“Relationship between oral health perception and quality of life in hospital staff.”**

 I humbly apologize for not responding sooner to your detailed review. At the time, I was not yet familiar with the F1000Research platform and unfortunately overlooked your comments. Now that I have a better understanding of the system, I wish to respectfully inform you that **all your major observations have now been addressed** in the revised version, including:

1. **Title and Causal Language**: The word *“impact”* was replaced with *“relationship”*, and causal or prescriptive expressions were carefully revised to remain within the scope of a correlational, cross-sectional design.
2. **Clarification of OHIP-14 Scoring**: We corrected the dimensional interpretation and cited John (2020, 2022) for standard scoring guidance. The questionnaire was reformulated to respect international standards and now includes proper acknowledgment of the four-dimensional structure of OHRQoL.
3. **Psychometric Validity**: We uploaded the full instruments (OHIP-14 and HU-DBI) including psychometric data to **Zenodo**, ensuring full transparency and replicability.
4. **Statistical Reporting**: We added confidence intervals for correlation coefficients and justified the sample size using G*Power, as well as reporting Nagelkerke’s pseudo R² to better interpret the logistic regression outcomes.
5. **Terminology**: We replaced the term “quality of life” with “oral health-related quality of life” throughout the manuscript to maintain conceptual accuracy.
6. **Conclusions Revised**: We rephrased our conclusions to reflect the statistical scope and limitations of a cross-sectional study, without suggesting implementation of strategies not directly derived from the design.

These modifications were made not only out of respect for your review, but also in appreciation for your high standards in the field of oral health-related quality of life research. We believe that the manuscript now meets the criteria for methodological and scientific integrity and would be deeply honored if you could consider revisiting the updated version.

 Please feel free to share any further suggestions—we remain open and grateful to continue improving.

 With highest respect and appreciation,

**Miryam Griselda Lora Loza**

*Corresponding Author*

# Response

**Published:** 2025-11-12

**Authors:** mlora@ucv.edu.pe Lora Loza

**Affiliations:** La Libertad, Universidad Cesar Vallejo Facultad de Ciencias Medicas, Trujillo /Trujillo, Trujillo, Peru

Dear Dr. John, as a brief update: Version 5 integrates your core recommendations—OHIP conceptualization (four dimensions) and scoring aligned with John (2020; 2022), non-causal language for a cross-sectional design, **95% CIs** added to correlations, and instruments/data shared openly. We appreciate your foundational input and would be grateful for any further advice. All changes are visible in V5 (tracked).
